# Active reinforcement learning versus action bias and hysteresis: control with a mixture of experts and nonexperts

**DOI:** 10.1371/journal.pcbi.1011950

**Published:** 2024-03-29

**Authors:** Jaron T. Colas, John P. O’Doherty, Scott T. Grafton

**Affiliations:** 1 Department of Psychological and Brain Sciences, University of California, Santa Barbara, California, United States of America; 2 Division of the Humanities and Social Sciences, California Institute of Technology, Pasadena, California, United States of America; 3 Computation and Neural Systems Program, California Institute of Technology, Pasadena, California, United States of America; Ecole Normale Superieure, FRANCE

## Abstract

Active reinforcement learning enables dynamic prediction and control, where one should not only maximize rewards but also minimize costs such as of inference, decisions, actions, and time. For an embodied agent such as a human, decisions are also shaped by physical aspects of actions. Beyond the effects of reward outcomes on learning processes, to what extent can modeling of behavior in a reinforcement-learning task be complicated by other sources of variance in sequential action choices? What of the effects of action bias (for actions per se) and action hysteresis determined by the history of actions chosen previously? The present study addressed these questions with incremental assembly of models for the sequential choice data from a task with hierarchical structure for additional complexity in learning. With systematic comparison and falsification of computational models, human choices were tested for signatures of parallel modules representing not only an enhanced form of generalized reinforcement learning but also action bias and hysteresis. We found evidence for substantial differences in bias and hysteresis across participants—even comparable in magnitude to the individual differences in learning. Individuals who did not learn well revealed the greatest biases, but those who did learn accurately were also significantly biased. The direction of hysteresis varied among individuals as repetition or, more commonly, alternation biases persisting from multiple previous actions. Considering that these actions were button presses with trivial motor demands, the idiosyncratic forces biasing sequences of action choices were robust enough to suggest ubiquity across individuals and across tasks requiring various actions. In light of how bias and hysteresis function as a heuristic for efficient control that adapts to uncertainty or low motivation by minimizing the cost of effort, these phenomena broaden the consilient theory of a mixture of experts to encompass a mixture of expert and nonexpert controllers of behavior.

## Author summary

Reinforcement learning unifies neuroscience and AI with a universal computational framework for motivated behavior. Humans and robots alike are active and embodied agents who physically interact with the world and learn from feedback to guide future actions while weighing costs of time and energy. Initially, the modeling here attempted to identify learning algorithms for an interactive environment structured with patterns in counterfactual information that a human brain could learn to generalize. However, behavioral analysis revealed that a wider scope was necessary to identify individual differences in not only complex learning but also action bias and hysteresis. Sequential choices in the pursuit of rewards were clearly influenced by endogenous action preferences and persistent bias effects from action history causing repetition or alternation of previous actions. By modeling a modular brain as a mixture of expert and nonexpert systems for behavioral control, a distinct profile could be characterized for each individual attempting the experiment. Even for actions as simple as button pressing, effects specific to actions were as substantial as the effects from reward outcomes that decisions were supposed to follow from. Bias and hysteresis are concluded to be ubiquitous and intertwined with processes of active reinforcement learning for efficiency in behavior.

## Introduction

Whether in machine learning and artificial intelligence or in animal learning and neural intelligence, the most crucial portion of reinforcement learning (RL) [1–3] is not passive, offline, or observational but instead active and online with a challenge of not only prediction but also real-time control. In the real world, resources for activity are finite, and much of active RL is also embodied RL. Whether robot or human, the embodied agent learns from feedback to make decisions and select physical actions that maximize future reward while minimizing various costs of energy as well as time.

**Fig 1:**
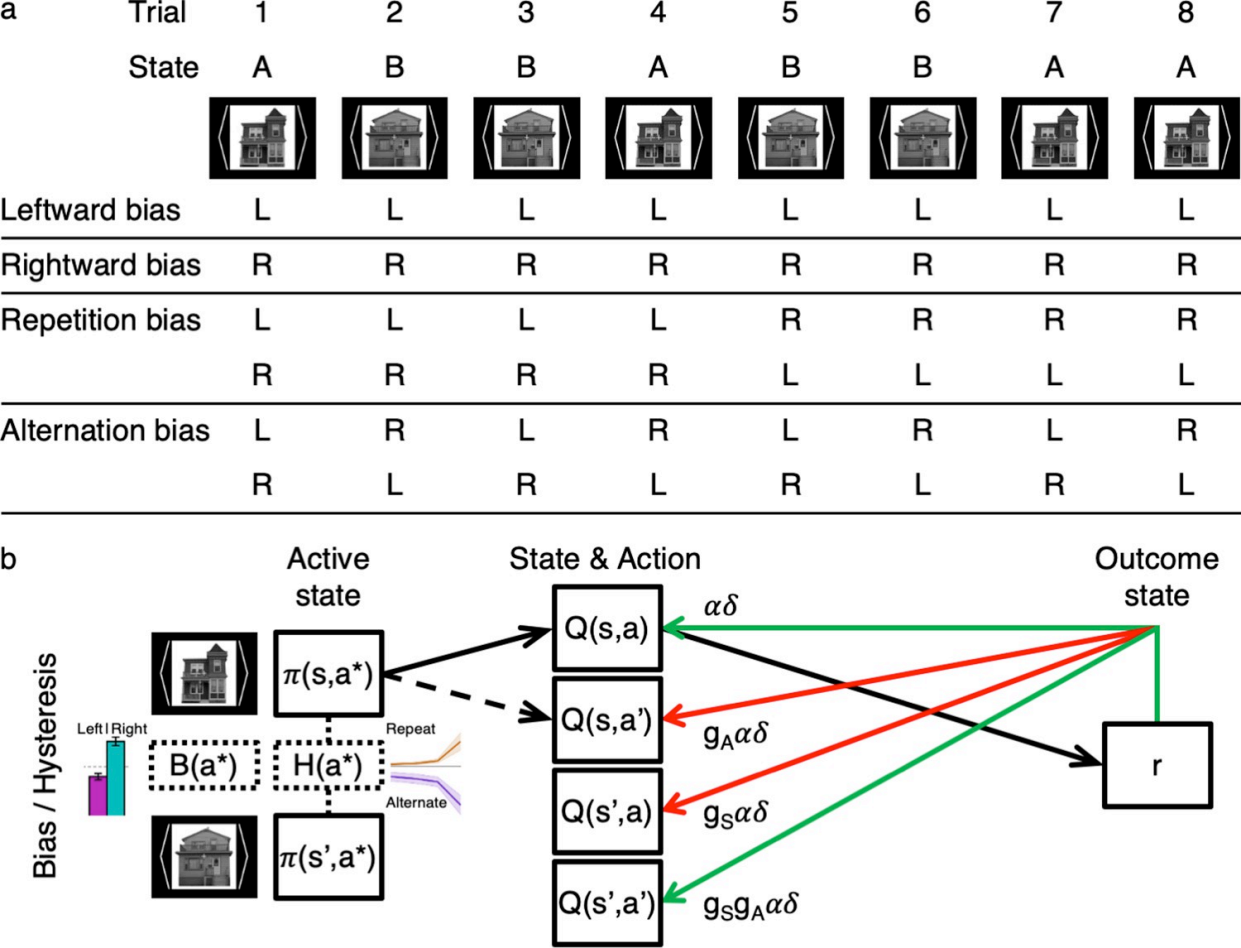
Action bias and hysteresis for the “generalized reinforcement learning” (GRL) model. **(a)** Each trial of the structured reward-learning task was initiated with an image cue symbolizing the state of the environment (e.g., “A” or “B”), where the optimal action given the state was a button press with either the left (“L”) or right (“R”) hand. In contrast to the expert control of GRL for mapping state-action pairs to rewards, the nonexpert forces of action bias and hysteresis were modeled as leftward or rightward bias and repetition or alternation bias. These action-specific effects manifest independently of the external state and reward history. **(b)** What matters for the present purposes is that, while a model with GRL adds complexity to basic RL, even more complexity must be accommodated for action bias and hysteresis. The agent’s mixture policy *π*_*t*_*(s*_*t*_,a*) is probabilistic over available actions a* in state *s*_*t*_. The action selection of this mixture policy is determined by not only learned value for state-action pairs *Q*_*t*_*(s*_*t*_,a*) but also constant bias B(a*) and dynamic hysteretic bias *H*_*t*_(a*) with an exponentially decaying hysteresis trace. The outcome of the chosen action *a*_*t*_ is a reward *r*_*t+1*_ that updates *Q*_*t*_*(s*_*t*_,*a*_*t*_*)* via the reward-prediction error (RPE) *δ*_*t+1*_ weighted by a learning rate *α*. For GRL specifically, this RPE signal is generalized to representations of other state-action pairs according to extra parameters for action generalization (*g*_*A*_) and state generalization (*g*_*S*_). See Figs 8 and 13 for details of the plots representing individual differences in constant lateral bias (left versus right) and the exponential hysteresis trace (repeat versus alternate). See also the original report of this study with additional details about the paradigm and GRL per se [12].

The RL framework has appreciable predictive validity [4,5] when accounting for human choices and learning behavior in a variety of settings [6–8]—let alone the power of extensions of RL [9–12]. However, such models sometimes fail to account well for an individual’s behavior even in a relatively simple task that should be amenable to RL in principle [13]. An open question concerns whether other components of variance not based on learning also exist alongside RL so as to collectively provide a better account of motivated behavior and even learning itself within a more comprehensive model. The present study focuses on the contributions of other elements of active learning that are also essential in their own way: action bias—specifically for actions per se—and action hysteresis, which is determined by the history of previously selected actions (**Fig 1A**).

The present case of two available actions (one per hand) reduces the first component of action bias to a single bidirectional constant for left versus right [14–16]. Hysteresis is bidirectional as well and adds dynamics in the form of either repetition or alternation of previous actions, which may also manifest for a horizon beyond just the most recent action [17–20]. Despite at least some precedent for either action bias or action hysteresis (more so the latter), the combination of both bias and hysteresis has even less precedent for RL [12,21].

The standard setup for fitting RL to behavior (e.g., [22]) begins with a 2-parameter model tuned for the learning rate and the softmax temperature, where the latter represents stochasticity [3,23–25]. This base model is then built upon with additional free parameters to test for more complex learning phenomena, which should include the due diligence of model comparison and qualitative falsification [26–28]. However, an alternative line of questioning could instead begin with asking whether more parsimonious and perhaps more substantial sources of variance merit prioritization before making any new assumptions about complexities within learning. The emphasis can also be shifted away from the prescriptive (i.e., “According to some notion of ‘optimality’, what should a person do here?”) in favor of the descriptive (“What do people actually do here?”) while creating an opportunity to circle back from empirical findings to a new perspective on different aspects of optimality in behavior.

In practice, model fitting is nontrivial with a sequence of choices typically limited to hundreds or even just dozens of observations. Adding to this challenge, increasingly complex behavior under study imposes greater demands for accommodating multidimensional individual differences and optimizing individual fits without hierarchical Bayesian fitting [13,29] and its disadvantage of estimation bias [30–35]. (For a random grouping of independent data sets, even hierarchical fitting compromises their independence with the strong assumption of a common distribution for every individual based on the ecological fallacy [36–38].) Both within and between individual sequences, sources of variance other than RL may be crucial to complement an RL model despite the costs of additional degrees of freedom. In other words, including modules beyond RL in a model of actual behavior can alleviate estimation bias and other distortions of learning parameters that would otherwise be forced to simultaneously fit other phenomena with omitted variables.

In the present study, we hypothesize that behavior during active learning is determined not only by RL and stochasticity but also by action bias and hysteresis, which are independent of the current state of the external environment and its reward history (**Fig 1**). This state-independent hysteresis in particular makes actions depend on previous actions regardless of states, but state-dependent hysteresis was also considered later (**Table 1**). The interplay of these different forces was investigated for human behavior in a task that in one sense is a hierarchical reversal-learning task but in another sense is a sequential button-pressing task (**Fig A in S1 Text**). Hence the behavioral data of a multisite neuroimaging study reported previously [12] were reanalyzed with further model comparison from this bias-centric perspective.

**Table 1:** Variables for basic forms of RL, bias, and hysteresis. Fundamentally for even basic RL, the possibilities for variables in a more comprehensive behavioral model can be classified according to dependence on (or independence of) states, actions. previous actions, and reward outcomes. In principle, whereas action value is outcome-dependent, action hysteresis is outcome-independent. However, when modeling actual behavior, this conceptual independence does not guarantee statistical independence because of incidental correlations in finite sequences of action choices. For the present study, the primary model comparison focuses on the three variables (marked with an asterisk) that are the most fundamental and typically the most dissociable—namely, constant bias *B(a)*, state-independent action hysteresis *H(a)*, and state-dependent action value *Q(s*,*a)*. The extended model comparison also incorporates state-dependent action hysteresis *H(s*,*a)* and state-independent action value *Q(a)*. Note that state value *V(s)* is generally relevant in RL but is not considered here. The abbreviations “PrevAction”, “dep.”, and “indep.” correspond to “previous action”, “dependent”, and “independent”, respectively.

Variable	Term	Action-	PrevAction-	State-	Outcome-
Constant action bias*	B(a)	dep.	indep.	indep.	indep.
State-independent action hysteresis*	H(a)	dep.	dep.	indep.	indep.
State-dependent action hysteresis	H(s,a)	dep.	dep.	dep.	indep.
State-independent action value	Q(a)	dep.	indep.	indep.	dep.
State-dependent action value*	Q(s,a)	dep.	indep.	dep.	dep.
State value	V(s)	indep.	indep.	dep.	dep.

Too often, such action-specific effects have been overlooked altogether or given only cursory mention as if they were inconsequential in the context of a learning model. If considered at all, the scope of hysteresis has also usually been limited to only one trial back. (To address this issue here, we modeled hysteresis over a time horizon longer than one trial.) Moreover, because repetition tends to predominate in aggregate behavior for RL and other sequential paradigms, manifestations of hysteresis have mostly been framed so as to deemphasize or entirely disregard alternation biases in favor of repetition biases. Autocorrelational effects have thus been referred to in the literature with unidirectional and often imprecise terminology such as “perseveration”, “perseverance” (a misnomer), “persistence”, “habit”, “choice stickiness”, “choice consistency”, “repetition priming”, “response inertia”, or “behavioral momentum”. Semantics of interpretation aside, the common thread for hysteresis is a past action’s influence on an upcoming action with independence from learnable external feedback and typically, albeit not necessarily, from external states as well.

A more comprehensive model of action selection can also enhance identifiability with respect to actual learning (or lack thereof) as opposed to other components of variance that may mimic or otherwise obscure signatures of learning with spurious correlations across the finite sequence of actions [17,18,27,28,39–47]. As external reinforcement promotes consistent repetition of responses within a state, so too can action bias, and both repetition and alternation from hysteresis can coincidentally align with the reward contingencies of the sequence of states. Whereas preexisting constant biases interact with learning when base rates for actions are unbalanced in sequence, hysteretic biases can further complicate action sequences with not only intrinsic dynamics but also more possibilities for interactions across any sequential patterns in the environment and the dynamics of learning.

Perhaps surprisingly, the hypothesis for hysteresis in the present experiment was that alternation would predominate rather than repetition. An action policy biased toward alternation would follow from the fact that, by design, choosing actions optimally in response to the rotating states of this environment would result in alternating more frequently. Yet, by design, this perseverative alternation that is characteristically independent of learned external value was therefore not conducive to obtaining more rewards from this environment.

The primary model comparison here (**Table 2 and Table A in S1 Text**) exhaustively tested various combinations of action-specific effects as well as “generalized reinforcement learning” (GRL), which is a quasi-model-based extension of model-free RL that can flexibly generalize value information across states and actions (**Fig 1B and Fig B in S1 Text**) [12]. GRL per se is somewhat incidental for the present purposes, but what matters as far as a test case here is that a model incorporating the complexities of bias and hysteresis should still be amenable to exploring complex learning algorithms beyond the most basic RL. GRL is especially complicating in this regard because it introduces high-frequency dynamics to learning with counterfactual updates of multiple value representations in parallel.

**Table 2:** Model parameters (condensed). Free parameters are listed for the 72 behavioral models in ascending order of complexity within and across classes. The models are coded with the first letter of the label referring to four possibilities: an absence of learning (“X”), reinforcement learning (RL) without generalization (“0”), generalized reinforcement learning (GRL) with one shared generalization parameter *g*_*1*_ (“1”), or GRL with two separate generalization parameters *g*_*1*_ and *g*_*2*_ (“2”). RL itself required free parameters for the learning rate *α* and the softmax temperature *τ*. Models labeled with “C” for the second letter included a constant lateral bias, which was arbitrarily designated as a rightward bias *β*_*R*_ (where *β*_*R*_ < 0 is leftward). The list is condensed with bracket notation to represent the range for the *n*-back horizons of each successive model within a hysteresis category (e.g., “2CE[1–3]” for models 2CE1, 2CE2, and 2CE3). Models labeled with”N” and ending with a positive integer (from the range in brackets) included *n*-back hysteresis with free parameters *β*_*n*_ for repetition (*β*_*n*_ > 0) or alternation (*β*_*n*_ < 0) of each previous action represented—up to 4 trials back (*β*_*4*_) with learning and up to 8 trials back (*β*_*8*_) without learning. Models labeled with “E” and ending with a positive integer *N* (from the range in brackets) included exponential hysteresis with inverse decay rate *λ*_*H*_ taking effect *N+1* trials back. Exponential models could also be both parametric and nonparametric with *N* free parameters *β*_*n*_ for initial *n*-back hysteresis up to 3 trials back (*β*_*3*_), where the final *β*_*N*_ is the initial magnitude of the exponential component. “df” stands for degrees of freedom. See also Table A in S1 Text for the unrolled version of the list. This ordering of the models corresponds to the ordering in Figs 2 and 3.

		RL		GRL		Bias	Hysteresis								
Model	df	*α*	*τ*	*g*_*1*_	*g*_*2*_	*β*_*R*_	*λ*_*H*_	*β*_*1*_	*β*_*2*_	*β*_*3*_	*β*_*4*_	*β*_*5*_	*β*_*6*_	*β*_*7*_	*β*_*8*_
X	0	-	-	-	-	-	-	-	-	-	-	-	-	-	-
XC	1	-	-	-	-	*β*_*R*_	-	-	-	-	-	-	-	-	-
XN[1–8]	1–8	-	-	-	-	-	-	*β*_*1*_	*β*_*2*_	*β*_*3*_	*β*_*4*_	*β*_*5*_	*β*_*6*_	*β*_*7*_	*β*_*8*_
XCN[1–8]	2–9	-	-	-	-	*β*_*R*_	-	*β*_*1*_	*β*_*2*_	*β*_*3*_	*β*_*4*_	*β*_*5*_	*β*_*6*_	*β*_*7*_	*β*_*8*_
XE[1–3]			2–4	-	-	-	-	-	*λ*_*H*_	*β*_*1*_	*β*_*2*_	*β*_*3*_	-	-	-	-	-
XCE[1–3]	3–5	-	-	-	-	*β*_*R*_	*λ*_*H*_	*β*_*1*_	*β*_*2*_	*β*_*3*_	-	-	-	-	-
0	2	*α*	*τ*	-	-	-	-	-	-	-	-	-	-	-	-
0C	3	*α*	*τ*	-	-	*β*_*R*_	-	-	-	-	-	-	-	-	-
0N[1–4]	3–6	*α*	*τ*	-	-	-	-	*β*_*1*_	*β*_*2*_	*β*_*3*_	*β*_*4*_	-	-	-	-
0CN[1–4]	4–7	*α*	*τ*	-	-	*β*_*R*_	-	*β*_*1*_	*β*_*2*_	*β*_*3*_	*β*_*4*_	-	-	-	-
0E[1–3]	4–6	*α*	*τ*	-	-	-	*λ*_*H*_	*β*_*1*_	*β*_*2*_	*β*_*3*_	-	-	-	-	-
0CE[1–3]	5–7	*α*	*τ*	-	-	*β*_*R*_	*λ*_*H*_	*β*_*1*_	*β*_*2*_	*β*_*3*_	-	-	-	-	-
1	3	*α*	*τ*	*g*_*1*_	-	-	-	-	-	-	-	-	-	-	-
1C	4	*α*	*τ*	*g*_*1*_	-	*β*_*R*_	-	-	-	-	-	-	-	-	-
1N[1–4]	4–7	*α*	*τ*	*g*_*1*_	-	-	-	*β*_*1*_	*β*_*2*_	*β*_*3*_	*β*_*4*_	-	-	-	-
1CN[1–4]	5–8	*α*	*τ*	*g*_*1*_	-	*β*_*R*_	-	*β*_*1*_	*β*_*2*_	*β*_*3*_	*β*_*4*_	-	-	-	-
1E[1–3]	5–7	*α*	*τ*	*g*_*1*_	-	-	*λ*_*H*_	*β*_*1*_	*β*_*2*_	*β*_*3*_	-	-	-	-	-
1CE[1–3]	6–8	*α*	*τ*	*g*_*1*_	-	*β*_*R*_	*λ*_*H*_	*β*_*1*_	*β*_*2*_	*β*_*3*_	-	-	-	-	-
2	4	*α*	*τ*	*g*_*1*_	*g*_*2*_	-	-	-	-	-	-	-	-	-	-
2C	5	*α*	*τ*	*g*_*1*_	*g*_*2*_	*β*_*R*_	-	-	-	-	-	-	-	-	-
2N[1–4]	5–8	*α*	*τ*	*g*_*1*_	*g*_*2*_	-	-	*β*_*1*_	*β*_*2*_	*β*_*3*_	*β*_*4*_	-	-	-	-
2CN[1–4]	6–9	*α*	*τ*	*g*_*1*_	*g*_*2*_	*β*_*R*_	-	*β*_*1*_	*β*_*2*_	*β*_*3*_	*β*_*4*_	-	-	-	-
2E[1–3]	6–8	*α*	*τ*	*g*_*1*_	*g*_*2*_	-	*λ*_*H*_	*β*_*1*_	*β*_*2*_	*β*_*3*_	-	-	-	-	-
2CE[1–3]	7–9	*α*	*τ*	*g*_*1*_	*g*_*2*_	*β*_*R*_	*λ*_*H*_	*β*_*1*_	*β*_*2*_	*β*_*3*_	-	-	-	-	-

Previously, the GRL model was built with fixed prior assumptions for another three free parameters representing action bias and hysteresis. One of these parameters specifies the constant lateral bias; the other two specify a decaying exponential function for the hysteresis trace extending backward across the sequence. This particular configuration of constant bias and exponential hysteresis was initially arrived at intuitively more so than empirically [12,21] while drawing elements from earlier models [17,18]. Now, the 3-parameter adjunct was to actually be tested against GRL alone as well as both simpler and more complex variations for bias and (state-independent) hysteresis. Subsequent testing also proceeded to alternative model features that could be other sources of action repetition or alternation, including state-dependent hysteresis, state-independent action value, confirmation bias in learning, or asymmetric learning rates more generally.

Abiding by Occam’s razor [48], the more parsimonious factors of action bias and hysteresis should be granted first priority for inclusion if they are sufficiently substantial, but testing empirical data was necessary to verify practical feasibility in consideration of the compounded complexity with different forms of learning. Individuals found to not learn well were expected to reveal the greatest effects of bias and hysteresis. Yet those who learned accurately were also hypothesized to exhibit biases that would account for significant variance (even if this were to amount to less variance than that from learning).

To the end of establishing guidelines for behavioral modeling in general, there were further questions concerning how exactly these directional biases would manifest and how substantial they would be for the experimenter’s default choice of pressing a button, which is a simple and familiar action with trivial motor demands. For proof of concept, the present paradigm can query not only the suitability of these particular forms of biases for button presses but also the viability of these factors as additional complexities while learning theory is advanced. With reference to analogous architectures in machine learning [49–54] as well as with general appeal to modular parallelism and conditional computation for balancing versatility and efficiency in optimal control, the consilient theory of a mixture of experts [6–8,55–57] can be broadened further for a mixture of expert and nonexpert controllers of behavior (see Discussion). This contrast of expertise versus efficiency is represented here by different types of expert RL versus nonexpert bias and hysteresis.

## Results

### Paradigm

Additional details of the study and previous results can be found in the original report for these data sets [12]. The hierarchical reversal-learning task delivered probabilistic outcomes for combinations of categorized states and contingent actions with reward distributions changing across 12 blocks of trials (**Figs A and B in S1 Text**). Suitably for first testing GRL, the state (or context) of each trial represented a two-armed contextual bandit belonging to one of two categories (e.g., faces or houses) with two anticorrelated states per category and two anticorrelated actions per state (i.e., left-hand button press or right-hand button press). For an optimal learner, the counterfactual information in this anticorrelational structure could be leveraged with the discriminative generalization of GRL. The action-generalization weight *g*_*A*_ and state-generalization weight *g*_*S*_, which would ideally both be negative for discriminative generalization, govern the relaying of the reward-prediction error across state-dependent actions or across states within a category, respectively.

For standard behavioral RL (with or without an extension such as GRL), the state-dependent action values *Q*_*t*_*(s*,*a)* that are learned over time would be the only inputs to a probabilistic action-selection policy *π*_*t*_*(s*,*a)* characterized by a softmax function with temperature *τ*:

$$\pi_{t}(s_{t},a)=P(a_{t}=a|s_{t})=\frac{\exp\left\{Q_{t}(s_{t},a)/\tau \right\}}{\sum_{a^{*}}\exp\left\{Q_{t}(s_{t},a^{*})/\tau \right\}}$$

As the scope of the model is expanded, the present study emphasizes that the action policy is a function of not only action value *Q*_*t*_*(s*,*a)* but also constant bias *B(a)* and dynamic hysteretic bias *H*_*t*_*(a)* as modules within a mixture of experts and nonexperts (**Fig 1**) [12,21]. Constant bias *B(a)* becomes a lateral bias between left and right actions in this case, whereas the dynamic hysteretic bias *H*_*t*_*(a)* maps repetition and alternation to positive and negative signs, respectively. To represent these action-specific biases that are independent of external state and reward history, the equation for the mixture policy incorporates additional terms like so:

$$\pi_{t}(s_{t},a)=\frac{\exp\left\{(Q_{t}(s_{t},a)+H_{t}(a)+B(a))/\tau \right\}}{\sum_{a^{*}}\exp\left\{(Q_{t}(s_{t},a^{*})+H_{t}(a^{*})+B(a^{*}))/\tau \right\}}$$

### Adding complexity in both learning and action bias and hysteresis

The primary model comparison here crossed factors for value-based learning (with first character “X”, “0”, “1”, or “2” for the model label), constant bias (“C”), *n*-back hysteresis (“N”), and exponential hysteresis (“E”) to incrementally build 72 models that were tested for each participant as an individual (**Table 2 and Table A in S1 Text**). Note that, in the original model comparison [12], the final 7-parameter model “2CE1” was built with two generalization parameters (*g*_*A*_ and *g*_*S*_) added to an initial 5-parameter base model “0CE1” (first adding *β*_*R*_, *β*_*1*_, and *λ*_*H*_ to the standard 2-parameter base model “0” with only learning rate *α* and temperature *τ*). Unlike the original factorial model comparison, the present model comparison was more exhaustive for biases rather than reduced variants of GRL or alternative learning algorithms. Hence the bias and hysteresis factors were presently crossed with the limited cases of no learning (“X”) (*α* = *g*_*A*_ = *g*_*S*_ = 0), basic RL (“0”) (*g*_*A*_ = *g*_*S*_ = 0), 1-parameter GRL (“1”) (*g*_*A*_ = min{0, *g*_*S*_}, -1 ≤ *g*_*S*_ ≤ 1), and 2-parameter GRL (“2”) (-1 ≤ *g*_*A*_ ≤ 0, -1 ≤ *g*_*S*_ ≤ 1).

The binary factor of constant bias was implemented as a lateral bias *β*_*R*_ (where a positive sign is arbitrarily rightward). Hysteresis, the next main factor, was further subdivided between exponential and *n*-back hysteresis as parametric and nonparametric alternatives, respectively. A model with *N*-back hysteresis included independent weights *β*_*n*_ for each of *N* total previous actions (the final number in the label such as the “1” in 2CN1 for 1-back), where each signed weight corresponds to a bias in favor of repetition (*β*_*n*_ > 0) or alternation (*β*_*n*_ < 0) of the respective previous action. The alternative of parametric hysteresis featured exponential decay (e.g., 2CE1) but could also include up to two additional degrees of freedom (e.g., up to 2CE3) for nonparametric weights on the most recent previous actions—that is, *n*-back and exponential hysteresis combined (cf. regression analyses in [17,20,58–61]).

Within each data set (i.e., the 3-T Face/House (“FH”) version or the 7-T Color/Motion (“CM”) version), the first step of the original analysis [12] entailed dividing participants into three subgroups according to model-independent performance on the task [18] as well as the results of model fitting [21]. A subset of participants was initially set aside as the “Good learner” (“G”) group (FH: *n* = 31/47, CM: *n* = 16/22) if choice accuracy was significantly greater than the chance level of 50% for a given individual (*p* < 0.05). The remaining participants—for whom the null hypothesis of chance accuracy could not be rejected at the individual level (*p* > 0.05)—were further subdivided between the “Poor learner” (“P”) group (FH: *n* = 9/47, CM: *n* = 5/22) and the “Nonlearner” (“N”) group (FH: *n* = 7/47, CM: *n* = 1/22) according to whether or not an RL or GRL model (including bias and hysteresis) could yield a significant improvement in goodness of fit relative to the pure bias-and-hysteresis model XCE1, which is nested within the full 2CE1 model adding GRL but has no sensitivity to reward or its omission.

Whereas the original model comparison [12] emphasized variants of GRL with associative or discriminative generalization and permuted these factors accordingly, the presently emphasized factors of action bias and hysteresis had been assumed a priori and fixed with three parameters for constant bias and exponential decay of the hysteresis trace. Although the original results were in favor of the 7-parameter 2CE1 model, these conclusions were drawn from only one perspective with fixed assumptions for action bias and hysteresis. That is, two new parameters for action and state generalization (*g*_*A*_, *g*_*S*_) were previously justified as additions to a 5-parameter base model 0CE1 starting with two parameters for basic RL (*α*, *τ*), one for constant bias (*β*_*R*_), and two for exponential hysteresis (*β*_*1*_, *λ*_*H*_). The 3-parameter adjunct (“-CE1”) was hypothesized to retain the most explanatory power post-correction in the present model comparison as well—even as various simpler and more complex alternatives were now being tested for due diligence.

**Fig 2:**
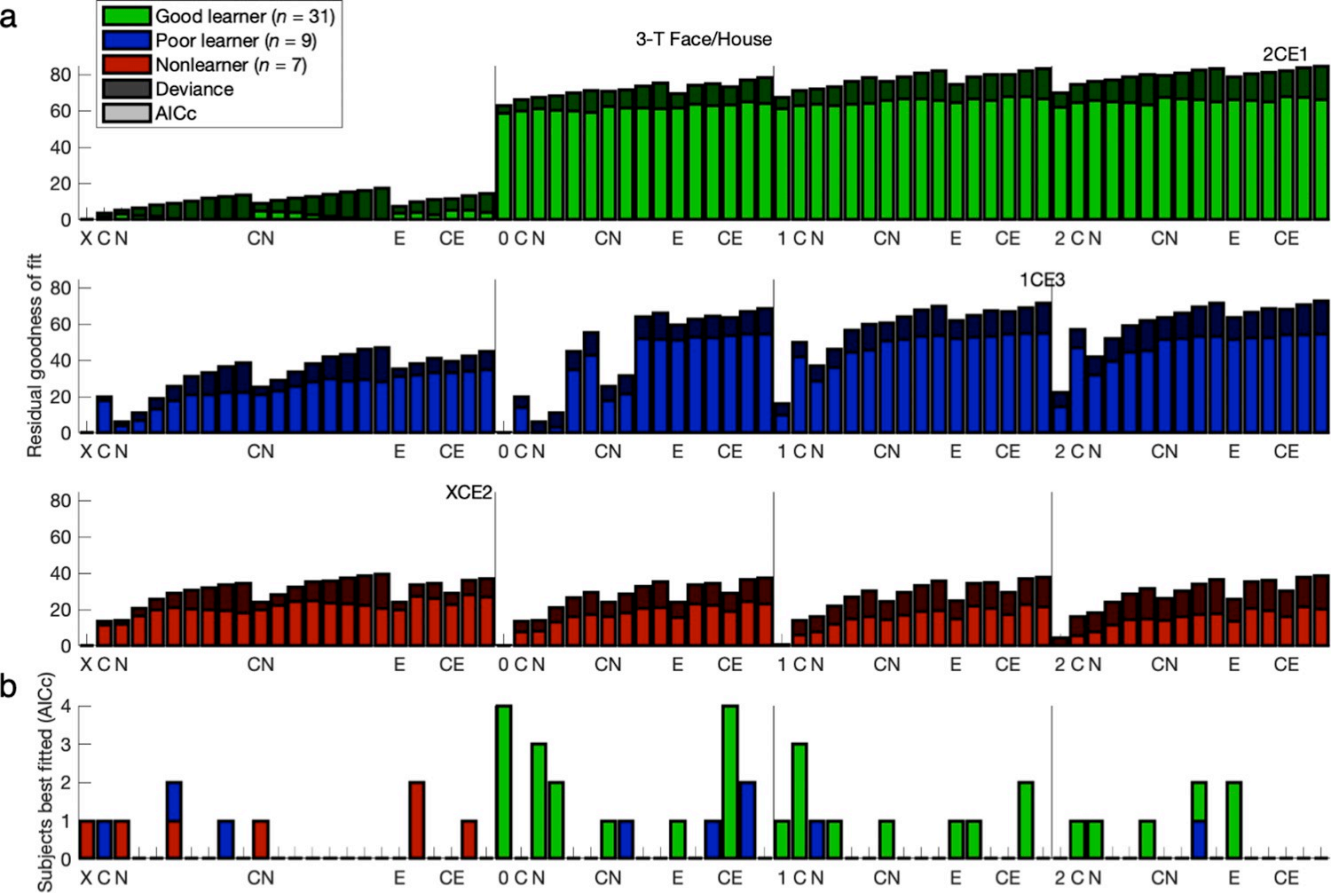
Model comparison: 3-T Face/House version. The ordering of the models here corresponds to the ordering in Table 2 and Table A in S1 Text. As before, the model begins with “X-”, “0-”, “1-”, or “2-” for no learning, basic RL, 1-parameter GRL, or 2-parameter GRL. A subsequent “C” denotes constant bias, and “N” or “E” represents *n*-back or exponential hysteresis, respectively, while incrementally adding a step back to the *n*-back horizon with each successive model within a hysteresis category (e.g., the rightmost models 2CE1, 2CE2, and 2CE3). **(a)** Shown for each model is average goodness of fit relative to the null chance model (“X”) with (light bars) and without (light and dark bars combined) a penalty for model complexity according to the corrected Akaike information criterion (AICc). With the addition of action bias and hysteresis parameters alongside GRL, Poor learners (blue bars) and Nonlearners (red bars) revealed the greatest gains in model performance, but Good learners (green bars) benefited significantly as well. The best-performing models (written above each plot) featured not only GRL for the actual learners but also constant bias and exponential hysteresis for all (FH-G: 2CE1, FH-P: 1CE3, FH-N: XCE2; see Fig 3 for CM-G: 2CE1, CM-P: 1CE2). For the most essential Good-learner group, the originally preferred 2CE1 model was validated as preferable to both simpler and more complex alternatives for the specification of bias and hysteresis or lack thereof. A more positive residual corresponds to a superior fit. **(b)** Counts of the participants best fitted by each model according to the AICc are plotted with separation of Good learners, Poor learners, and Nonlearners. At the individual level, 87% of participants across both data sets exhibited significant effects of some kind of action bias or hysteresis. The 7-parameter 2CE1 model—complementing 2-parameter GRL with constant bias and 2-parameter exponential hysteresis—accommodates heterogeneity in both learning and action-specific effects across individuals, leaving 64% best fit by 2CE1 or one of its nested models rather than other *n*-back or *n*-back-plus-exponential models.

**Fig 3:**
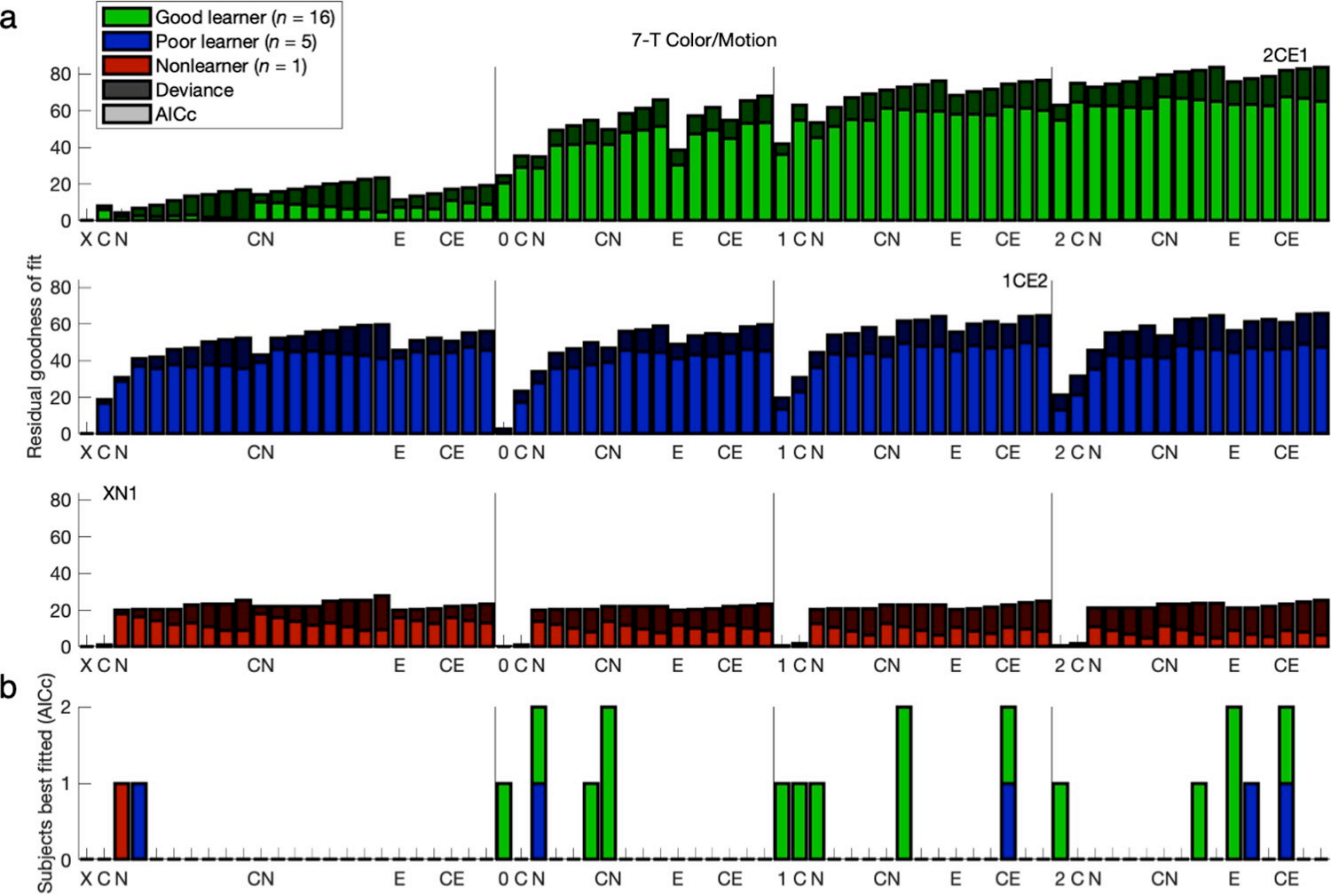
Model comparison: 7-T Color/Motion version. Compare to Fig 2. Results were replicated in the 7-T Color/Motion version of the experiment with a nearly identical experimental design.

Across all five participant groups from both data sets, the model comparison here established that the best-performing models featured not only GRL (for actual learners) but also constant bias and exponential hysteresis (FH-G: 2CE1, FH-P: 1CE3, FH-N: XCE2, CM-G: 2CE1, CM-P: 1CE2)—even after correcting for model complexity according to the Akaike information criterion with correction for finite sample size (AICc) [62,63] (**Figs 2A and 3A and Tables B-F in S1 Text**). Furthermore, at the individual level, 87% of participants exhibited significant effects of some kind of action-specific bias or hysteresis (FH: *n* = 41/47, CM: *n* = 19/22) (**Figs 2B and 3B and Figs Kd and Ld in S1 Text**).

With regard to correspondence between this bias-centric model comparison and the original learning-centric model comparison [12], individual Good learners were again always best fitted by a learning model (FH: *n* = 31/31, CM: *n* = 16/16), whereas Nonlearners were again always best fitted by a nonlearning model with nothing more than action bias or hysteresis (FH: *n* = 0/7, CM: *n* = 0/1). The boundary case of the Poor-learner group was mostly but not always in the direction of a learning model as opposed to a nonlearning model (FH: *n* = 6/9, CM: *n* = 4/5). Nevertheless, the original group assignments were retained here not only for consistency but also in consideration of the lack of a full factorial design with respect to GRL here (originally 11 models rather than 3).

As hypothesized for bias and hysteresis parameters, Nonlearners and even Poor learners showed greater gains in model performance than Good learners, but Good learners still benefited significantly as well. The Poor-learner and Nonlearner groups actually suggested greater explanatory power from additional hysteresis parameters (even over a third learning parameter): The best fits were from the 1CE3 and 1CE2 models for Poor learners and XCE2 for Nonlearners. Yet, in the interest of a universal model that is both parsimonious and straightforward, the 2CE1 model and the CE1 adjunct remained preferred overall for the present purposes because the Good-learner groups, which both favored 2CE1, are more reliable and more essential as evidence for a mixture of experts and nonexperts. These results and many others that follow confirmed that the original group assignments from the learning-centric model comparison remain applicable with reanalysis from this bias-centric perspective.

Although a simpler alternative nested within the 7-parameter 2CE1 model may provide a decent account for some individuals, this moderately complex model in itself provided the most parsimonious account for the greatest proportion of heterogeneous participants—and especially so among those who learned well. Conversely, the lesser overall performance of the 8- and 9-parameter models argues against an explanation reduced to mere overfitting. While omitting additional *n*-back degrees of freedom, the 2-parameter specification for exponential hysteresis was sufficiently flexible to best fit (post-correction) 64% of the heterogeneity across participants with nested models (FH: *n* = 28/47, CM: *n* = 16/22). As for the nonparametric equivalent in total degrees of freedom, substituting 2-back hysteresis (i.e., 2CN2) in lieu of the decay parameter would accommodate only 54% of this heterogeneity (FH: *n* = 24/47, CM: *n* = 13/22) in addition to providing a worse fit overall.

**Fig 4:**
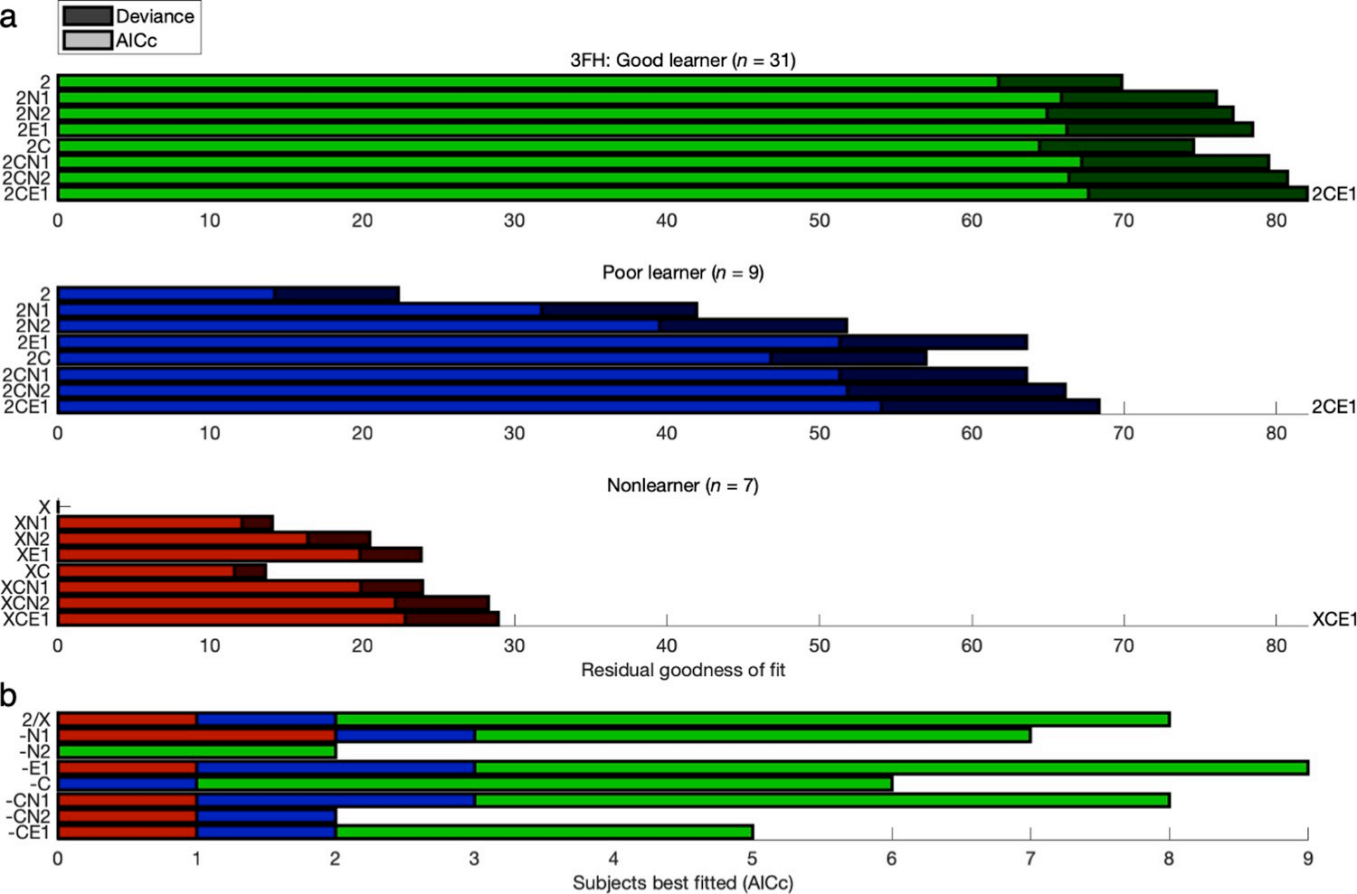
Reduced model comparison: 3-T Face/House version. Compare to Fig 2. The next round of comparisons focused on subsets of eight models building up to constant bias and exponential hysteresis (“-CE1”). The baseline models were 2-parameter GRL (“2”) for Good and Poor learners or a random policy (“X”) for Nonlearners. The evidence for best fit with the 2CE1 model is more visibly salient here (FH-G: 2CE1, FH-P: 2CE1, FH-N: XCE1; see Fig 5 for CM-G: 2CE1, CM-P: 2CN2).

**Fig 5:**
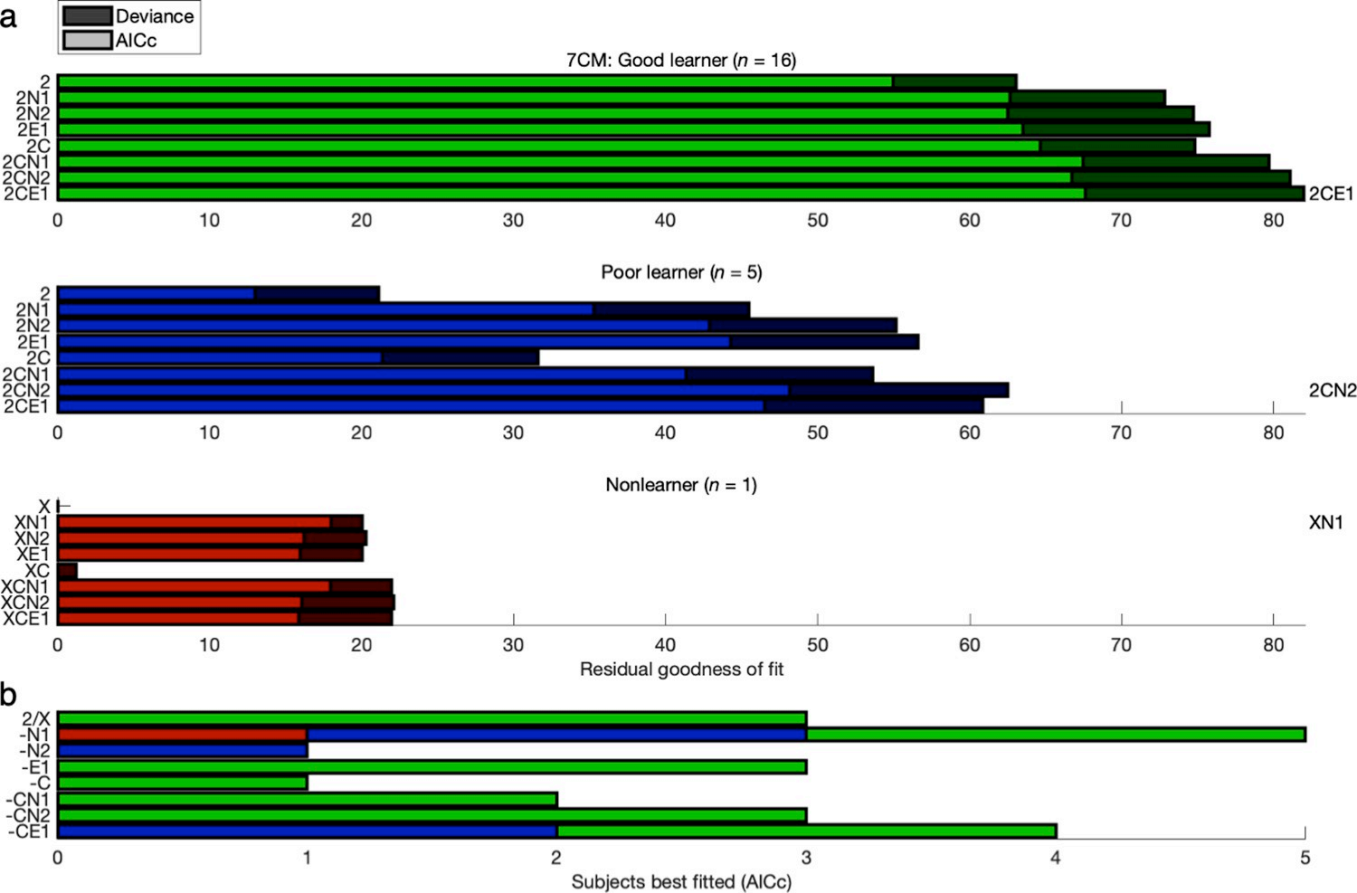
Reduced model comparison: 7-T Color/Motion version. Compare to Figs 3 and 4.

Having selected 2CE1 (and XCE1) with a large-scale comparison of 72 models, the most relevant subsets of eight models were rearranged for a follow-up comparison—namely, 2, 2N1, 2N2, 2E1, 2C, 2CN1, 2CN2, and 2CE1 (4 to 7 parameters) for the two learner groups and X, XN1, XN2, XE1, XC, XCN1, XCN2, and XCE1 (0 to 3 parameters) for the Nonlearner group (**Figs 4 and 5 and Figs Ka and La in S1 Text**). Between the edge cases of the no-bias model “2” and the full model 2CE1 were another six intermediate models—that is, four nested within 2CE1 featuring exponential hysteresis (2N1, 2E1, 2C, 2CN1) and two substituting 2-back hysteresis (2N2, 2CN2) with an equivalent number of degrees of freedom. The evidence for best fit with the 2CE1 model was more salient in this subset (FH-G: 2CE1, FH-P: 2CE1, FH-N: XCE1, CM-G: 2CE1, CM-P: 2CN2).

To again affirm the discriminability of the preferred 2CE1 model among both simpler and more complex alternatives ranging from 0 to 9 free parameters, simulated data sets were yoked to their respective empirical data sets but instead derived from individually fitted instantiations of this generative model. The simulated agent would receive input in silico according to what the respective human participant actually encountered in the session. When substituting simulated data generated by the instantiations of the 2CE1 model fitted to empirical data, the pattern of results could be replicated as expected (**Figs C, D, G, H, Kb/e, Lb/e, and M and Tables G-K in S1 Text**). Conversely, yoked simulations generated by the no-bias model “2” with only GRL—that is, a reduced model still biased toward reward maximization but unbiased with respect to action bias and hysteresis—shifted the fitting results to instead align with a learning-sans-bias model as expected (**Figs E, F, I, J, Kc/Kf, Lc/Lf, and M and Tables L-P in S1 Text**). In other words, the more complex model could be recovered from itself, and the simpler model could be recovered from itself, ruling out overfitting.

With the model comparison again (cf. [12]) pointing to the 7-parameter 2CE1 model, the individually fitted parameters of this model were verified and interpreted with reference to model-independent metrics for either action bias or learning performance (**Table 3**). The constant lateral bias *β*_*R*_ could be either leftward (*β*_*R*_ < 0) or rightward (*β*_*R*_ > 0), and its absolute value *|β*_*R*_*|* represents the weight of constant bias independent of direction—thereby resolving ambiguity between truly zero bias in the aggregate versus a distribution of substantial nonzero biases that are both positive and negative among individuals so as to cancel each other out. The initial magnitude of the exponential hysteresis bias *β*_*1*_ could accommodate both repetition (*β*_*1*_ > 0) and alternation (*β*_*1*_ < 0), where the unsigned weight *|β*_*1*_*|* represents either form in the 1-back hysteretic bias. Furthermore, the model’s overall level of bias—or at least 0-back and 1-back bias while overlooking the decaying remainder—could be quantified as *|β*_*R*_*|+|β*_*1*_*|* for a metric.

**Table 3:** Parameters of the 2CE1 model. Fitted parameters for the preferred 2CE1 model are listed for each participant group based on learning performance. To characterize the dimensions of distinct behavioral profiles for each participant, the signs of individual fits are categorized as “discriminative” (-1 ≤ *g*_*A*_ < 0) or “none” (*g*_*A*_ = 0) for action generalization; “discriminative” (-1 ≤ *g*_*S*_ < 0), “none” (*g*_*S*_ = 0), or “associative” (0 < *g*_*S*_ ≤ 1) for state generalization; “leftward” or (*β*_*R*_ < 0) “rightward” (*β*_*R*_ > 0) for constant bias; and “alternation” (*β*_*1*_ < 0) or “repetition” (*β*_*1*_ > 0) for hysteretic bias. Also listed are metrics for absolute constant bias *|β*_*R*_*|*, absolute hysteretic bias *|β*_*1*_*|*, and overall bias *|β*_*R*_*|+|β*_*1*_*|*, which is inversely related to the probability of a correct response (*p* < 0.05). The residual deviance *D*_*df*_ (with degrees of freedom in the subscript) corresponds to the 2CE1 model’s improvement in fit relative to either the XC model with only constant bias or the complete nonlearning model XCE1 adding exponential hysteresis. Standard deviations are listed in parentheses below corresponding means.

	3-T Face/House			7-T Color/Motion		
2D GRL + Con + Exp (2CE1)	Good learner	Poor learner	Non- learner	Good learner	Poor learner	Non- learner
*n*	31	9	7	16	5	1
Learning rate *α*	0.517 (0.242)	0.269 (0.339)	0.483 (0.345)	0.555 (0.345)	0.540 (0.353)	0.372
Action generalization *g*_*A*_	-0.355 (0.367)	-0.321 (0.376)	-0.787 (0.357)	-0.535 (0.393)	-0.551 (0.482)	-1.000
Discriminative : None	21 : 10	6 : 3	7 : 0	13 : 3	4 : 1	1 : 0
State generalization *g*_*S*_	-0.184 (0.344)	0.367 (0.535)	0.359 (0.887)	-0.239 (0.390)	0.257 (0.819)	1.000
Disc. : None : Associative	18 : 9 : 4	1 : 2 : 6	2 : 0 : 5	11 : 1 : 4	1 : 1 : 3	0 : 0 : 1
Softmax temperature *τ*	0.698 (0.464)	0.737 (0.565)	3.066 (0.724)	0.700 (0.343)	1.298 (0.782)	2.157
Rightward bias *β*_*R*_	0.113 (0.354)	0.160 (0.185)	0.391 (0.855)	0.167 (0.240)	0.245 (0.360)	-0.435
Leftward : Rightward	12 : 19	2 : 7	2 : 5	2 : 14	1 : 4	1 : 0
Repetition bias: Initial magnitude *β*_*1*_	-0.066 (0.235)	-0.133 (0.438)	-0.169 (1.034)	-0.130 (0.153)	-0.393 (0.949)	-1.278
Alternation : Repetition	21 : 10	4 : 5	4 : 3	13 : 3	3 : 2	1 : 0
Repetition bias: Inverse decay rate *λ*_*H*_	0.543 (0.371)	0.578 (0.404)	0.456 (0.421)	0.659 (0.318)	0.485 (0.403)	0.000
Constant bias *|β*_*R*_*|*	0.196 (0.314)	0.191 (0.149)	0.714 (0.561)	0.207 (0.204)	0.305 (0.298)	0.435
Hysteretic bias *|β*_*1*_*|*	0.171 (0.172)	0.228 (0.392)	0.868 (0.472)	0.152 (0.130)	0.717 (0.672)	1.278
Overall bias *|β*_*R*_*|+|β*_*1*_*|*	0.367 (0.449)	0.419 (0.396)	1.582 (0.969)	0.358 (0.276)	1.021 (0.584)	1.713
Constant (XC): Res. dev. *D*_*6*_	78.56	48.67	16.75	73.97	42.15	22.28
C + Exponential (XCE1): *D*_*4*_	70.55	28.94	1.46	64.82	10.31	1.51

To more rigorously test for effects of action bias and hysteresis even in the presence of competing effects of value-based learning, Nonlearners are excluded from many of the analyses that follow. Confirming parameter validity across both Good and Poor learners, the rightward bias *β*_*R*_ was correlated with the probability of performing the right-hand action (FH: *r* = 0.556, *t*_*38*_ = 4.13, *p* < 10^−4^; CM: *r* = 0.640, *t*_*19*_ = 3.63, *p* < 10^−3^). Likewise, the repetition bias *β*_*1*_ was correlated with the probability of repeating the previous action regardless of state (FH: *r* = 0.769, *t*_*38*_ = 7.40, *p* < 10^−8^; CM: *r* = 0.660, *t*_*19*_ = 3.83, *p* < 10^−3^). Given the exclusively right-handed participants in this study, the majority were expected to exhibit a net rightward bias (*β*_*R*_ > 0) like that even captured within the subgroups based on learning performance (FH-G: *M* = 0.113, *t*_*30*_ = 1.78, *p* = 0.043; FH-P: *M* = 0.160, *t*_*8*_ = 2.60, *p* = 0.016; FH-N: *M* = 0.391, *t*_*6*_ = 1.21, *p* = 0.136; CM-G: *M* = 0.167, *t*_*15*_ = 2.78, *p* = 0.007; CM-P: *M* = 0.245, *t*_*4*_ = 1.52, *p* = 0.102).

If action bias and hysteresis were omitted as is typically the case, estimation bias and other distortions of learning parameters would arise when forced to simultaneously fit these parallel phenomena that are otherwise unaccounted for. The necessity of the extra parameters could also be validated in silico with parameter recovery or lack thereof when simulating with or without bias parameters, respectively (**Fig N in S1 Text**). As compared with successfully recovering parameters of the full bias-and-hysteresis model 2CE1 (FH-G: α: *r* = 0.759, *p* < 10^−6^; g_A_: *r* = 0.731, *p* = 10^−6^; g_S_: *r* = 0.725, *p* = < 10^−5^; τ: *r* = 0.668, *p* < 10^−4^; β_R_: *r* = 0.624, *p* = 10^−4^; β_1_: *r* = 0.876, *p* < 10^−10^; λ_H_: *r* = 0.463, *p* = 0.004; FH-P: α: *r* = 0.841, *p* = 0.002; g_A_: *r* = 0.853, *p* = 0.002; g_S_: *r* = 0.819, *p* = 0.003; τ: *r* = 0.306, *p* = 0.212; β_R_: *r* = 0.824, *p* = 0.003; β_1_: *r* = 0.725, *p* = 0.014; λ_H_: *r* = 0.666, *p* = 0.025; CM-G: α: *r* = 0.638, *p* = 0.004; g_A_: *r* = 0.472, *p* = 0.033; g_S_: *r* = 0.621, *p* = 0.005; τ: *r* = 0.697, *p* = 10^−3^; β_R_: *r* = 0.717, *p* < 10^−3^; β_1_: *r* = 0.588, *p* = 0.008; λ_H_: *r* = 0.448, *p* = 0.041; CM-P: α: *r* = 0.786, *p* = 0.058; g_A_: *r* = 0.866, *p* = 0.029; g_S_: *r* = 0.891, *p* = 0.021; τ: *r* = 0.885, *p* = 0.023; β_R_: *r* = 0.974, *p* = 0.003; β_1_: *r* = 0.856, *p* = 0.032; λ_H_: *r* = 0.996, *p* < 10^−3^), recovery of the learning parameters from 2CE1 with the no-bias model “2” was generally less robust for all learners and especially insufficient—even failing to recover—for the Poor-learner group more characterized by action biases that outweigh and obscure confounded learning processes (FH-G: α: *r* = 0.291, *p* = 0.056; g_A_: *r* = 0.535, *p* = 10^−3^; g_S_: *r* = 0.744, *p* < 10^−6^; τ: *r* = 0.658, *p* < 10^−4^; FH-P: α: *r* = 0.430, *p* = 0.124; g_A_: *r* = 0.172, *p* = 0.329; g_S_: *r* = 0.418, *p* = 0.131; τ: *r* = 0.374, *p* = 0.161; CM-G: α: *r* = 0.683, *p* = 0.002; g_A_: *r* = 0.592, *p* = 0.008; g_S_: *r* = 0.604, *p* = 0.007; τ: *r* = 0.690, *p* = 0.002; CM-P: α: *r* = 0.716, *p* = 0.087; g_A_: *r* = 0.631, *p* = 0.127; g_S_: *r* = 0.995, *p* < 10^−3^; τ: *r* = 0.796, *p* = 0.054).

The deficiencies of a model limited to only learning are especially noteworthy in this contrived environment with experimental controls regulating the reward schedule such that spurious confounds between effects of learning and effects of bias and hysteresis have been mitigated by design. The proof of concept in this extreme case with unnatural controls suggests an even more pressing need for this framework for applications in less controlled laboratory settings as well as natural settings in the real world. Elsewhere without such experimental control via deliberate counterbalancing that would otherwise impose symmetric structure in the environment as well as individual trajectories within it, there would be even greater susceptibility to parameter distortion if bias parameters were omitted.

### Action bias and hysteresis versus learning performance

In keeping with the previous point about idiosyncratic environments, the statistics of a given task environment must be considered to set reference points for quantifying and interpreting truly action-specific components of variance. While triple dissociation of bias, hysteresis, and learning is generally nontrivial for a short sequence of active states, this challenge can be exacerbated even more so by class imbalance depending on the temporal statistics of states, actions, and rewards. In arriving at a fully interpretable quantitative model amenable to individual differences, the challenge was first met here by a hierarchically counterbalanced experimental design that was tightly controlled within and across sessions.

Regarding the constant lateral bias, available rewards were thus evenly distributed between left-hand and right-hand actions all throughout the experiment. Hence an omniscient optimal agent with perfect 100% accuracy would be guaranteed to produce an even 50% probability of a left- or right-hand action. This was not the case for hysteresis, however.

In contrast, that same agent would produce an uneven 66.7% probability of action alternation as a byproduct of choosing the optimal actions here. This incidental asymmetry can superficially mimic an internal alternation bias while a learner actually responds to the external structured sequence of four randomly rotating states. (States were never repeated in consecutive trials, and of the three remaining states, only one from the complementary category would reward the action just performed in a given state for the block—resulting in two-thirds or 66.7% alternation.) Note that a naïve policy with a 100% probability of alternation irrespective of state would nonetheless produce chance accuracy at 50% by design. Such ambiguity for a raw, model-independent measure again underscores the need for comprehensive computational modeling that accounts for multiple implicit effects simultaneously.

To the extent that the forces of bias and learning compete with each other to drive behavior, an inverse relation was expected between learning performance and the weight of action bias and hysteresis. Again omitting Nonlearners, overall bias *|β*_*R*_*|+|β*_*1*_*|* in actual learners was inversely correlated with accuracy as the probability of choosing the correct action (FH: *r* = -0.290, *t*_*38*_ = 1.87, *p* = 0.035, *r*_*S*_ = -0.374, *p* = 0.009 for monotonicity; CM: *r* = -0.472, *t*_*19*_ = 2.33, *p* = 0.015, *r*_*S*_ = -0.605, *p* = 0.002 for monotonicity). This inverse relation between modeled bias and objective performance was monotonic across not only all learners but also the alternation-bias group specifically (FH: *r* = -0.383, *t*_*23*_ = 1.99, *p* = 0.029, *r*_*S*_ = -0.475, *p* = 0.009 for monotonicity; CM: *r* = -0.453, *t*_*14*_ = 1.90, *p* = 0.039, *r*_*S*_ = -0.618, *p* = 0.006 for monotonicity), demonstrating that bias as extracted with modeling was not confounded with alternation that may incidentally result from pursuing reward. (See next section for more detail about the alternation-bias group.)

To complement the initial quantitative model comparison for overall goodness of fit, a series of posterior predictive checks followed for evidence of bias and hysteresis with qualitative falsification of the null hypotheses in nested models [26–28]. The same technique had been used previously to falsify basic RL against GRL [12]. Each check entailed juxtaposition of empirical behavior and the behavior simulated by GRL models that, while holding a fixed assumption of two new learning parameters for generalization, are incrementally tested with up to three more action-bias parameters.

First separating groups on the basis of learning performance, a binary model comparison could illustrate some fundamental limitations of the pure GRL model “2” with no bias as opposed to the final 2CE1 model with three parameters for constant bias and exponential hysteresis. (The intermediate models between these 4- and 7-parameter end points are investigated in greater depth later.) Posterior predictive checks for these two models were tested against empirical results for not only the probability of a correct (versus incorrect) action—as is standard for a learning paradigm—but also the probability of a right-hand (versus left-hand) action and the probability of a repeated (versus alternated) action independent of state.

**Fig 6:**
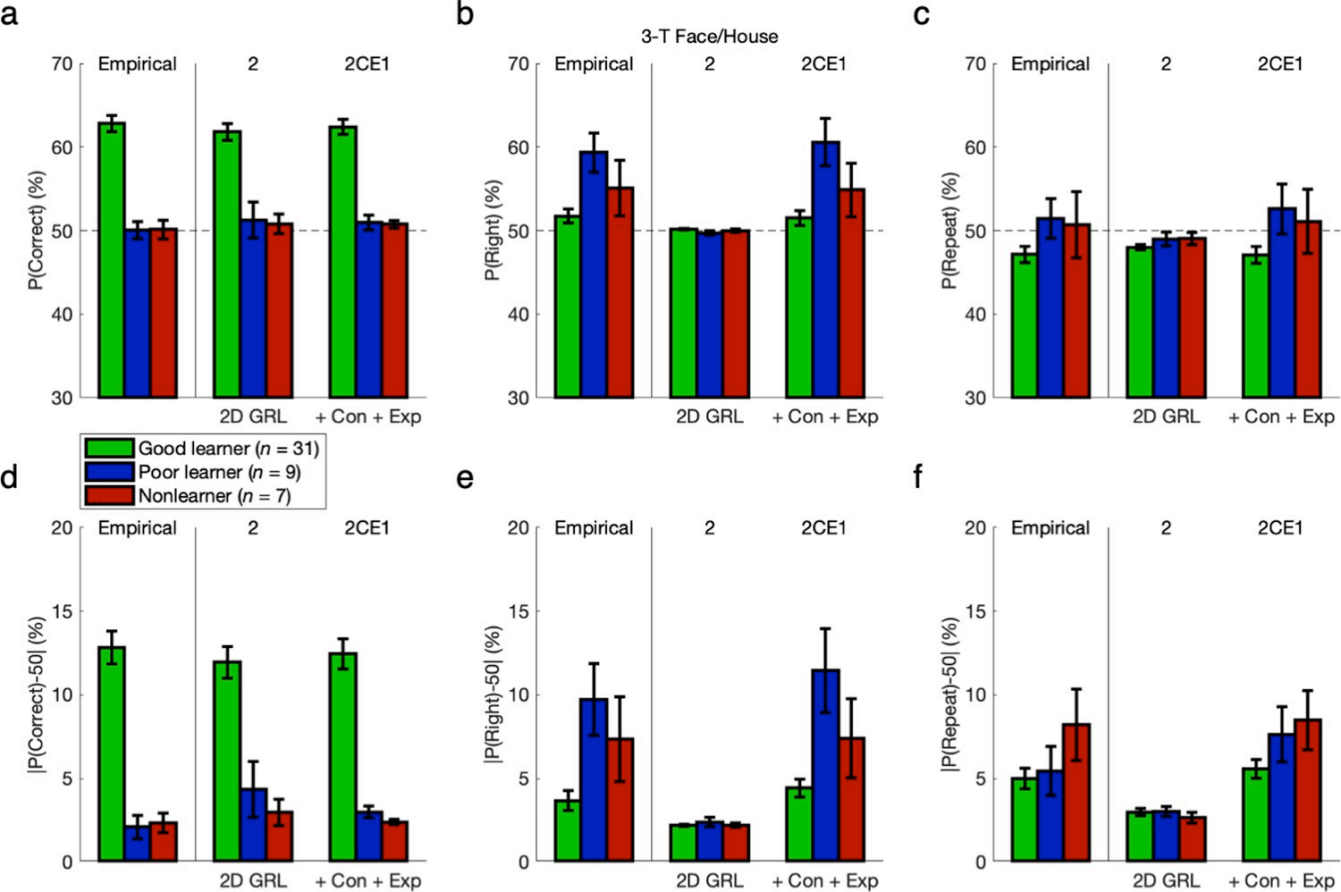
Action bias and hysteresis versus learning performance: 3-T Face/House version. To compare the pure GRL model (“2”) with the final 2CE1 model adding three parameters for constant bias and exponential hysteresis, simulated data sets from each model were yoked to their respective empirical data sets. Posterior predictive checks were tested for the probability of a correct action, the probability of a right-hand action, or the probability of a repeated action independent of state. **(a)** If only examining accuracy in terms of correct choices for maximizing reward, the shortcomings of the reduced model without bias are not so obviously apparent at first. **(b)** Upon considering action bias, these right-handed individuals mostly had a tendency to select the right-hand action (*p* < 0.05). Whereas the 2CE1 model could account for this effect with a constant lateral bias (*p* < 0.05), the reduced model could not (*p* > 0.05). **(c)** Regarding the probability of repetition versus alternation, note that 100% accuracy would produce 66.7% alternation for the present experimental design, but 100% alternation would still produce 50% accuracy. The Good-learner group exhibited a tendency to alternate in the aggregate as expected (*p* < 0.05), whereas the Poor-learner and Nonlearner groups did not (*p* > 0.05). Only the 2CE1 model featuring exponential hysteresis could match this pattern with quantitative precision. **(d-f)** Independent of direction, absolute differences from the chance level of 50% reveal the full extent of the action-specific components of variance, which are as substantial as the effects of reward typically emphasized in active learning. For fitting the probability of a right-hand action or a repeated action, a margin of roughly 2% for pure GRL was insubstantial in comparison. Error bars indicate standard errors of the means.

**Fig 7:**
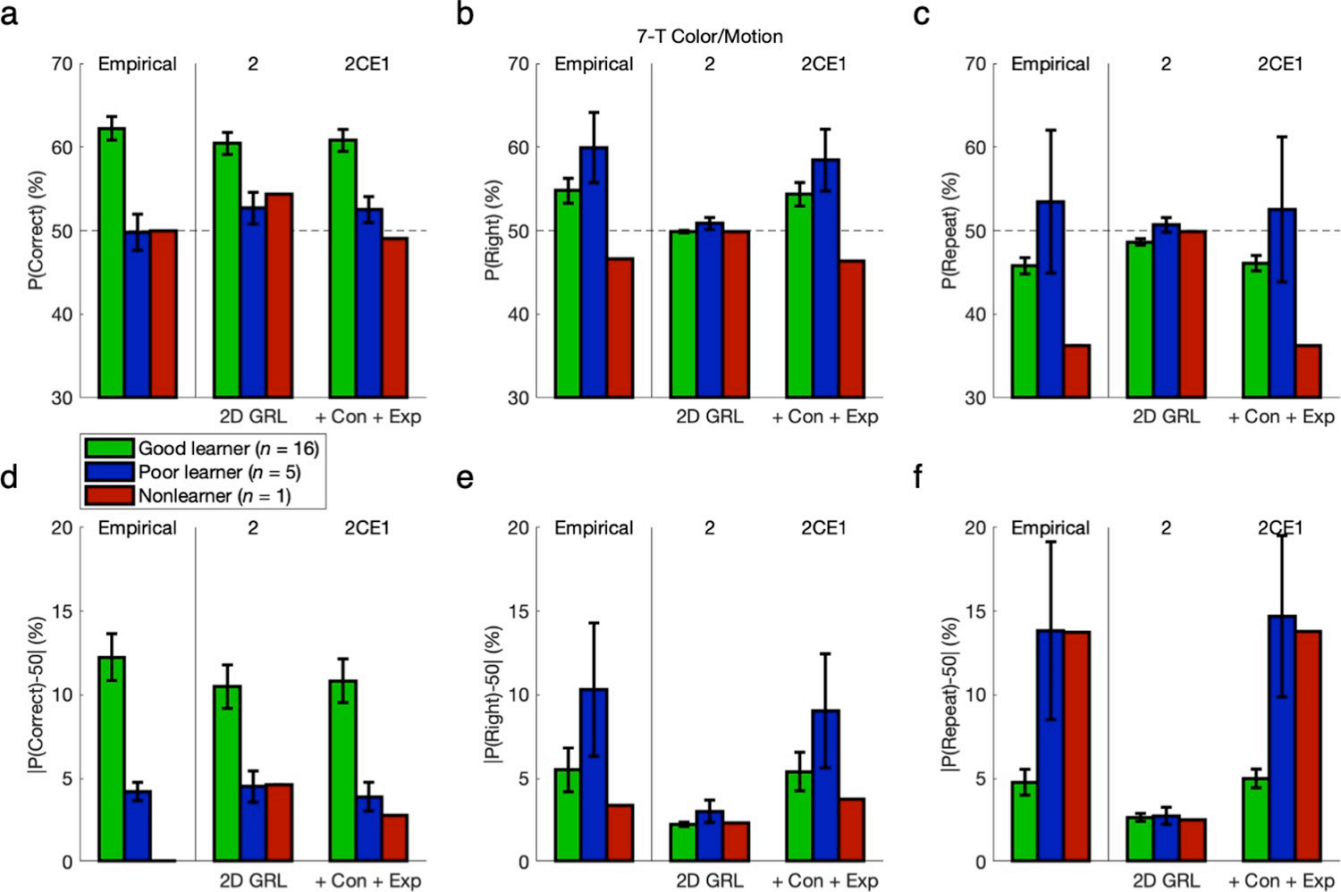
Action bias and hysteresis versus learning performance: 7-T Color/Motion version. Compare to Fig 6. Results were replicated in the 7-T Color/Motion version of the experiment.

From a naïve perspective it would appear that, by qualitatively capturing the probability of a correct choice across levels of learning performance (FH-G: *M* = 12.8%, *t*_*30*_ = 13.13, *p* < 10^−13^; FH-P: *M* = 0.1%, *p* > 0.05; FH-N: *M* = 0.1%, *p* > 0.05; CM-G: *M* = 12.3%, *t*_*15*_ = 8.75, *p* = 10^−7^; CM-P: *M* = -0.2%, *p* > 0.05) in silico as well (FH-G: *p* < 0.05; FH-P: *p* > 0.05; FH-N: *p* > 0.05; CM-G: *p* < 0.05; CM-P: *p* > 0.05) (**Figs 6A/6D and 7A/7D and Fig Oa/d in S1 Text**), the 4-parameter GRL model “2” with no bias seemingly accounts for human behavior comparably to the 7-parameter 2CE1 model expanded with action bias and hysteresis. However, the shortcomings of a purely learning-based account can be revealed even in 0-back and 1-back action-specific effects. Remarkably, these action-specific effects (**Figs 6E–6F and 7E–7F**) are quite substantial in effect size as compared with the value-based effects (**Figs 6D and 7D**) typically and most intuitively emphasized in a paradigm for active learning.

Across these right-handed participants, all five groups in the aggregate performed the right-hand action more often (FH-G: *M* = 1.8%, *t*_*30*_ = 2.11, *p* = 0.022; FH-P: *M* = 9.3%, *t*_*8*_ = 3.99, *p* = 0.002; FH-N: *M* = 5.1, *t*_*6*_ = 1.54, *p* = 0.088; CM-G: *M* = 4.8%, *t*_*15*_ = 3.21, *p* = 0.003; CM-P: *M* = 9.9%, *t*_*4*_ = 2.36, *p* = 0.039) (**Figs 6B/6E and 7B/7E and Fig Ob/Oe in S1 Text**), and greater or marginally greater rightward bias was observed in Poor learners and Nonlearners relative to Good learners (FH-PG: *M* = 7.6%, *t*_*38*_ = 3.80, *p* < 10^−3^; FH-NG: *M* = 3.3%, *t*_*36*_ = 1.43, *p* = 0.081; CM-PG: *M* = 5.2%, *t*_*19*_ = 1.47, *p* = 0.079). Hence this measure of absolute lateral bias *|P(Right)-50%|* was also greater in Poor learners and Nonlearners (FH-PG: *M* = 6.0%, *t*_*38*_ = 3.81, *p* < 10^−3^; FH-NG: *M* = 3.7%, *t*_*36*_ = 2.14, *p* = 0.020; CM-PG: *M* = 4.8%, *t*_*19*_ = 1.51, *p* = 0.074), which likewise held true when correlating across the continuous measure of accuracy rather than discrete participant groups (FH: *r* = -0.544, *t*_*38*_ = 4.00, *p* = 10^−4^; CM: *r* = -0.540, *t*_*19*_ = 2.80, *p* = 0.006). Whereas the full 2CE1 model could replicate all of these effects (*p* < 0.05), the reduced GRL model could not (*p* > 0.05). As a reflection of individual-specific class imbalance or overfitting in the absence of constant bias, a roughly 2% margin was apparent in the absolute difference between the reduced model’s right-hand probability and the chance level of 50% (**Figs 6E and 7E**). Yet this margin was insubstantial in comparison to the true effect sizes of constant bias that were quantitatively matched by only the full model.

Note again that 100% accuracy in this contrived environment would produce 66.7% alternation because of rotating states, but 100% alternation would produce 50% accuracy. The interpretation of this raw measure is thus confounded between effects of reward and hysteresis, but in keeping with the statistics of the environment, the Good-learner groups did exhibit a tendency to alternate in the aggregate while the Poor-learner and Nonlearner groups did not (FH-G: *M* = -2.9%, *t*_*30*_ = 2.94, *p* = 0.003; FH-P: *M* = 1.5%, *p* > 0.05; FH-N: *M* = 0.8%, *p* > 0.05; CM-G: *M* = -4.2%, *t*_*15*_ = 4.34, *p* < 10^−3^; CM-P: *M* = 3.5%, *p* > 0.05) (**Figs 6C/6F and 7C/7F and Fig Oc/f in S1 Text**). In contrast, the absolute repetition-or-alternation frequency *|P(Repeat)-50%|* was significantly greater than chance for all subgroups (FH-G: *M* = 5.0%, *t*_*30*_ = 8.11, *p* < 10^−8^; FH-P: *M* = 5.5%, *t*_*8*_ = 3.73, *p* = 0.003; FH-N: *M* = 8.2%, *t*_*6*_ = 3.84, *p* = 0.004; CM-G: *M* = 4.8%, *t*_*15*_ = 6.15, *p* < 10^−5^; CM-P: *M* = 13.8%, *t*_*4*_ = 2.60, *p* = 0.030). Relative to Good learners, Nonlearners exhibited even greater deviation from chance with repetition or alternation (*M* = 3.2%, *t*_*36*_ = 1.97, *p* = 0.028), as did the Poor learners of at least the second data set (*M* = 9.1%, *t*_*19*_ = 2.89, *p* = 0.005). The latter trend held true for the second data set with marginal significance for the continuous measure of accuracy as well (*r* = -0.312, *t*_*19*_ = 1.43, *p* = 0.084). Only the 7-parameter model could match net 1-back effects with quantitative precision (FH-G: *p* < 0.05; FH-P: *p* > 0.05; FH-N: *p* > 0.05; CM-G: *p* < 0.05; CM-P: *p* > 0.05), and qualitative falsification of the pure GRL model for such hysteretic effects was to be found in follow-up analyses disambiguating effects of reward and hysteresis. Owing to this disambiguation, the model-based results that follow are more reliable than these model-independent measures for inference about actual hysteresis per se.

### Different forms of action bias and hysteresis

The 2CE1 model should accommodate the idiosyncrasies of individual participants with respect to not only GRL, which has already been demonstrated [12], but also action bias and hysteresis. Based on parameter fits, Good and Poor learners were combined and then reclassified according to the directionality of either constant bias or hysteretic bias—that is, leftward (*β*_*R*_ < 0) versus rightward (*β*_*R*_ > 0) or alternation (*β*_*1*_ < 0) versus repetition (*β*_*1*_ > 0). Nonlearners were again omitted for more rigorous testing of biases in the presence of actual learning. Each posterior predictive check was extended to the eight models previously highlighted in the reduced model comparison—that is, incrementally building up from the no-bias model “2” with only GRL (4 parameters) to the full 2CE1 model (7 parameters). Necessity could thus be verified for every single parameter of the 2CE1 model.

**Fig 8:**
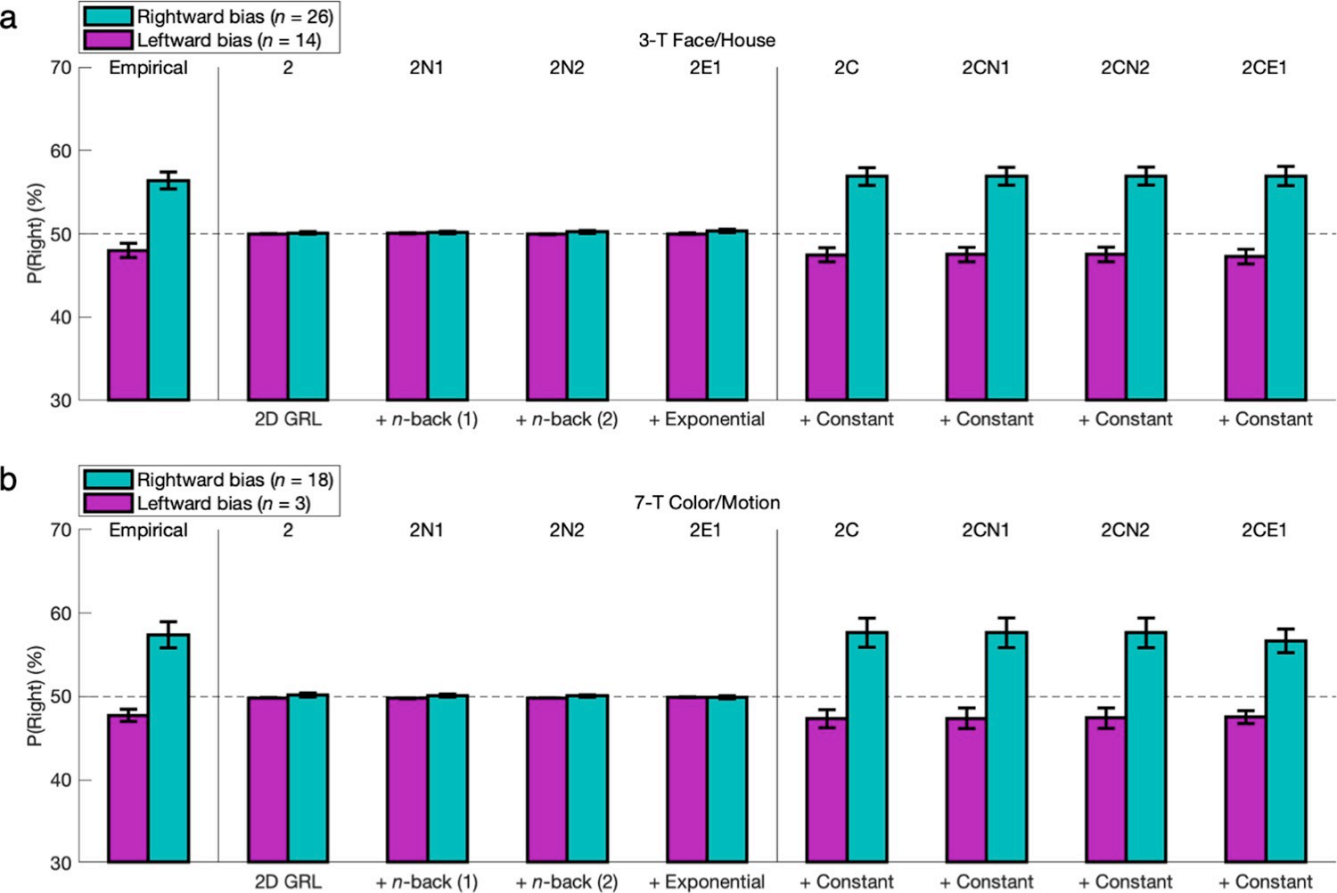
Constant bias. **(a)** Based on individual fits of the 2CE1 model, Good and Poor learners were combined and then reclassified according to whether the constant lateral bias was a leftward bias (*β*_*R*_ < 0) (magenta bars) or a rightward bias (*β*_*R*_ > 0) (cyan bars). The model comparison extended this posterior predictive check and others to another six intermediate models—four models nested within the 2CE1 model featuring exponential hysteresis (2N1, 2E1, 2C, 2CN1) and two models substituting 2-back hysteresis (2N2, 2CN2) but matched for degrees of freedom. For the probabilities of left or right actions, some of these right-handed people actually exhibited a contrary leftward bias; those who did exhibited a smaller absolute magnitude of bias than that of the rightward-bias group (*p* < 0.05). The models with a parameter for constant bias (2C through 2CE1) could replicate these effects (*p* < 0.05), falsifying the models that could not at all for lack of this parameter (*p* > 0.05). **(b)** Results were replicated in the 7-T Color/Motion version of the experiment.

Among these right-handed learners, 28% exhibited a contrary leftward bias (FH: *n* = 14/40; CM: *n* = 3/21). Those with leftward bias (FH: *M* = -2.0%, *t*_*13*_ = 2.29, *p* = 0.020; CM: *M* = -2.3%, *t*_*2*_ = 3.12, *p* = 0.045) exhibited a smaller (or marginally smaller) absolute magnitude of bias (FH: *M* = 4.2%, *t*_*38*_ = 2.84, *p* = 0.004; CM: *M* = 5.1%, *t*_*19*_ = 1.31, *p* = 0.103) relative to the rightward-bias group (FH: *M* = 6.4%, *t*_*25*_ = 6.30, *p* < 10^−6^; CM: *M* = 7.4%, *t*_*17*_ = 4.73, *p* < 10^−4^) (**Fig 8**), but the existence of so many leftward biases among right-handed individuals is noteworthy. The models with a parameter for constant bias (2C through 2CE1) could replicate these effects (*p* < 0.05), whereas those without the parameter could not at all (*p* > 0.05). These findings falsify the naïve hypothesis that handedness might determine the direction of constant bias invariably. The unpredictable distribution of an effect as simple as laterality stands among the evidence that, in general, individual differences must be modeled without a-priori distributional assumptions—whether about a random sample of individuals or about the population from which they are drawn (see Discussion).

Bear in mind that optimal behavior results in more frequent alternation of actions in this particular setting. Conversely, naïve alternation does not result in above-chance performance for the aforementioned reasons. Despite the latter fact, behavior was hypothesized to be predisposed to alternation that is independent of states and outcomes after an agent has been alternating actions at the appropriate times due to learning that is dependent on states and outcomes. This hypothesis might initially appear at odds with the typical narrative in the RL literature emphasizing perseveration as naïve action repetition, but here, that would only represent first-order perseveration at the level of actions. At the level of policies, second-order perseveration suggests that a learner in such an environment perseverates from an expert reward-seeking policy of optimal alternation when appropriate to a nonexpert default policy of perseverative alternation whenever.

**Fig 9:**
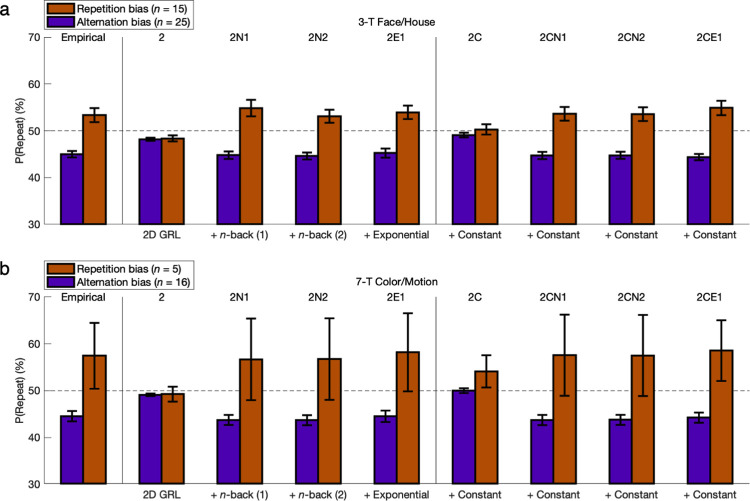
Hysteresis represented by the previous trial. The learners were next reclassified according to whether the hysteretic bias was an alternation bias (*β*_*1*_ < 0) (violet bars) or a repetition bias (*β*_*1*_ > 0) (orange bars). With some adhering to a more typical profile of first-order perseveration, the repetition-bias group did retain a substantial effect on the probability of repeating an action independent of state (*p* < 0.05). However, in keeping with second-order perseveration, the alternation-bias group actually outnumbered and outweighed in effect size the repetition-bias group (*p* < 0.05). That is, extra alternation could follow from the design feature whereby optimal behavior would more frequently result in alternating actions. In contrast to optimal alternation when appropriate for a given state, this perseverative alternation was action-specific so as to not actually improve reward-maximizing accuracy for the alternation-bias group (*p* > 0.05). The models with at least one parameter for hysteretic bias could replicate these 1-back effects (*p* < 0.05). Although the 2C model with constant bias could partially mimic action repetition with a nonsignificant trend, the models without any hysteresis parameters (2 and 2C) could not properly match the empirical 1-back effect (*p* > 0.05).

In keeping with this hypothesis, the alternation-bias group (FH: *n* = 25/40; CM: *n* = 16/21) was expected to outnumber the repetition-bias group (FH: *n* = 15/40; CM: *n* = 5/21) as well as exhibit an effect on the raw probability of alternation (FH: *M* = -5.0%, *t*_*24*_ = 7.32, *p* < 10^−7^; CM: *M* = -5.4%, *t*_*15*_ = 4.93, *p* < 10^−4^) (**Fig 9**). Yet reward-maximizing accuracy was not significantly higher for the alternation-bias group than for the repetition-bias group (FH: *M* = 3.2%, *p* > 0.05; CM: *M* = 2.2%, *p* > 0.05), confirming the action-specific nature of this bias as a nonexpert heuristic. The arrow of causality for the hypothesis of second-order perseveration primarily points from optimal alternation to perseverative alternation rather than vice versa. These results lend themselves to an analogy with the previously described cohort that was left-biased despite being right-handed, whereas there was still also a sizable repetition-bias group in which some learners instead adhered to a more intrinsic first-order perseveration effect like what has typically been reported in the literature. That is, this learning cohort could sometimes alternate to exploit actions with high estimated reward when appropriate but still perseverated so as to repeat actions according to a more robust default repetition bias (FH: *M* = 3.3%, *t*_*14*_ = 2.24, *p* = 0.021; CM: *M* = 7.4%, *t*_*4*_ = 1.06, *p* = 0.175; nonsignificant, but versus alternation-bias group: *M* = 12.9%, *t*_*19*_ = 3.06, *p* = 0.003). Whereas the models with at least one parameter for hysteretic bias (including the simplest 2N1 model) could replicate these 1-back effects (*p* < 0.05), the models with no such parameter could not (*p* > 0.05).

Notably, the 2C model with constant bias but no hysteresis could partially mimic the repetition effect observed in the repetition-bias group (with a trending but nonsignificant result, *p* > 0.05). That is, a true action-repetition effect could be overfitted to some extent by instead representing only imbalanced base rates for actions. Although this reduced constant-only model fails to match the empirical repetition result quantitatively, there is cause for alarm in the qualitative trend that spuriously arises in both data sets. As discussed previously, the present environment represents a distinct active-learning paradigm in which such class imbalance is actually minimized—unlike most other environments with greater confounding in distributions for classes such as those of the actions per se or repetitions versus alternations. In general, omission of repetition bias may inflate estimates of constant bias with limited data if there is insufficient opportunity for repetition to be demonstrated across multiple actions. Likewise, omission of constant bias may inflate estimates of a confounded repetition effect. Conversely, omission of alternation bias may deflate estimates of constant bias because this alternation counteracts the incidental repetition of an action with a greater base rate. The different forms of bias and hysteresis all need to be accounted for comprehensively.

### Psychometric modeling of the mixture policy

**Fig 10:**
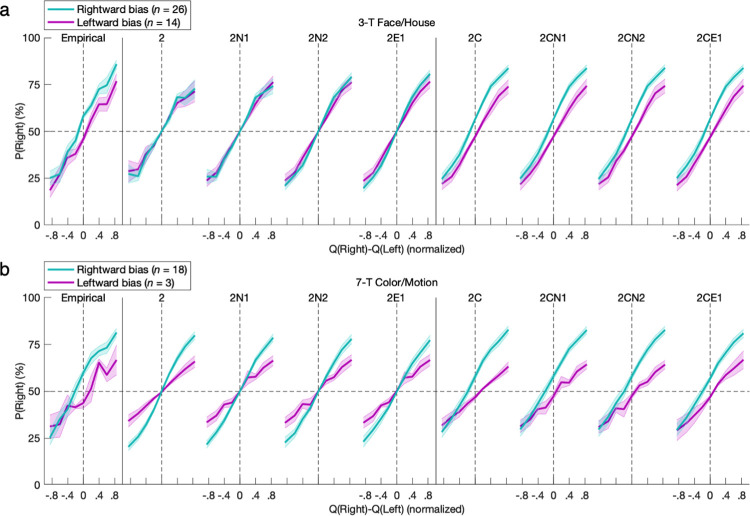
Psychometric modeling of constant bias. The probability of an action increased with the difference between action values *Q*_*t*_*(s*_*t*_,*a)* derived from the GRL component of the 2CE1 model as fitted to empirical behavior (*p* < 0.05). Constant bias was derived from a logistic model in the appropriate directions for both the leftward-bias and rightward-bias groups (*p* < 0.05). The models featuring constant bias could replicate these effects with quantitative precision as well (*p* < 0.05), whereas models without the parameter could not (*p* > 0.05). The nine plots per row each have an identical x-axis despite omission of tick labels from every other plot for readability. Error bars indicate standard errors of the means.

**Fig 11:**
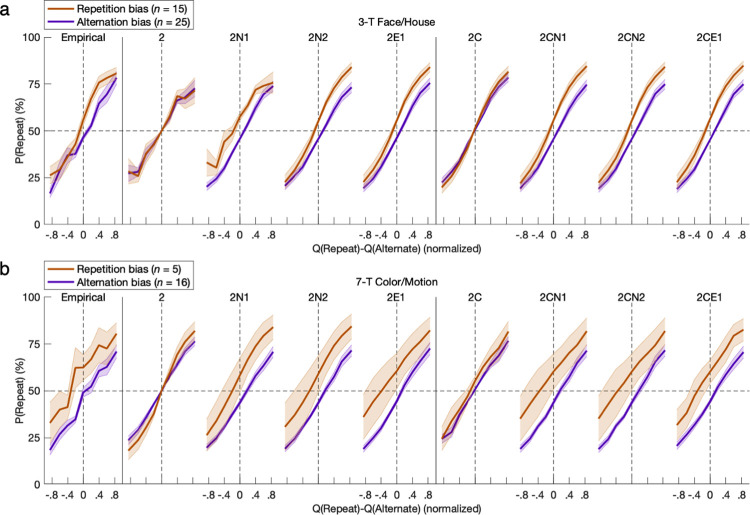
Psychometric modeling of hysteresis represented by the previous trial. For instead the probabilities of alternated or repeated actions, hysteretic bias was likewise derived from a GRL-based logistic model in the appropriate directions for both the alternation-bias and repetition-bias groups (*p* < 0.05). The models featuring at least one parameter for hysteretic bias could replicate these 1-back effects with comparable psychometric functions (*p* < 0.05), and while models without the parameter could not (*p* > 0.05), the 2C model could again deceptively mimic repetition with a nonsignificant trend.

More quantitatively precise modeling of psychometric functions followed to examine the interface of value-based learning, action-specific effects, and the softmax function determining the mixture policy for action selection. The breadth of this mixture of experts and nonexperts integrated modular elements of basic RL, generalized RL, constant bias, hysteretic bias, and stochasticity from exploration as well as noise. As expected across all subgroups of learners, the probability of an action increased with the difference between the state-dependent action values *Q*_*t*_*(s*_*t*_,*a)* learned by the GRL component of the 2CE1 model as fitted to empirical behavior (FH-L: *β* = 1.544, *t*_*13*_ = 6.38, *p* = 10^−5^; FH-R: *β* = 2.084, *t*_*25*_ = 6.74, *p* < 10^−6^; FH-A: *β* = 1.682, *t*_*24*_ = 9.60, *p* < 10^−9^; FH-P: *β* = 2.316, *t*_*14*_ = 4.61, *p* < 10^−3^; CM-L: *β* = 0.938, *t*_*2*_ = 2.67, *p* = 0.058; CM-R: *β* = 1.494, *t*_*17*_ = 7.20, *p* < 10^−6^; CM-A: *β* = 1.443, *t*_*15*_ = 7.20, *p* < 10^−5^; CM-P: *β* = 1.76, *t*_*4*_ = 2.97, *p* = 0.021) (**Figs 10 and 11**).

In determining the probability of left-hand versus right-hand actions, constant bias was derived from the logistic model in the appropriate directions for both the leftward-bias (FH: *β* = -0.113, *t*_*13*_ = 2.93, *p* = 0.006; CM: *β* = -0.103, *t*_*2*_ = 2.97, *p* = 0.049) and rightward-bias (FH: *β* = 0.265, *t*_*25*_ = 6.98, *p* = 10^−7^; CM: *β* = 0.302, *t*_*17*_ = 5.08, *p* < 10^−4^) groups (**Fig 10**). The models featuring constant bias could replicate these effects with comparable psychometric functions (*p* < 0.05), whereas models without the parameter could not (*p* > 0.05).

For instead the probability of repeated versus alternated actions independent of state, hysteretic bias was derived from the logistic model in the appropriate directions for both the alternation-bias (FH: *β* = -0.178, *t*_*24*_ = 5.21, *p* = 10^−5^; CM: *β* = -0.220, *t*_*15*_ = 5.31, *p* < 10^−4^) and repetition-bias (FH: *β* = 0.218, *t*_*14*_ = 4.79, *p* = 10^−4^; CM: *β* = 0.462, *t*_*4*_ = 1.35, *p* = 0.124; nonsignificant, but versus alternation-bias group: *M* = 0.682, *t*_*19*_ = 3.51, *p* = 0.001) groups (**Fig 11**). The models featuring at least one parameter for hysteretic bias could replicate these 1-back effects with comparable psychometric functions (*p* < 0.05), and while models without the parameter could not (*p* > 0.05), the solitary constant bias of the 2C model does deceptively mimic repetition with a nonsignificant trend.

### Dynamics of action hysteresis

The hysteresis trace of the 2CE1 model extends its temporal horizon beyond the 1-back effects examined thus far. For the preceding posterior predictive checks, the extra parameter for exponential decay could not explicitly show the full extent of its impact—showing instead only subtle quantitative improvement. If this costly free parameter were to be justified, its improvement for the model would need to also be qualitative and substantial. Considering that the 2CE1 model has already been shown to outperform both simpler and more complex implementations of hysteresis overall, the assumption of two parameters for exponential hysteresis must provide a superior parsimonious fit for effects of action history ranging from 2-back onward with an indefinite horizon. Moreover, 2-parameter exponential hysteresis outperformed *n*-back models for not only *n* = 1 but also *n* = 2 (2CN1 and 2CN2), establishing that it must not be only the 2-back effects but rather also 3-back and beyond that have significant weight beyond 1-back. Accordingly, hysteretic effects were explored directly up to eight trials back.

**Fig 12:**
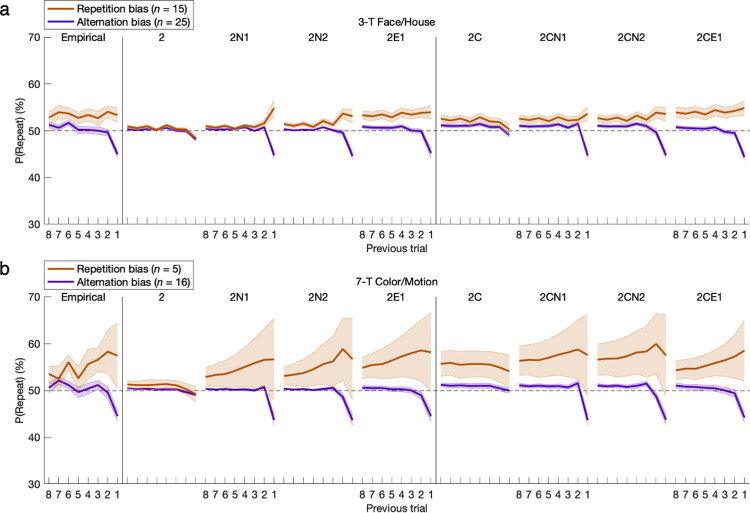
Hysteresis represented across multiple trials. Here the scope of hysteresis was extended to previous actions up to eight trials back. For the repetition-bias group, this probability of repeating a previous action remained elevated above chance prior to 1-back (*p* < 0.05). For the alternation-bias group, this probability instead returned from a 1-back alternation effect (*p* < 0.05) to chance prior to 1-back as it increases backward (*p* > 0.05). Only the models with exponential hysteresis could properly match the shapes of the action-history curves, and the addition of constant bias made the correspondence even more precise. With regard to mimicry, an upward shift in the curve from constant bias in the 2C model superficially resembles the autocorrelational signature of repetition across multiple trials with exponential hysteresis. The nine plots per row each have an identical x-axis despite omission of tick labels from every other plot for readability. Error bars indicate standard errors of the means.

The probability of a repeated action was now conditioned on each respective action from the eight most recent trials (**Fig 12**; see **Fig P in S1 Text** for distributions of runs of consecutive repeats). As expected for the repetition-bias group, this probability of repeating a previous action (FH: *M* = 3.3%, *t*_*14*_ = 2.24, *p* = 0.021; CM: *M* = 7.4%, *t*_*4*_ = 1.06, *p* = 0.175; nonsignificant, but versus alternation-bias group: *M* = 12.9%, *t*_*19*_ = 3.06, *p* = 0.003) was elevated above chance prior to 1-back as well (FH: *M* = 4.1%, *t*_*14*_ = 3.39, *p* = 0.002; CM: *M* = 8.3%, *t*_*4*_ = 1.83, *p* = 0.070 with marginal significance) and remained elevated. Conversely, for the alternation-bias group, this probability returned from a 1-back alternation effect (FH: *M* = -5.0%, *t*_*24*_ = 7.32, *p* < 10^−7^; CM: *M* = -5.4%, *t*_*15*_ = 4.93, *p* < 10^−4^) to the chance level prior to 1-back (FH: *M* = -0.3%, *p* > 0.05; CM: *M* = -0.4%, *p* > 0.05) as it increased slightly thereafter. Only the models with exponential hysteresis (2E1 and 2CE1) could match the shapes of the action-history curves, and the addition of constant bias made the correspondence even more precise. Concerning its pitfall of mimicry, constant bias alone (2C) manifests as an across-trial increase in the probability of repetition that superficially resembles the multitrial signature of an extended hysteresis trace.

**Fig 13:**
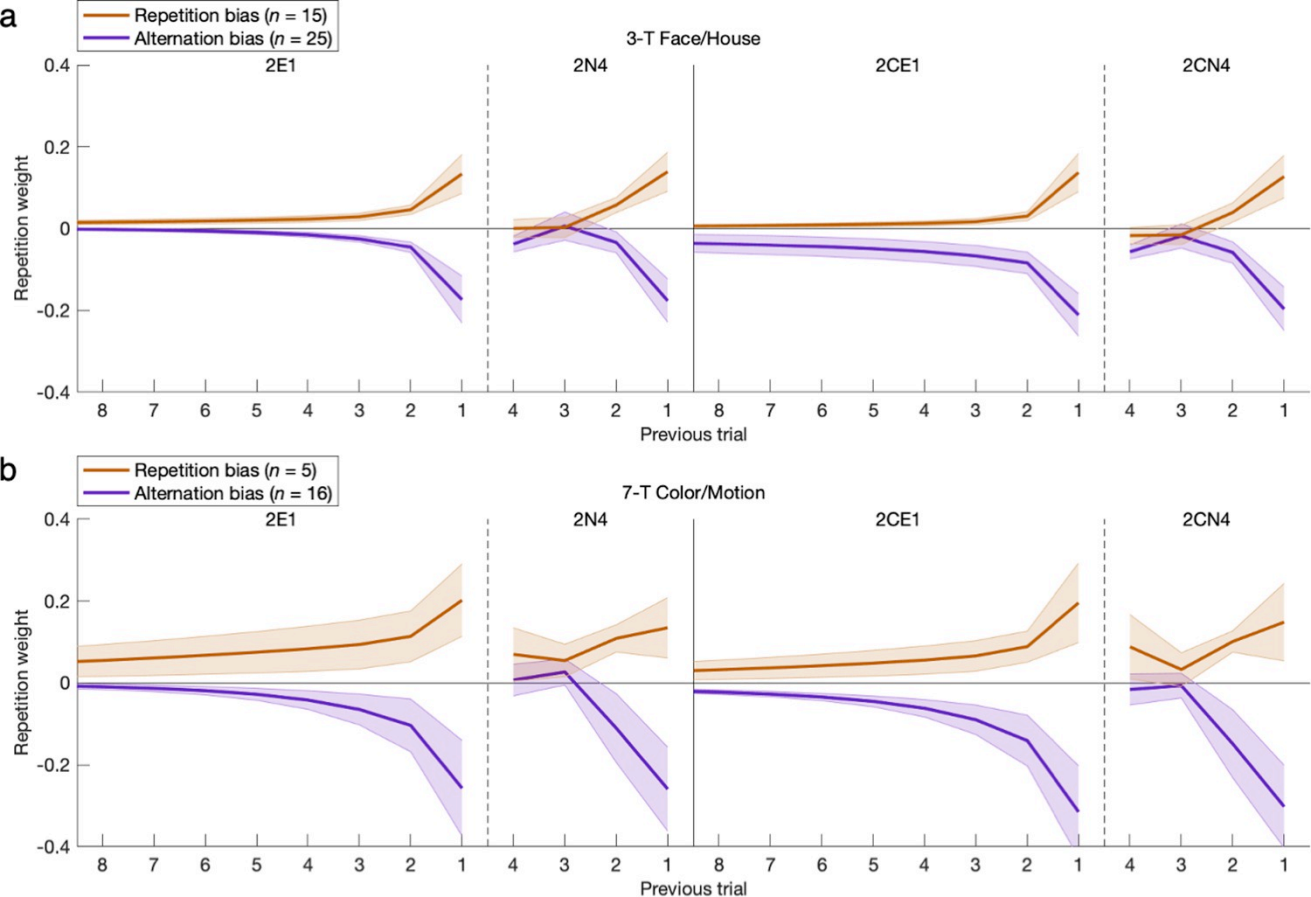
Hysteresis parameters with exponential or nonparametric models. The fitted parameters of the GRL model with either exponential or 4-back hysteresis are plotted as repetition weights (or alternation if negative)—simply *β*_*n*_ for *n*-back models or the corresponding weights *β*_*1*_*λ*_*H*_^*n*-*1*^ in the exponential function. Action-specific effects are better illuminated here by explicitly factoring out effects of RL and GRL within the comprehensive model. There is close correspondence between these parametric (2E1 and 2CE1) and nonparametric (2N4 and 2CN4) implementations of hysteresis for at least the first two trials back. The need for a scope extending beyond 1-back demands more than one free parameter, and a proper hysteresis trace with exponential decay yields an even better fit than a scope of 2-back due to subtle effects from 3-back and beyond. As further evidence of interactions among parameters, omission of constant bias (2E1 or 2N4) consistently inflated the modeled repetition weights as they were forced to attempt to mimic the necessary third parameter for constant bias. Altogether, the CE1 adjunct is essential. Error bars indicate standard errors of the means.

To better interpret the preceding model-independent time courses, the fitted parameters of the GRL model with either exponential or *n*-back (i.e., 4-back) hysteresis provide context by explicitly factoring out confounds in constant bias as well as the effects of value-based learning (**Fig 13**). (The selection of 4-back is only for comparison of action-history curves, as the corrected fit of the 9-parameter 2CN4 model was actually worse than that of 2CN2 after adding two more free parameters.) This juxtaposition of parametric and nonparametric implementations of hysteresis revealed notably close correspondence for at least the first two trials back. However subtle the correspondence may be for decaying 3-back and 4-back effects, the superior overall fit of the exponential model relative to a simpler 2-back model (2CN2) already indicated the persistence of collectively significant cumulative effects from 3-back and beyond. Moreover, omission of constant bias (2E1 or 2N4) consistently inflated all of the modeled repetition weights, revealing the source of the mimicry between constant bias and repetition—especially in the persistent exponential form—that was alluded to with posterior predictive checks. The 3-parameter adjunct of constant bias and exponential hysteresis proves necessary as well as largely sufficient to distill the action-specific aspects of individual behavioral profiles.

### Different forms of bias and hysteresis versus learning performance

The first set of analyses originally split the three levels of learning performance without splitting directions of action biases, whereas the second split directions of bias across Good and Poor learners without splitting levels of learning performance. For this final stage, participants were further divided into six subgroups that separated the two directions of either form of bias as well as the three levels of learning performance—this time also plotting the two directions for previously omitted Nonlearners. There are statistical limitations with this next degree of granularity, which left some of the subgroups with a small sample, but these intersectional subgroups are worth consideration even if only to verify that the main effects essentially extend to this level as well.

With respect to the first set of original findings, action bias and hysteresis were significant for Good learners but even more pronounced for Poor learners and Nonlearners (**Figs 6 and 7**). Second, 2CE1 simulations modeled with constant bias and exponential hysteresis could replicate the directions and magnitudes of empirical action-specific effects both qualitatively and quantitively (**Figs 8 and 9**). Notwithstanding the lack of statistical significance in a few of the smallest samples, these trends from either two or three groups consistently held true with the scrutiny of their interface within the six subgroups (**Figs Q and R in S1 Text**).

### Alternatives to state-independent action hysteresis

With the primary model comparison establishing that the 2CE1 model has the ideal architecture among the 72 models compared thus far, what follows are other possibilities that could be considered instead of or in addition to state-independent action hysteresis for comparable effects and possible confounds. In other words, these factors could ultimately relate to some form of repetition or alternation across the sequence of action choices. The list of alternative features includes state-dependent action hysteresis *H*_*t*_*(s*_*t*_,*a)* (cf. [21]), state-independent action value *Q*_*t*_*(a)*, confirmation bias in learning that weighs positive outcomes over negative with the constraint *α*_*N*_ < *α*_*P*_ (i.e., only optimism), or asymmetric learning rates with flexibility in the possibilities for *α*_*N*_ ≠ *α*_*P*_ (i.e., optimism or pessimism).

Parsimony is paramount here, and none of these alternatives are as parsimonious as basic hysteresis that is both outcome-independent and state-independent. Take, for example, certain instances of action repetition: Rather than default attribution to a more general optimistic confirmation bias for learning [64–68], first-order perseveration may offer a more parsimonious explanation for some observations. As mentioned for RL, confirmation bias can translate to an asymmetry in learning rates favoring positive over negative outcomes [69–78]—but at the cost of greater susceptibility to overfitting relative to state-dependent or state-independent hysteresis [42,44,46,79–81], which can manifest its own sort of outcome-independent confirmation bias (see Discussion). (Moreover, as option values become relative in the action policy, the action generalization of GRL can also achieve effects comparable to what asymmetric learning rates might otherwise produce. This point is beyond the present scope but illustrates the broader issue of compounding complexity across the many possibilities that a model could incorporate.)

**Fig 14:**
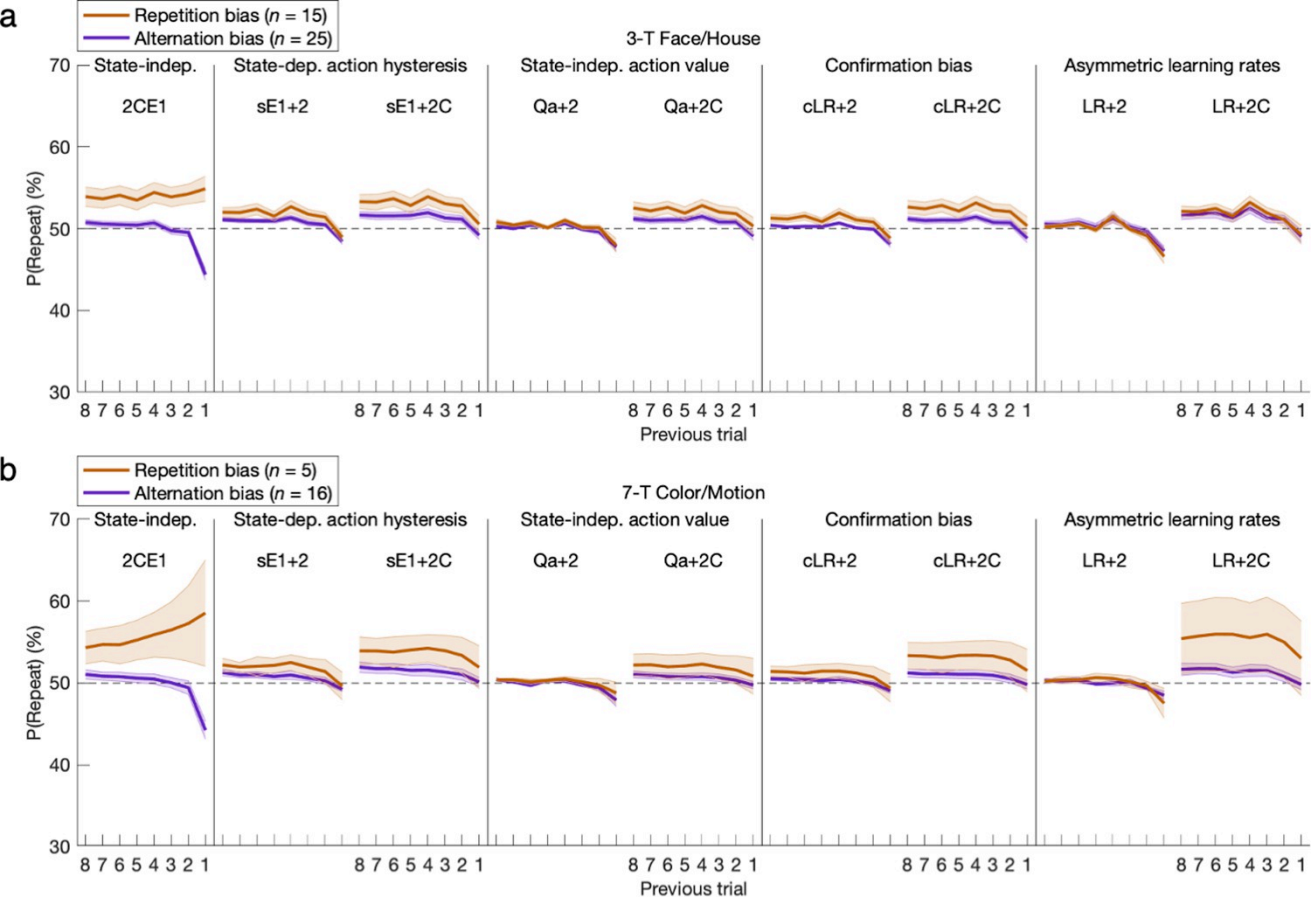
Alternatives to state-independent action hysteresis. Compare to Fig 12. To falsify alternative hypotheses concerning the origins of the apparent effects of state-independent action hysteresis *H*_*t*_*(a)* (“2CE1”), the model comparison was first extended to test substitution of state-dependent action hysteresis *H*_*t*_*(s*_*t*_,*a)* (“sE1+2C”), state-independent action value *Q*_*t*_*(a)* (“Qa+2C”), confirmation bias in learning with the constraint *α*_*N*_ < *α*_*P*_ (“cLR+2C”), or asymmetric learning rates with no constraint for *α*_*N*_ ≠ *α*_*P*_ (“LR+2C”). As expected, none of these alternatives were capable of generating the original action-history curves that only state-independent action hysteresis could produce.

The initial round of analyses for this extended model comparison began with substitutions of the factors of interest so as to test—and presumably falsify—their alternative hypotheses for the origins of repetition and alternation biases that state-independent hysteresis has been shown to account for with the posterior predictive checks above. Qualitative falsification was indeed robust for all four alternatives, such that none of these model features were capable of generating the original action-history curves that only state-independent action hysteresis could produce (**Fig 14 and Fig S in S1 Text**). These falsifications were hypothesized a priori in consideration of the following conceptual distinctions.

First, state-dependent hysteresis (“sE1+2” or “2sE1”) would not align with state-independent hysteresis because the four states were rotated in sequence such that there were variable numbers of trials between the origins and consequences of state-dependent effects. In keeping with this point, only a subtle repetition effect emerged after two trials back. For the original repetition-bias group, the effect sizes were nonexistent for one trial back and quantitatively insufficient from two trials back onward. Furthermore, for the original alternation-bias group, the emergent repetition effect was actually counterproductive such that it pointed in the opposite direction.

Second, state-independent action value (“Qa+2”) is unlike state-independent action hysteresis inasmuch as action value is outcome-dependent while action hysteresis is outcome-independent. In principle, there is potential for some degree of confounding if actions that are rewarded consistently end up being repeated consistently. However, in this controlled environment, state-independent action value had little impact on the action-history curves. For the second data set at least, there was a subtle alternation effect in both the original alternation-bias group and the original repetition-bias group—counterproductively for the latter.

Third, confirmation bias in learning (“cLR+2”) is generally limited to action repetition and is not only outcome-dependent but also state-dependent in the presence of rotating states here. Like with state-dependent hysteresis, there was only a subtle repetition effect from two trials back onward. However, unlike with state-dependent hysteresis, model simulations for the alternation-bias group did not exhibit a contrary repetition bias.

Fourth, a more flexible asymmetry in learning rates (“LR+2”), including either an optimistic confirmation bias or a pessimistic doubt bias, is again state- and outcome-dependent in the presence of rotating states here. Notably, not all participants in the repetition-bias group adhered to the rule of *α*_*N*_ < *α*_*P*_ in the absence of the constraint forcing confirmation bias. Hence the action-history curve for the repetition-bias group was not elevated above chance beyond 2-back as before with the constrained “cLR+2” result. Instead, the unconstrained asymmetry of “LR+2” produced a 1-back alternation effect for both groups—that is, also counterproductively for the repetition-bias group. With respect to the alternation-bias group, the model’s effect was insufficient in magnitude to quantitatively account for the actual effect observed.

### Extended model comparison

At this stage, each of the four alternatives had been falsified against state-independent hysteresis with its parsimonious account of the origin of the repetition and alternation effects of interest. The next issue to investigate was the extent to which an alternative feature might instead complement state-independent hysteresis for an even more complex model. Accordingly, the extended model comparison not only substituted these features—namely, state-dependent action hysteresis, state-independent action value, confirmation bias, and asymmetric learning rates—but also added them while crossing with constant bias and 1-back, 2-back, or exponential state-independent hysteresis (e.g., “sE1+2C”, “sE1+2CN1”, “sE1+2CN2”, “sE1+2CE1”) in subsets of eight models per alternative (**Table 4 and Figs S-W and Tables Q-U in S1 Text**). The eight models crossed with each alternative feature mirrored the previous reduction of the primary model comparison.

**Table 4:** Extended model comparison. Additional models were constructed with substitution or addition of the alternative features that might be expected to interact with effects of state-independent action hysteresis. Each alternative was fixed within a new subset of eight models building up to constant bias and exponential state-independent hysteresis (“-CE1”). Variations on substitution of state-dependent hysteresis in particular were also tested up to two parameters. Listed for each participant group are the best-fitting models (per AICc score) among each subset of eight models as well as the full set of 44 models. Although there appears to be some quantitative evidence suggesting state-dependent hysteresis in addition to state-independent hysteresis, the lack of qualitative validation with falsification leaves this quantitative result inconclusive. Hence the 2CE1 model remains preferred for a final model. “df” stands for degrees of freedom. See also Figs S-W and Tables Q-U in S1 Text.

Model comparison	df	Best fit	df	AICc
3FH: Good learner (*n* = 31)		sE1+2CE1	9	71.13
State-independent action hysteresis	7	2CE1	7	67.69
State-dependent action hysteresis	7	2CsE1	7	67.47
State-indep. + State-dep. action hysteresis	9	sE1+2CE1	9	71.13
State-indep. hysteresis + State-indep. action value	9	Qa+2CE1	9	64.92
State-indep. hysteresis + Confirmation bias	8	cLR+2CE1	8	68.32
State-indep. hysteresis + Asymmetric learning rates	8	LR+2CE1	8	69.57
3FH: Poor learner (*n* = 9)		sE1+2CN1	8	65.50
State-independent action hysteresis	7	2CE1	7	54.06
State-dependent action hysteresis	7	2CsE1	7	62.40
State-indep. + State-dep. action hysteresis	9	sE1+2CN1	8	65.50
State-indep. hysteresis + State-indep. action value	9	Qa+2CE1	9	50.32
State-indep. hysteresis + Confirmation bias	8	cLR+2CE1	8	54.67
State-indep. hysteresis + Asymmetric learning rates	8	LR+2CE1	8	54.73
3FH: Nonlearner (*n* = 7)		2CE1	7	16.04
State-independent action hysteresis	7	2CE1	7	16.04
State-dependent action hysteresis	7	2CsE1	7	5.94
State-indep. + State-dep. action hysteresis	9	sE1+2CE1	9	15.96
State-indep. hysteresis + State-indep. action value	9	Qa+2CE1	9	14.82
State-indep. hysteresis + Confirmation bias	8	cLR+2CE1	8	14.43
State-indep. hysteresis + Asymmetric learning rates	8	LR+2CE1	8	14.72
7CM: Good learner (*n* = 16)		sE1+2CE1	9	72.64
State-independent action hysteresis	7	2CE1	7	67.62
State-dependent action hysteresis	7	2CsE1	7	69.93
State-indep. + State-dep. action hysteresis	9	sE1+2CE1	9	72.64
State-indep. hysteresis + State-indep. action value	9	Qa+2CE1	9	66.07
State-indep. hysteresis + Confirmation bias	8	cLR+2CE1	8	67.07
State-indep. hysteresis + Asymmetric learning rates	8	LR+2CE1	8	67.55
7CM: Poor learner (*n* = 5)		Qa+2CN2	9	50.02
State-independent action hysteresis	7	2CN2	7	48.15
State-dependent action hysteresis	7	2CsE1	7	23.02
State-indep. + State-dep. action hysteresis	9	sE1+2CN2	9	49.30
State-indep. hysteresis + State-indep. action value	9	Qa+2CN2	9	50.02
State-indep. hysteresis + Confirmation bias	8	cLR+2CN2	8	46.05
State-indep. hysteresis + Asymmetric learning rates	8	LR+2CN2	8	46.78

The extended model comparison was applied both within and across the six subsets of eight models (with 44 models in total for the omnibus comparison). The first two subsets built up to constant bias and exponential hysteresis but distinguished the original subset with state-independent hysteresis (e.g., “2CE1”) from a new subset with state-dependent hysteresis (e.g., “2CsE1”). The remaining four subsets added each of the four alternative features as a fixed component crossed with the original subset of eight models building up to 2CE1 (e.g., “sE1+2CE1”, “Qa+2CE1”, “cLR+2CE1”, “LR+2CE1”).

Within every one of the model subsets, the group-level fitting results consistently favored the addition of the CE1 adjunct with all three of its parameters. In other words, the effects of state-independent hysteresis are indeed substantial, and these specific effects are not confounded with those of any of the alternative features because no alternative could eliminate the need for including state-independent hysteresis in order to adequately fit even the Good-learner groups.

Next comparing all 44 models across the six subsets at once, there actually was a notable improvement in overall quantitative fit with the addition of state-dependent hysteresis in particular (FH-G: sE1+2CE1, FH-P: sE1+2CN1, FH-N: 2CE1, CM-G: sE1+2CE1, CM-P: Qa+2CN2). Thus, among the four candidates, state-dependent hysteresis could merit highest priority as the next feature to explore as a possibility for an even larger 9-parameter model. However, despite quantitative gains for state-dependent hysteresis as well as other alternatives, there were still no qualitative improvements in any model-specific effect as would be necessary to falsify a base model having only state-independent hysteresis (**Figs T-W in S1 Text**).

With respect to the otherwise best candidate of state-dependent hysteresis, the absence of qualitative falsification means that its quantitative improvement in fit might actually reflect a spurious relation with residual nonlinearities in the dynamics of learning processes that, unlike hysteresis, are both state-dependent and outcome-dependent. Inevitably, learning is modeled less than perfectly with the current specification of GRL; to take but one example, there are necessary simplifications of a static rather than dynamic learning rate (cf. [81–93]) as well as static generalization weights [12]. The presently inconclusive evidence for state-dependent hysteresis is nevertheless suggestive of the possibility of qualitative validation in future paradigms designed to address follow-up questions about this and other plausible factors directly. However, the most definitive qualitative evidence here is limited to concluding that the final model remains the parsimonious 2CE1 model prioritizing state-independent hysteresis.

## Discussion

### Summary

These findings have illuminated action bias and hysteresis in the context of active RL so as to suggest that any such study of sequential behavior would benefit from due consideration of these essential variables. Even for some who learn properly, action-specific effects can be so substantial as to actually outweigh the learning effects under primary focus. The modeling inquired beyond basic RL, but two-dimensional GRL as well as constant bias and state-independent hysteresis (2CE1) could all be validated collectively for both quantitative and qualitative individual differences in highly idiosyncratic human behavior. Simpler alternatives to the 3-parameter CE1 adjunct for bias and hysteresis were systematically falsified with factorial model comparison and posterior predictive checks. Conversely, hysteresis models more complex than the 2-parameter exponential function of the CE1 adjunct were susceptible to overfitting. Moreover, an extended model comparison eliminated possible confounds in the form of state-dependent action hysteresis, state-independent action value, confirmation bias in learning, or asymmetric learning rates.

Recognizing each action-bias parameter as fundamental to the core modules of the mixture of experts and nonexperts, the practical costs of these degrees of freedom do not preclude parallel development of learning algorithms and theory. On the contrary, accounting for bias and hysteresis as sources of variance within and between individuals enhances the interpretability of finite behavioral data, which need to be modeled with the independence of each participant preserved. In environments without the symmetric counterbalancing of the present experiment, the limitations of a model with only learning can be even more substantial from spurious correlations between signatures of learning and nonlearning processes. To the extent that the action-specific aspects of bias and hysteresis would also be even more prominent in tasks with more engaging motor responses, proof of concept in this case of trivial motor demands suggests that these effects on choices and actions are as ubiquitous as they are parsimonious and should always be accounted for as a first priority—even with relevance to efficient artificial intelligence as a feature rather than a bug. While fitting at the level of individuals, building from the foundation of this base model—with at least five free parameters for basic RL (0CE1)—is critical to precisely test for whether and how each individual is learning as but a part of interacting with the environment.

### Constant bias and lateral bias

Here, the scope for cognitive modeling of motivated behavior is expanded beyond the abstraction of a disembodied brain. Considering that the motor system is the ultimate interface for the actions to be optimized, even low-level sensorimotor processes can constrain the embodied learner. This special case of a binary, bimanual choice task also translates constant bias to a lateral bias.

Although mostly overlooked as part of models of value-based learning, constant bias has occasionally been reported—with and without laterality [12,14–17,21,91,94–99] as well as between acting and not acting for a go/no-go task [100–105]. Even decision making that is not defined by learning—whether value-based [106] or perceptual [80,86,88–90,92,107–112]—can be affected by such stimulus-independent biases with a less obvious role for bias than would be assumed for skillful action-based decision making where physical aspects of action per se have explicit relevance [113].

The decision cost and action cost implicit in such a bias may reflect more than effector-specific motor bias—for example, not only selecting the left hand but also pressing the left button, engaging the left side of abstractly represented egocentric space, attending to the left hemifield of visual space, or embedding a chosen action within subsequences of left and right actions. Asymmetric costs and biases can be considered at all levels of sensorimotor perception, planning, preparation, and execution. Every participant in this neuroimaging study was right-handed for consistency, such that the coexistence of some leftward biases along with the rightward majority demonstrates the significance of not just handedness [114–118] but also a mixture of different levels of representation for nonexpert control.

Lateral biases, for example, can have diverse origins as well. For this sample of Westernized Americans—who are left-to-right readers, for example—eye-tracking studies have demonstrated that people with this cultural background share a propensity for attending to the left side of a display first [106,119–121]. Even more generally, low-level overrepresentation of the left hemifield has been implicated in tasks as basic as line bisection [122]. These biases are in keeping with the innate right-hemispheric dominance of visuospatial attention in the human brain [123–126]. Yet right-to-left (e.g., Hebrew) readers still learn through experience so as to instead exhibit rightward biases [127–129].

In essence, endogenous and exogenous sensorimotor biases are ubiquitous but not always straightforwardly interpretable beyond net effects reflecting a mixture of factors. For example, a leftward visuospatial bias might be at odds with a rightward motor bias in right-handed individuals performing this visuomotor task. There remains substantial ambiguity concerning the distributions of such biases and the relative influences of personal traits (such as handedness) or environmental factors (such as visuospatial cueing). Nevertheless, the key point established here is the need for flexible and fine-grained modeling of the possibilities for biases at the level of individuals.

### Bidirectional hysteretic bias

Maintaining the neutral terminology of “hysteresis” as “repetition” versus “alternation”, the model here begins with behavioral phenomenology before elaborating on broad unifying theory. That being said, the theoretical construct most often cited with respect to such hysteresis is perseveration, which describes how past responses are repeated regardless of whether or not it is beneficial to do so according to feedback for a new state of the environment [130–135]. Perseveration is linked with the conceptual umbrella of habit to some extent in not being goal-directed. However, habitual phenomena also tend to be more state-dependent, reward-dependent, time-dependent, and intentional than perseverative phenomena [6,136–144]. The literature has emphasized repetition over alternation as far back as the classic “law of effect”, which postulates repetition of rewarded responses but was also complemented by the “law of exercise” that postulates the repetition of past responses regardless of reward outcomes [64,65,137]. Yet an inverted sort of antiperseveration can also manifest with similarly inflexible tendencies toward rhythmic patterns of alternating responses [145–148].

The present study operationalizes perseveration at two levels: first-order, action-level perseveration for repetition of what an agent just did and second-order, policy-level perseveration for what an agent has been doing—either repetition or alternation depending on the circumstances. First-order perseveration aligns with the conventional usage of the term “perseveration” for action repetition in the context of RL, whereas the second-order perseveration emphasized here is less constrained and can result in action alternation as well for an environment such as the controlled one here. The present paradigm did not actually favor alternation per se but nonetheless facilitated it, such that a reward-maximizing policy would incidentally result in more frequent alternation but without any advantage in reward for arbitrary alternation. The hypothesis of second-order perseveration was apparently confirmed in the majority of participants with alternation biases rather than the default repetition biases more often mentioned in the RL literature. Yet, considered further, net effects in output frequencies can also reflect choice and action biases at different levels of representation.

Relatively low-level properties of the motor system can also contribute to alternation more so than repetition. More nonspecific alternation biases can manifest even in perceptual decision making, including neural correlates localized to motor cortex [147]. Whereas motor priming could favor repetition [149–155], motor fatigue could favor alternation if only for an opportunity to rest an effector and recover energy. The general phenomenon of repetition suppression [156,157] extends to the attenuation of signals in the brain’s motor areas—and especially premotor cortex—when actions are repeated [158–160]. Such effects may in part reflect the post-movement rebound of beta-band oscillations [161], which are also perhaps analogous to inhibition of return in sensory systems [162–164]. Tendencies toward alternating can also be apparent in arbitrary free choices made without the feedback of any outcome. Whether in expectation of statistical regularities or merely because of limitations in capacity for short-term memory or cognitive control, counterproductive repetition and alternation biases alike can even persist when a person is explicitly instructed to generate maximally random sequences as simple as mental coin flips [165–175].

Perseveration and action repetition in this context have been related to the functions of dopamine [20,144,176–181] (but see [101,182]) as well as perhaps serotonin [177,183] (but see [101]). The theory here can take into account the roles of dopaminergic systems for not only computations such as the reward-prediction error [184–186] but also motivation, vigor, effort, and skillful execution of movement [187–192].

### Multiple expert, semiexpert, and nonexpert controllers

The key dynamic variables in the present model are state- and outcome-dependent action value *Q*_*t*_*(s*_*t*_,*a)* and state- and outcome-independent hysteretic bias *H*_*t*_*(a)*. Having justified these two fundamental modules as a first priority with constant bias, there are then further possibilities to consider for additions to the mixture of expert and nonexpert controllers. As per the extended model comparison, *H*_*t*_*(a)* and *Q*_*t*_*(s*_*t*_,*a)* could in principle be complemented by state-dependent, outcome-independent hysteretic bias *H*_*t*_*(s*_*t*_,*a)* (cf. [21]) or state-independent, outcome-dependent action value *Q*_*t*_*(a)*. However, taking the qualitatively inconclusive gains in model fit observed here as an example, disentangling nonlinear dynamics for multiple types of learning and hysteresis at different levels of representation is nontrivial in practice.

Regarding *H*_*t*_*(s*_*t*_,*a)* for hysteresis that is outcome-independent but instead conditioned on the current external state, there can be an analogous conceptualization of a choice or action itself as a state-dependent reinforcer (i.e., autoreinforcer) motivating repetition in another positive-feedback loop—or a punisher motivating alternation for exploration. Like *H*_*t*_*(a)*, its counterpart *H*_*t*_*(s*_*t*_,*a)* can also be modeled with the accumulating hysteresis trace [21]. Along with the alternative of a replacing trace (see Methods), another more constrained implementation of hysteretic accumulation could be based on an action-prediction error (or choice-prediction error) with analogy to the reward-prediction error [40,42–47,96,143,144,178,181]. The action-prediction error has been framed as “value-free”, but this label and that of *H*_*t*_*(s*_*t*_,*a)* as “habit strength” (cf. [143]) may fail to represent a more endogenous form of subjective value such as with internal positive feedback for repetition (i.e., autoreinforcement) or negative feedback for alternation. The more neutral and bidirectional label of “hysteresis” is preferred here because “habit” not only overemphasizes repetition but also has more specific connotations of stimulus-response associations that may be more semiexpert than truly nonexpert—translating to biases made inflexibly persistent through reinforcement via the reward-prediction error as well [6,135–141,143,144]. Phenomena in the direction of state-dependent and state-independent repetition alike could also be relatable to choice-induced preference change as a reflection of a type of confirmation bias that resolves cognitive dissonance by disregarding feedback altogether [193–199], producing downstream effects comparable to those of confirmation bias with asymmetric learning rates. As discussed in the Results, there is considerable potential for confounds between *H*_*t*_*(s*_*t*_,*a)* and *Q*_*t*_*(s*_*t*_,*a)* as rewarded actions are appropriately repeated within a state, and likewise for *H*_*t*_*(s*_*t*_,*a)* and *H*_*t*_*(a)* if different states have overlap in sequences of actions and outcomes.

Regarding state-independent action value *Q*_*t*_*(a)*, this construct is conceptually constrained to align with repetition of rewarded actions. The most obvious interpretation conflates actions with low-level motor output—in contrast to the high-level goals of actions directed toward external stimuli [95,97,200–203]—but, under the proper circumstances, there could be cognitive and even strategic aspects to state-independent representations as well for semiexpert control. Sequential action representation under uncertainty can be more abstract than just motor control, such as with action chunking in response to working-memory load [204–206]. Concerning the challenge of adding *Q*_*t*_*(a)* to the mixture, a confound with *H*_*t*_*(a)* can ensue as rewarded actions are more often chosen. Moreover, a confound with *Q*_*t*_*(s*_*t*_,*a)* can also ensue if actions are rewarded similarly across different states.

### Levels of representation for decisions, choices, actions, and hysteresis

In contrast to biases more directly linked to motor representations, more abstract cognitive biases may impact sequential behavior as well. Higher-order choice-level biases—as opposed to action-level—can produce comparable effects of sequential dependence in paradigms where motor output is decoupled from perceptual [163,207–213] or value-based [214–217] decisions that do not require learning (i.e., choice hysteresis as opposed to action hysteresis). Complicating interpretation of choice bias or response bias yet further, effects of response history have been shown to parallel, interact with, and even conflict with effects of stimulus history at lower levels of representation in perceptual decision making [81,89,209,211,213,218–225].

For the phenomenology explored here, questions arise as to the contributions of different levels of representation and their integration in the parallelized modularity of the nervous system—ranging from the most abstract level of option choices to the most concrete level of physical motor output. With respect to constant bias *B(a)*, grounding the observed phenomena in the topology of visuospatial and motor representations is more immediately obvious because intrinsic action cost naturally corresponds to a bias that is both state-independent and sequence-independent. Hence an initial hypothesis here was that rightward biases would be more common among the exclusively right-handed participants, for example.

Whereas constant bias is more straightforward, the origins of even the basic hysteresis emphasized here are more nuanced. Yet that argument also primarily, albeit not exclusively, points to action-based representations—unlike with choice hysteresis as opposed to action hysteresis. First, there is the distinction between state-independent hysteresis *H*_*t*_*(a)* and state-dependent hysteresis *H*_*t*_*(s*_*t*_,*a)*, which have crucial differences between them despite both being outcome-independent. Whereas state-independent hysteresis may be primarily action-based, this may be less the case for state-dependent hysteresis.

As states of the task environment were rotating while the binary set of actions remained fixed (and time pressure was imposed), a state-independent action representation with tangible visuospatial and motor mapping is unlikely to entail as much abstract representation in terms of a high-level choice rather than action planning and execution. That is, the task incentivizes immediately mapping decisions directly to the space of actions and affordances [226–229], incurring no cost in doing so as long as the motor component of the task is simple and predictable.

In contrast, a state-dependent action representation would more plausibly invoke abstract choice representation to a substantial degree. Insofar as abstraction can be inherent to learning to map an action to the context of an arbitrary state with this sort of instrumental (or operant) conditioning [136,137], a state-aware controller would be making more of an abstract choice about the action than a state-blind controller would. Thus, state-dependent hysteresis could be less contained within action space and instead entail more abstract representation in choice space.

For other situations in which actions might not be as tangible and well-defined as they are in the present setting, greater degrees of abstraction away from action space and into choice space can become more plausible even for state-independent choice hysteresis. Further investigation will be needed for task demands across the spectrum ranging from the present extreme—that of the simplest one-to-one binary mapping across choices and actions as well as effectors and spatial locations—to the opposite extreme of a symbolic choice that must be made either in the absence of any information about subsequent action mapping or in the absence of action altogether (i.e., if only relevant for later actions). Yet the evidence herein is compatible with the majority of active-learning paradigms, where choices typically translate to actions directly and in a straightforward manner.

### Dynamics of hysteresis

The specific dynamics of choice or action hysteresis beyond 1-back have typically not been given consideration in previous empirical work with RL and hysteresis for behavior (cf. [79,80,94,95,97,98,101,203,230–242]). Thus far, some computational modeling [12,18,19,21,43,44,46,47,96,181,201,243,244] as well as simpler regression analyses with an autoregressive choice kernel or action kernel [17,20,58–61,245,246] have yielded differing time courses for hysteretic effects, but such findings tend to not be reported in detail.

Following the trends of artificial neural networks, deep learning [247–251], and deep RL [252–260], recent approaches to cognitive modeling have begun to utilize machine learning via the architecture of a recurrent neural network (RNN) [261–263]—such as with a long short-term memory (LSTM) unit [264] or a simpler gated recurrent unit (GRU) [265]—in an attempt to understand core computations for learning (i.e., beyond just nonlinear function approximation for state representation) [266–279]. Whereas such efforts pursue a data-centric approach leveraging predictive power as opposed to the present theory-centric approach leveraging explanatory power, it is the latter that has so far proven more effective for inference about empirical behavior (but see [87,280]). The mechanistic interpretability of a standard deep-learning approach (cf. [281–292]) is limited with nearly a black box and one typically not amenable to the individual differences here given model demands for data and dimensionality that are orders of magnitude larger. Hence, despite general merits of deep learning, the promise here is confronted by formidable challenges both practical and epistemological. At the very least, deep autoregressive neural networks with inputs for action or choice history—as well as state and reward histories—have begun to speak to not only the degree of nonlinear dynamical complexity but also the significance of sequential hysteresis across longer time scales in parallel with RL [267,270,271,274,277–279].

As part of the motivation for testing different hysteresis traces in the large-scale model comparison here, regression analyses without computational modeling have suggested possibilities for nonmonotonic reversals between short-term alternation and long-term repetition [17,20,58–60] or vice versa [20,61]. Although the dynamics of hysteresis may not always be so complex, sequential patterns can emerge from more than just neural activity persisting from previous trials. On the one hand, amplification of hysteresis over time is possible and can be attributed to working memory and its maintenance of past information [211] or instead to accumulating urgency signals [293] and their baseline activation for a response [294]. On the other hand, phenomena such as the diminishing of hysteresis with longer temporal intervals resonate with an account of sustained residual activity [214,216,295–301]. The exponential function evidenced here is a logical means to monotonic decay and also apt as a matched control against the similarly decaying effects of reinforcement across nonreinforced observations over time [12,18,19,21,43,47,96,243,244].

### The primacy of bias and hysteresis as well as individual differences

That the effects illuminated herein are so parsimonious and demonstrably extractable means that comparable studies of RL and other sequential tasks generally stand to benefit from considering bias and hysteresis as part of due diligence—even if the main focus of inquiry is directed elsewhere. Being more representative of actual behavior, the expanded 5-parameter base model 0CE1 aims to enhance parameter identifiability with respect to actual RL as opposed to action-specific components of variance that may mimic or otherwise obscure signatures of learning with spurious correlations [17,18,27,28,39–47]. Before making additional assumptions, parsimoniously imposing action-specific parameters with first priority can be beneficial as a sort of regularization for learning parameters that in practice are nontrivial to extract and estimate.

The present solution of a more comprehensive yet parsimonious model avoids compromising the independence of separate data sets, making it preferable to alternative small-data solutions finding recourse in regularization via fully group-level estimation (i.e., concatenating data sets or averaging parameters) or the intermediate approaches of empirical priors and hierarchical Bayesian modeling across participants [13,29,79,302–305]. From an idealized Bayesian-statistical perspective, compromising independence between individuals in this way mitigates putative measurement error from limited data. From a realistic perspective, however, measurement error and test-retest reliability are irrelevant and ill-defined here: A session of an experiment for a person and their internal state at the moment is a unique, nonrepeatable event—especially for dynamic learning, where model parameters are guaranteed to change over long timespans [47,105,306–320]. Across time, both learning and nonlearning modes for behavior can evolve or discretely alternate with dynamics that are as enigmatic as they are idiosyncratic [81,86,88–93]. In any case, anything resembling measurement error in behavior that is fitted with an incomplete model is not necessarily more substantial than modeling error [32], including that from omitted variables such as action bias and hysteresis.

As per the bias-variance tradeoff for the nonconvex optimization problem of model fitting, a reduction of variance in parameter fits with the group-level constraints of hierarchical Bayesian estimation necessarily incurs undesirable estimation bias both toward averages across individuals (i.e., shrinkage) and toward the specifications of parametric probability distributions [30–35,321,322]. Whereas a biased estimator will be guaranteed to show greater stability than an unbiased estimator, this property becomes disadvantageous when the biased estimator is less veridical. In a multidimensional parameter space, this estimation bias is exacerbated and can not only underestimate but also overestimate individual differences along a given dimension as a result of complex interactions among parameters constrained by outside data—for example, mimicry of a more constrained parameter by a less constrained one.

There is a more general epistemological problem with inference predicated on the strong assumptions of model validity and a common distribution for every individual from a random grouping of independent data sets, thereby speciously invoking the ecological fallacy [36–38]. The ecological (or population) fallacy is characterized by the principle that, even if a group in the aggregate is representative of the majority of the individuals within said group, any given individual or subgroup is not necessarily representative of the group at all. Hence, when assumed for the individual, assumptions based on group-level or hierarchical inference are inherently fallacious and invalidate potential conclusions about individual differences, including those applied in computational psychiatry and neurology [323–325] for computational phenotyping [29,316,318,319,326–328]. This point is missed in a cognitive-modeling literature now widely and unquestioningly adopting hierarchical Bayesian fitting—a trend motivated by the allure of results that, being biased, merely appear to be cleaner because of unverifiable assumptions about the unknowns of diverse brain states.

With independence instead preserved for each participant, the power of individual differences in computational modeling includes the means to model-based classification of individuals for hypothesis testing within, between, or across subgroups defined qualitatively and quantitatively by various dimensions of a model validated with posterior predictive checks [12,21]. Furthermore, if participants are grouped in advance—as with clinical studies, for example—this approach can address the initial classification in relation to model-based classification as well as model-based metrics across a continuum. More precise individual-level interpretability also extends to model-based analysis of neurophysiological data [329–331], including better estimation of computational signal dynamics within and between individual brains [12,21].

### The optimality of nonexpert control with lessons for ML and AI

From an apparently intuitive perspective, any bias or hysteresis in general might be viewed as interference that needs to be mitigated for optimal reward maximization with expert control. Perseveration in particular has a legacy of association with pathologized traits of compulsive behavior, brain lesions, and neurological disorders [20,97,130–132,233,332]. In a somewhat similar vein for the present study, the learners who performed best were not unbiased in this regard but did characteristically exhibit the least bias. Likewise, in experiments with extended training, the relative weight of choice biases tends to decline as learning performance improves over time [333,334]. Both repetition and alternation biases tend to be most robust when evidence is uncertain, confidence is low, and difficulty is high [207,224,238,333,335,336]. Among these factors, that of difficulty is most directly accounted for by the present model with point estimates for action values because these value estimates are rescaled by the nonlinearity of the softmax policy. That is, bias has greatest impact in the most locally linear vicinity of the intercept of the sigmoid psychometric curve as a function of value difference.

From another perspective, however, nonexpert biases are not suboptimal as part of a tradeoff for optimizing in favor of minimal cost, including computational costs of cognitive demands and motor control as well as sheer time. If uncertainty, unfamiliarity, or irrelevance trivialize a given decision, then choosing quickly according to low-cost biases by default would be optimal to mitigate energy expenditure and fatigue—even if fast responses could not affect the reward rate. Although additional complexities of dynamical decision making [293,337–341] are presently abstracted away for tractability, a speed-accuracy tradeoff [342,343] was evident both within and between participants in the data sets here [12]: More difficult decisions were slower, and across individuals, decisions made by better learners were slower as well. Altogether, the effortful aspects of task engagement can be integrated into the common currency of the cost of control [344–356]. Internal cost-benefit analysis also weighs these costs against reward incentives to determine the level of motivation to effortfully leverage expertise rather than defer to more efficient nonexpert control. Aside from the uncertainty in learning, the monetary incentivization in an experiment tends to be low in subjective value and can be reflected in low levels of motivation and arousal as well as effort and attention.

In contrast to its associations with suboptimality, perseveration has also been framed as adaptive policy compression amid a tradeoff between maximizing expected reward and minimizing the information-theoretic complexity of an action policy [135,176,179,205,206,357–359]. This principle can be extended to higher-order perseveration as well as action or choice bias in general. The dimensions exemplified here reflect how a more state- and outcome-dependent policy trades off being more rewarding for being more complex than a more state- and outcome-independent policy. In addition to undirected exploration with bias rather than variance (i.e., policy stochasticity for the latter), even exploitation can be achieved both more efficiently and more effectively with choice bias as a semi-optimal heuristic for strategic satisficing [360–362] if appropriate for a given environment [81,277,363–365]. In other words, nonexpert biases can even be leveraged in a semiexpert fashion. Such a reward-compressibility tradeoff may offer an analogy with other biases of perceptual stability [208,213,221,223,366,367] or cognitive anchoring [368,369]: Both similarly leverage heuristics for efficiency—whether at the expense of veridical sensory representation or at the expense of precise statistical estimation.

Even low-level motor biases, which if disregarding their benefits in lower internal cost might otherwise be considered a disadvantage of embodiment, may also not be so disruptive as part of a tradeoff for which an embodied RL policy has greater potential for robustness in learning per se. Indeed, embodied RL for concrete actions can achieve greater fluency than disembodied RL for symbolic choices abstracted away from motor output [95,99,202]. Benefits of embodied learning may be facilitated by lesser working-memory demands and lesser overall demands from the topology of the action space as a cognitive map [370–373] more amenable to spatial and embodied representations in the neural circuitry of the basal ganglia and cortex [8,21,22,374–378].

In addition to endogenous choice and action biases, exogenous factors can also shape biases over time. For example, the environment here was structured to be conducive to an alternation bias via second-order perseveration. Adaptive bias has been suggested for actions, effectors, or spatial locations in experimental paradigms delivering rewards asymmetrically with distributions that are congruent or incongruent with respect to particular biases [99,106,111,210,212,213,300,301,333,379]. Adaptive control with the heuristics of a mixture policy would entail flexible leveraging or suppressing of action bias and hysteresis to strike a balance among various tradeoffs of bias and variance, speed and accuracy, energy and effort, benefit and cost, reward and compressibility, expertise and efficiency, or exploration and exploitation.

With analogies between animal learning [6–8,55–57,380–382] and machine learning [49–54,288,291,383–396], the theory of a mixture of experts is based on advantages of modular parallelism and conditional computation for balancing versatility and efficiency in optimal control. As with the mixture-of-experts (MoE) architecture per se (which has also proven effective for sparse scaling of a deep neural network), the scope of this consilient theory can be extended to systems of varying levels of expertise as well as nonexpert controllers and their numerous choice and action biases (cf. [12,21,81,86,88–92,94–96,98,99,143,144]). Benefiting from distributed control of decisions and actions across diverse levels of representation in the networks of the nervous system [227–229], a mixture of experts and nonexperts can dynamically mediate distinct subpolicies with the metacontrol of a manager for arbitration over the gated ensemble of modular learning and nonlearning processes. With adaptive computation for a given subpolicy, semiexpert or nonexpert controllers could be upweighted for conserving time and energy when incentivized, whereas expert learning algorithms could be downweighted for being evaluated as too costly to compute or insufficiently reliable for lack of information or fidelity at any given moment.

Reverse engineering such manifestations of the implicit wisdom of evolution yields a wellspring of inspiration for designing artificial intelligence. Although this computational modeling has primarily been tailored to human behavior and its neural substrates, the fundamental concepts are well-suited for interdisciplinary triangulation across the consilience of RL. With respect to an embodied robotic system, cost and reliability can be factored in for the state of the plant with its physical constraints in action sequences as well as demands for inference and decisions with minimal latency [256,258,260,397–404]—all with analogy to a nervous system characterized by not only metabolic constraints and memory constraints but also motor constraints and embodied cognition [226–229,405,406]. More generally, these insights extend well beyond robotics into all of control theory, machine learning, and artificial intelligence. The costs of time, energy, and computational resources are not limited to active RL and indeed can be found in any system for inference or control. Considering their ubiquity, variants of bias and hysteresis of any abstraction are essential to multiobjective optimization in a resource-limited but resourceful agent—one who is effectively a mixture of agents and at that a mixture of experts and nonexperts.

## Methods

### Ethics statement

Including functional MRI (fMRI), participants provided informed written consent according to protocols approved by the Institutional Review Board of each of six scanning sites—namely, the California Institute of Technology; Columbia University; New York University; the University of Pennsylvania; the University of California, Santa Barbara; and the University of Southern California.

### Preface

In this second report, only the details most relevant for the present purposes are included here. Additional details of the study, including neuroimaging, can be found in the original report for these data sets [12]. Incidentally, “3 T” and “7 T” refer to field strength for the respective MRI scanners.

### Participants

Forty-seven (male:female = 27:20; age: *M* = 25.5 y, *SD* = 4.9 y) and twenty-two (male:female = 12:10; age: *M* = 28.0 y, *SD* = 6.0 y) human participants volunteered for the 3-T Face/House and 7-T Color/Motion versions of the study, respectively. The 3-T Face/House version was itself multisite, being conducted at five separate facilities for magnetic-resonance imaging (MRI) where participants were recruited from the respective universities and local communities of each laboratory. All participants were screened for MRI contraindications; all were right-handed and generally healthy adults between 18 and 43 years old. Participants in the 7-T Color/Motion version were also screened for color blindness. Upon completing the study, participants were paid $10 for minimizing head movement plus the amount of money earned within the task as the main incentive.

### Experimental procedures

A hierarchical reversal-learning task [12] delivered probabilistic outcomes for combinations of categorized states and conditional actions with reward distributions changing across 12 blocks of trials. Note that **Fig 1A** (showing only one state category) does not actually represent a possible sequence of trials (see **Figs A and B in S1 Text**) because the purpose of the figure is instead to conceptually illustrate action bias and hysteresis. To represent each active state (a two-armed contextual bandit), four new cues were assigned randomly every run with two pairs of images each respectively drawn from two state categories. In the version of the experiment incidentally conducted with a 3-T MRI scanner, these categories were faces and houses (images in Fig 1 courtesy of [407]).

At the onset of each episodic (i.e., separate) trial, one of four predictive cues was presented with equal probability, but trials were also ordered in a series of randomized and counterbalanced quartets that each included four cues representing separate states. These quartets were constrained such that a cue never appeared in consecutive trials. The onset of a trial was marked by a face or house image appearing. The participant was allotted 2 s to respond to this active state by pressing one of two buttons with the corresponding index finger of either the left or right hand. A fixed interstimulus interval (ISI) of 3 s separated the cue and the outcome.

The transition probabilities for the action given the state determined whether the outcome following the ISI was a rewarded state or a nonrewarded state. Delivery of an actual reward of $0.30 was symbolized by an image of a dollar sign for 1 s, whereas a scrambled dollar sign signified an absence of monetary reward for that trial. The duration of the jittered intertrial interval (ITI) was drawn without replacement within a run from a discrete uniform distribution ranging from 3 to 7 s in increments of 41.7 ms. If the participant failed to respond in time, the nonrewarded outcome appeared immediately as the fixation cross turned red for 1 s; the ISI would then be merged with the subsequent ITI.

Twelve blocks of trials were defined by permutations of three experimental conditions, The first condition for category value had three possibilities also counterbalanced within a run. This condition determined whether the face category had greater, lesser, or equivalent value relative to the house category. For the unequal conditions, the category with greater value included reward probabilities of 62.5% and 100%, whereas the category with lesser value included reward probabilities of only 43.75%. For the equal condition, both categories included reward probabilities of 43.75% and 81.25%. These exact probabilities were all divisible by sixteenths and so were evenly split between two 32-trial blocks with 8 trials per state. (For the odd probabilities of 43.75% and 81.25%, the more-rewarded halves of the distributions were evenly distributed within a condition sampled across runs: The net probability of 43.75% (7/16) was the average of 37.5% (6/16) and 50% (8/16), and net 81.25% (13/16) was the average of 75% (12/16) and 87.5% (14/16).) A nonzero reward probability was only assigned to one action per state, always leaving an alternative action with zero probability of reward. This complementarity between actions within a state was designed to reveal action generalization.

The second condition for state value had two possibilities partially counterbalanced with a 2:1 ratio within a run. This condition concerned which state (arbitrarily “A” or “B”) had the greater value within a category if the category included two different reward probabilities for a given block.

The third condition for action mapping had four possibilities. This condition concerned the mapping of a state category’s reward probabilities to actions, such that the two states (“A” and “B”) within a category always symmetrically provided rewards for opposite actions. The possibilities for this condition could be summarized across all four active states like so: “LR&LR”, “LR&RL”, “RL&LR”, or “RL&RL”, where the example of “LR&RL” can be expanded as “AL/BR & AR/BL” for the binary hierarchical metastates of the face and house categories, respectively. That is, “LR&RL” (or “AL/BR & AR/BL”) would mean that the left action is rewarded for face A and house B while the right action is rewarded for face B and house A. This complementarity between states within a category was designed to reveal state generalization.

Rather than sheer randomness in the design, which would especially limit interpretation of individual differences, meticulously controlled counterbalancing was crucial for eliminating confounds within and across individual sessions. For each participant, different conditions were randomized and counterbalanced to evenly distribute rewards for categories, states, and actions in a factorial design defining 12 blocks that included hierarchical reversals of instrumental learning. Four scanning runs including three blocks each and 32 trials per block amounted to 384 trials in total. (Prior to the actual experiment, the participant completed 10-trial practice sessions with separate stimuli both outside and inside the scanner.)

Nearly attaining a 3 x 2 x 4 design (“category value” x “state value” x “action mapping”) for the 12 blocks, the 3 x 2 and 3 x 4 crosses were fully counterbalanced while the 2 x 4 cross could only be partially balanced given the number of blocks. By virtue of this counterbalancing, choosing the same action for every single trial of the session was guaranteed to yield exactly half of the available rewards. Likewise, each state category preceded exactly half of the available rewards within each run. Moreover, with reward probabilities in units of sixteenths, each run included exactly or nearly one quarter of the rewards for the entire session. Yet the reward probabilities for state-action pairs fluctuated from block to block so as to facilitate variability in the dynamics of neural signals of interest. Across the session, what remained constant amid these fluctuations was the anticorrelational pattern between complementary actions within a state and between complementary states within a category. The categories were independent of each other without any such structured pattern between them.

Between blocks, the design was constrained for a single remapping to mark the onset of a new block within a run, where reversals of rewarded actions occurred for only one category at a time. The two categories were remapped in turn in a random order counterbalanced across runs, such that each category had exactly one between-block remapping per run. Although the participant was informed that the reward probabilities could change throughout the session, no explicit indications were provided as to how or when such changes might occur.

Regarding the 7-T Color/Motion version conducted in parallel, this second version of the experiment was mostly matched to the first but was not entirely identical. The main difference was that the 7-T version substituted dynamic colors and directions of motion in lieu of faces and houses as state categories. Moreover, these color and motion stimuli (4 in total) were not replaced every run as with the 3-T version’s faces and houses (16 in total). Although the two pairs of visual stimuli comprising the two categories instead remained constant across the entire session, the counterbalanced factorial design of the 3-T version was preserved such that the reward probabilities for the respective states still rotated as before.

### Computational modeling: Generalized reinforcement learning

Generalized reinforcement learning (GRL) [12] is a quasi-model-based extension of model-free reinforcement learning (RL) [1–3]. The description of GRL that follows here is simplified so as to shift the emphasis to details of action-specific bias and hysteresis in the model’s mixture policy for action selection (**Fig 1**). Importantly for the present purposes, GRL adds even more complexity to the mixture of experts and nonexperts. Incidentally, this complexity takes the form of intersecting dichotomies for associative versus discriminative generalization and state versus action generalization. This expansion of RL in parallel with the expansion of action bias and hysteresis serves to demonstrate the practical feasibility of simultaneously investigating more complex learning theory despite the costly degrees of freedom inherent to the added complexities of the nonlearning modules.

Neuroimaging analysis [329] and thus the original critic/Q-learner (CQ) model [12,21] are presently set aside for this analysis of the single-step cue-outcome task. This simplified version of the GRL model omits not only passive state-value learning—which would be via the critic module of the actor/critic architecture [408–410]—but also the temporal-difference (TD) prediction method [411–413]. Given the absence of the TD update here, the action-value learning that remains also makes no distinction between off-policy and on-policy methods such as in the Q-learning algorithm [414,415] and the state-action-reward-state-action (SARSA) algorithm [416], respectively.

The beginning of a run marks initialization of action values *Q*_*t*_*(s*,*a)* for all novel state-action pairs. As representing priors in the absence of previous associations would entail some kind of internal model, a naïve model-free agent initializes to zero [417]:

$$\forall (s,a):Q_{0}(s,a)=0$$

The rotating active states were initiated with the onset of each trial. Upon transitioning from an active state to an outcome state, a reward-prediction error (RPE) *δ*_*t+1*_ is determined by the discrepancy between the current action-value estimate *Q*_*t*_*(s*_*t*_,*a*_*t*_*)* and the subsequent reward (or lack thereof) *r*_*t+1*_ presented in the binary outcome state. The RPE would obey the same equation with any scalar reward as well:

$$\delta_{t+1}=r_{t+1}-Q_{t}(s_{t},a_{t})$$

As with any standard RL model, the value of the chosen state-action pair is updated according to the following delta-learning rule with a fitted learning rate *α* (for 0 ≤ *α* ≤ 1):

$$Q_{t+1}(s_{t},a_{t})=Q_{t}(s_{t},a_{t})+\alpha \delta_{t+1}$$

The equations thus far have described the basic RL model in its original form. In preparation for the following section on GRL, note again that the reward magnitude is fixed for this paradigm. Hence the cached action value *Q*_*t*_*(s*,*a)* effectively corresponds to the estimated probability of reward. To prevent the duplicated and relayed prediction errors of GRL from producing an illogical expected value for probabilistic binary outcomes (i.e., 0 ≤ *P* ≤ 1), the clipping function *f(x)* clips action value between zero and unity as an ad-hoc solution for this particular case where subjective value represents probability. Although reference dependence and normalization are mechanisms of relevance to value-based learning [418–421], the present paradigm is not suitably amenable to these complexities. Possibilities for alternatives to clipping are not considered for now inasmuch as a guaranteed improvement in fit in the absence of this constraint would presently be uninterpretable: Probability estimates above unity or below zero would be meaningless as probabilities per se, and a negative value would also correspond to negative valence despite an absence of punishment. When this neural model is applied to (computational) model-based neuroimaging analysis [12], these simulated signals have substantial implications for the interpretation of value signals in the brain, which would be maximized with certain reward and range from neutral to appetitive rather than including anything in the aversive range of valence. The *x* here refers to an updated value estimate prior to transformation:

f(x)=clip{x,[0,1]}

In contrast to previous RL models, the GRL model introduced here additionally applies a common RPE signal to learning about other state-action pairs within the same state as well as the same state category. Aside from generalization, the value of any state-action pair not encountered remains as is rather than being subject to decay or “forgetting” with potential for overfitting [43,58,200,422–426]. (For future investigation elsewhere, there are intriguing parallels to note in the mathematics of value decay versus counterfactual updating for non-encountered representations.) Presently, the two-alternative forced choice allows for a straightforward model of discriminative action generalization, such that the nonchosen action *a’*_*t*_ receives an inverse value update as the complement of the chosen action *a*_*t*_ (where prime notation refers to complementarity here). The variables *a*_*L*_ and *a*_*R*_ stand for the left and right actions:

$$a_{t}^{\prime }=\left\{ \begin{array}{l} a_{R},\;a_{t}=a_{L}\\ a_{L},\;a_{t}=a_{R} \end{array}\right.$$

This counterfactual update is regulated by a negative parameter for the action-generalization weight *g*_*A*_ (for -1 ≤ *g*_*A*_ ≤ 0) that modulates the original learning rate. Although associative action generalization is a possibility elsewhere, this parameter is not allowed to be positive here because the effective input to the softmax function is the difference between two action values—rendering overgeneralization across actions essentially indistinguishable from a mere absence of learning. The constraint that absolute generalization weights do not exceed unity first resolves the potential nonidentifiability issue of multiplied free parameters for generalized delta learning. Conceptually, this constraint also reflects the assumption—one shared with the eligibility trace of the “TD(λ)” algorithm [3,411–413,427,428]—that generalized RPE signals would not be relayed with greater gain than the original RPE signal but rather with lesser or equal gain. (In a different setting, this assumption might be relaxed under the appropriate circumstances.) As with the state generalization that follows, this action generalization is analogous to the temporal generalization of TD(λ) (see [12]):

$$Q_{t+1}(s_{t},a_{t}^{\prime })=f(Q_{t}(s_{t},a_{t}^{\prime })+g_{A}\alpha \delta_{t+1})$$

With only two states per category, state generalization entails an analogous formula where—in addition to the encountered state *s*_*t*_—the other, complementary within-category state *s’*_*t*_ receives a relayed value update. The variables *s*_*A*_ and *s*_*B*_ refer to state A and state B (arbitrarily designated as such):

$$s_{t}^{\prime }=\left\{ \begin{array}{l} s_{B},\;s_{t}=s_{A}\\ s_{A},\;s_{t}=s_{B} \end{array}\right.$$

This update is regulated by a state-generalization weight *g*_*S*_ (for -1 ≤ *g*_*S*_ ≤ 1) that modulates the learning rate. Unlike overgeneralization across actions, overgeneralization across states within a category can be detected here. That is, the agent could incorrectly operate as if the category itself were assumed to be a unitary state (*g*_*S*_ = 1), or the agent could at least partially conflate representations of exemplars within a category due to fuzzy boundaries (0 < *g*_*S*_ < 1). The present paradigm is characterized by anticorrelational linkage between states within a category. Hence a negative sign for *g*_*S*_ correctly produces discriminative generalization, while a positive sign for *g*_*S*_ incorrectly produces associative overgeneralization:

$$Q_{t+1}(s_{t}^{\prime },a_{t})=f(Q_{t}(s_{t}^{\prime },a_{t})+g_{S}\alpha \delta_{t+1})$$

The two factors of action generalization and state generalization interact multiplicatively to also update the complementary action for the complementary state. In the optimal case combining discriminative generalization across both dimensions (i.e., -1 ≤ *g*_*A*_ < 0 and -1 ≤ *g*_*S*_ < 0), this interactive state-action generalization weight would appropriately be associative (0 < *g*_*S*_*g*_*A*_ ≤ 1) for the one state-action pair that is correlated with the original pair rather than anticorrelated:

$$Q_{t+1}(s_{t}^{\prime },a_{t}^{\prime })=f(Q_{t}(s_{t}^{\prime },a_{t}^{\prime })+g_{S}g_{A}\alpha \delta_{t+1})$$

### Computational modeling: Mixture policy with bias and hysteresis

The learned *Q* values are inputs to a probabilistic action-selection policy *π*_*t*_*(s*,*a)* characterized by the Boltzmann-Gibbs softmax function and the Shepard-Luce choice rule as a discriminative model of decision making [3,23–25] rather than a generative model. The approximation of a softmax function—effectively with perfect subtraction between two alternatives here—has some limitations in accounting for nonlinearities in actual behavior due to the dynamics of underlying decision processes in the brain [340], but this simplification can suffice for the present purposes as a standard assumption for active-learning models.

In addition to an essential module for action value, the mixture policy here also incorporates inputs from modules for action-specific bias and hysteresis (**Fig 1**) [12,21]. Constant bias *B(a)* becomes a lateral bias between left and right actions in this case, whereas the dynamic hysteretic bias *H*_*t*_*(a)* (cf. [17,18]) maps repetition and alternation to positive and negative signs, respectively. These state- and outcome-independent action biases complemented the state- and outcome-dependent action values to determine the mixture policy’s action probabilities via the following softmax function with temperature *τ* (for *τ* > 0), which regulates the stochasticity of choices reflecting noise as well as exploration against exploitation [3,429–435]. This policy equation also reduces to a logistic function in the present case of a two-alternative forced choice:

$$\pi_{t}(s_{t},a)=P(a_{t}=a|s_{t})=\frac{\exp\left\{(Q_{t}(s_{t},a)+H_{t}(a)+B(a))/\tau \right\}}{\sum_{a^{*}}\exp\left\{(Q_{t}(s_{t},a^{*})+H_{t}(a^{*})+B(a^{*}))/\tau \right\}}$$

With *n-1* parameters for *n* available actions, constant bias is reduced to a single parameter for a binary action space such as the present one. The indicator function *I*_*R*_*(a)* is used for a lateral bias with the arbitrary convention that a positive sign for the parameter *β*_*R*_ corresponds to a rightward bias while a negative sign corresponds to a leftward bias:

$$I_{R}(a)=\left\{ \begin{array}{l} 0,\;a=a_{L}\\ 1,\;a=a_{R} \end{array}\right.$$

Avoiding the dummy-variable trap, the bias terms are then *β*_*R*_ for the right-hand action and null for the left-hand action:

$$B(a)=\beta_{R}I_{R}(a)$$

Modeling action hysteresis in terms of the dynamics of integrated repetition or alternation biases first requires an initialization of the hysteresis trace and its cumulative bias variable *H*_*t*_*(a)*:

$$\forall a:\;H_{0}(a)=0$$

A counter variable *C*_*t*_ is initialized at the beginning of each run to index the total number of actions performed within the run:

$$C_{0}=0$$

This action-counter variable is simply incremented with each action performed:

$$\forall a_{t}:\;C_{t}=C_{t-1}+1$$

Using this action index throughout the run, the indicator function *I*_*Ct*_*(a)* tracks action history:

$$I_{C_{t}}(a)=\left\{ \begin{array}{l} 0,\;a\neq a_{t}\\ 1,\;a=a_{t} \end{array}\right.$$

In its currently preferred form (“-E1” models such as 2CE1), the hysteretic bias is determined by its initial (i.e., 1-back) magnitude *β*_*1*_ and inverse decay rate *λ*_*H*_ (for 0 ≤ *λ*_*H*_ ≤ 1), where this base of the exponential function is notated as the complement of (i.e., unity minus) the exponential decay rate. A positive magnitude for this autocorrelation (*β*_*1*_ > 0) represents a repetition bias in favor of repeating previous actions, whereas a negative magnitude (*β*_*1*_ < 0) represents an alternation bias in favor of switching between actions. By conventions with analogy to the eligibility trace of TD(λ) [3], the hysteresis trace (i.e., action kernel) is specified as an accumulating trace rather than a replacing trace so as to not be overly constrained; the latter instead has an upper bound at *β*_*1*_ and disregards consecutive repeats (cf. [18]). Yet it is ultimately the difference between the cumulative hysteresis effects of competing actions that determines their net weight in the action policy. An accumulating repetition bias (*β*_*1*_ > 0, *λ*_*H*_ > 0) means that a repeated action would become even more likely to be repeated again with successive repetitions in a positive-feedback loop. Conversely, an accumulating alternation bias (*β*_*1*_ < 0, *λ*_*H*_ > 0) means that a second repetition would become even less likely. The exponential decay of a given action’s bias proceeds indefinitely with each action executed as the hysteresis trace is continually integrated into the cumulative hysteretic bias *H*_*t*_*(a)*:

$$H_{t+1}(a)=\sum_{i=0}^{C_{t}-1}\beta_{1}\lambda_{H}^{i}I_{C_{t}-i}(a)$$

The label of the preferred 2CE1 model stands for 2-parameter GRL (“2”), constant bias (“C”), and 1-back exponential hysteresis (“E1”)—that is, one degree of freedom preceding exponential decay. This model described thus far includes seven free parameters altogether—namely, learning rate *α*, action-generalization weight *g*_*A*_, state-generalization weight *g*_*S*_, softmax temperature *τ*, rightward (or leftward) bias *β*_*R*_, and initial magnitude *β*_*1*_ coupled with inverse decay rate *λ*_*H*_ for the exponential decay of the repetition (or alternation) bias. An additional 23 models of the 72 in the primary model comparison (**Table 2 and Table A in S1 Text**) were also nested within the 2CE1 model: X, XC, XN1, XCN1, XE1, XCE1, 0, 0C, 0N1, 0CN1, 0E1, 0CE1, 1, 1C, 1N1, 1CN1, 1E1, 1CE1, 2, 2C, 2N1, 2CN1, and 2E1.

Beyond 1-back hysteresis, the remaining 48 models extended *n*-back hysteresis with *N* free parameters *β*_*n*_ for *N* total previous actions. With reference to statistical fundamentals of generic sequence or time-series modeling, notation with “β” for bias reflects analogous notation for autoregressive and intercept terms corresponding to hysteresis and constant bias, respectively. The signed individual weights *β*_*n*_ each independently correspond to a bias in favor of repetition (*β*_*n*_ > 0) or alternation (*β*_*n*_ < 0) of the respective previous action from *n* actions back. The dynamic hysteretic bias *H*_*t*_*(a)* is more generally defined by this flexible equation to accommodate any combination of first *n*-back and then exponential hysteresis in series—here the first and second terms, respectively, summing backward across time again:

$$H_{t+1}(a)=\sum_{n=1}^{N}\beta_{n}I_{C_{t}-n+1}(a)+\sum_{i=N+1}^{C_{t}}\beta_{N}\lambda_{H}^{i-N}I_{C_{t}-i+1}(a)$$

### Computational modeling (extended): Alternatives to state-independent action hysteresis

At this point, the final 2CE1 model has been described in its entirety, and likewise for the other 71 models included in the primary model comparison. What follows are the details of models subsequently tested in an extended model comparison controlling for alternative features that might be expected to interact with the effects of the state-independent action hysteresis presently emphasized (**Table 1**).

### Computational modeling (extended): State-dependent action hysteresis

The first alternative feature considered as part of the extended model comparison was state-dependent hysteresis *H*_*t*_*(s*_*t*_,*a)* (cf. [21]) in contrast to state-independent hysteresis *H*_*t*_*(a)* as described above. The mathematical specifications of the hysteresis trace are entirely analogous with the incorporation of state dependence.

In this case, the cumulative bias variable is initialized for every state-action pair rather than just actions:

$$\forall (s,a):\;H_{0}(s,a)=0$$

The counter variable becomes a vector *C*_*t*_*(s*_*t*_*)* that instead indexes action counts separately for each state:

$$\forall s:\;C_{0}(s)=0$$

This action-counter variable is incremented with each action as before:

$$\forall a_{t}:\;C_{t}(s_{t})=C_{t-1}(s_{t})+1$$

The indicator function *I*_*Ct(s)*_*(s*,*a)* then tracks action history within each state:

$$I_{C_{t}(s_{t})}(s_{t},a)=\left\{ \begin{array}{l} 0,\;a\neq a_{t}\\ 1,\;a=a_{t} \end{array}\right.$$

In its pure exponential form (“sE1”), state-dependent hysteresis is determined by its initial (i.e., 1-back) magnitude *β*^*S*^_*1*_ and inverse decay rate *λ*_*S*_ (for 0 ≤ *λ*_*S*_ ≤ 1)—now for exponential decay across only the actions performed within a state:

$$H_{t+1}(s_{t},a)=\sum_{i=0}^{C_{t}(s_{t})-1}\beta_{1}^{S}\lambda_{S}^{i}I_{C_{t}(s_{t})-i}(s_{t},a)$$

In addition to the seven free parameters of the 2CE1 model, the extended “sE1+2CE1” model adds two more—that is, *β*^*S*^_*1*_ and *λ*_*S*_—for a maximum of nine parameters in total. However, in another subset of models matching the reduced model comparison (2sN1, 2sN2, 2sE1, 2CsN1, 2CsN2, and 2CsE1), state-dependent hysteresis was instead substituted for its state-independent counterpart to remain at most seven free parameters for that subset. The general equation for any combination of first *n*-back and then exponential state-dependent hysteresis is the following:

$$H_{t+1}(s_{t},a)=\sum_{n=1}^{N}\beta_{n}^{S}I_{C_{t}(s_{t})-n+1}(s_{t},a)+\sum_{i=N+1}^{C_{t}(s_{t})}\beta_{N}^{S}\lambda_{H}^{i-N}I_{C_{t}(s_{t})-i+1}(s_{t},a)$$

The extended “sE1+2CE1” model thus adds yet another term to the mixture policy:

$$\pi_{t}(s_{t},a)=\frac{\exp\left\{(Q_{t}(s_{t},a)+H_{t}(s_{t},a)+H_{t}(a)+B(a))/\tau \right\}}{\sum_{a^{*}}\exp\left\{(Q_{t}(s_{t},a^{*})+H_{t}(s_{t},a^{*})+H_{t}(a^{*})+B(a^{*}))/\tau \right\}}$$

### Computational modeling (extended): State-independent action value

In parallel along the dimension of state dependence, the next alternative feature was state-independent action value *Q*_*t*_*(a)* in contrast to state-dependent action value *Q*_*t*_*(s*,*a)* as described above. In this case, action values are initialized for not only state-action pairs but also actions per se:

$$\forall a:\;Q_{0}(a)=0$$

An action-specific RPE *δ*^*A*^_*t+1*_ is determined by the discrepancy between the state-independent action-value estimate *Q*_*t*_*(a*_*t*_*)* and the subsequent reward (or lack thereof) *r*_*t+1*_:

$$\delta_{t+1}^{A}=r_{t+1}-Q_{t}(a_{t})$$

Naturally, the value update for the chosen action follows an analogous delta-learning rule with an action-specific learning rate *α*_*A*_ (for 0 ≤ *α*_*A*_ ≤ 1):

$$Q_{t+1}(a_{t})=Q_{t}(a_{t})+\alpha_{A}\delta_{t+1}^{A}$$

In addition to the seven free parameters of the 2CE1 model, the extended “Qa+2CE1” model adds two more—that is, action-specific learning rate *α*_*A*_ (for 0 ≤ *α*_*A*_ ≤ 1) and action-specific value weight *w*_*A*_ (for 0 ≤ *w*_*A*_ ≤ 1)—to reach its maximum of nine parameters. (For the sake of tractability here, action generalization is presently omitted for state-independent action value, but either a shared or tenth parameter could have been added with a generalized RPE updating the state-independent value representation for the nonchosen action.) The weighting parameter between state-independent and state-dependent action value can be incorporated into the mixture policy like so:

$$\pi_{t}(s_{t},a)=\frac{\exp\left\{(w_{A}Q_{t}(a)+(1-w_{A})Q_{t}(s_{t},a)+H_{t}(a)+B(a))/\tau \right\}}{\sum_{a^{*}}\exp\left\{(w_{A}Q_{t}(a^{*})+(1-w_{A})Q_{t}(s_{t},a^{*})+H_{t}(a^{*})+B(a^{*}))/\tau \right\}}$$

### Computational modeling (extended): Asymmetric learning rates and confirmation bias

Rather than adding another module to the original mixture policy, another alternative feature that could similarly relate to the repetition or alternations of actions is asymmetry in learning rates between positive and negative RPE signals (*α*_*P*_ and *α*_*N*_ for 0 ≤ *α*_*P*_ ≤ 1 and 0 ≤ *α*_*N*_ ≤ 1). One subset of eight models (“LR+”) flexibly allowed for either an optimistic confirmation bias (*α*_*N*_ < *α*_*P*_) or a pessimistic doubt bias (*α*_*P*_ < *α*_*N*_), whereas another subset of eight models (“cLR+”) was constrained with an assumption of only confirmation bias if any asymmetry (*α*_*N*_ ≤ *α*_*P*_). Imposing the latter constraint was in keeping with precedent in the modeling literature that emphasizes choice or action repetition by way of optimism and confirmation bias, implying that these forces would ultimately override pessimism and doubt. This modification entailed the addition of only one free parameter for a maximum of eight total in the “LR+2CE1” and “cLR+2CE1” models.

With positive learning rate *α*_*P*_ and negative learning rate *α*_*N*_, the delta-learning rule is bifurcated with a conditional rule separating positive and negative RPE signals in this new equation:

$$Q_{t+1}(s_{t},a_{t})=\left\{ \begin{array}{l} Q_{t}(s_{t},a_{t})+\alpha_{P}\delta_{t+1},\;\delta_{t+1}>0\\ Q_{t}(s_{t},a_{t})+\alpha_{N}\delta_{t+1},\;\delta_{t+1}<0 \end{array}\right.$$

For the rewards of fixed magnitude here, the conditions of positive or negative RPE (*δ*_*t+1*_ > 0 or *δ*_*t+1*_ < 0) would be met in the presence or absence of reward (*r*_*t+1*_ = 1 or *r*_*t+1*_ = 0), respectively. Furthermore, with the extension of GRL, these separate learning rates likewise take effect for generalized RPE signals according to analogous conditional updates:

$$Q_{t+1}(s_{t},a_{t}^{\prime })=\left\{ \begin{array}{l} f(Q_{t}(s_{t},a_{t}^{\prime })+g_{A}\alpha_{P}\delta_{t+1}),\;\delta_{t+1}>0\\ f(Q_{t}(s_{t},a_{t}^{\prime })+g_{A}\alpha_{N}\delta_{t+1}),\;\delta_{t+1}<0 \end{array}\right.$$

$$Q_{t+1}(s_{t}^{\prime },a_{t})=\left\{ \begin{array}{l} f(Q_{t}(s_{t}^{\prime },a_{t})+g_{S}\alpha_{P}\delta_{t+1}),\;\delta_{t+1}>0\\ f(Q_{t}(s_{t}^{\prime },a_{t})+g_{S}\alpha_{N}\delta_{t+1}),\;\delta_{t+1}<0 \end{array}\right.$$

$$Q_{t+1}(s_{t}^{\prime },a_{t}^{\prime })=\left\{ \begin{array}{l} f(Q_{t}(s_{t}^{\prime },a_{t}^{\prime })+g_{S}g_{A}\alpha_{P}\delta_{t+1}),\;\delta_{t+1}>0\\ f(Q_{t}(s_{t}^{\prime },a_{t}^{\prime })+g_{S}g_{A}\alpha_{N}\delta_{t+1}),\;\delta_{t+1}<0 \end{array}\right.$$

### Model fitting and comparison

Whereas the original model comparison permuted models for all variants and reductions of RL and GRL (or fully model-based learning algorithms) [12], the primary model comparison here permuted fewer learning variants to instead combine these with varied implementations of action bias and hysteresis for 72 models in total (**Table 2 and Table A in S1 Text**). Specifically, this model comparison crossed factors for value-based learning, constant bias, *n*-back hysteresis, and exponential hysteresis. The first two factors for learning were limited to the cases of no learning (“X”) (*α* = *g*_*A*_ = *g*_*S*_ = 0), basic RL (“0”) (*g*_*A*_ = *g*_*S*_ = 0), 1-parameter GRL (“1”) (*g*_*A*_ = min{0, *g*_*S*_}, -1 ≤ *g*_*S*_ ≤ 1), and 2-parameter GRL (“2”) (-1 ≤ *g*_*A*_ ≤ 0, -1 ≤ *g*_*S*_ ≤ 1). (Note that 1-parameter GRL here still refers to two-dimensional GRL but with a shared single parameter.)

With respect to bias and hysteresis, the first main factor was the inclusion (“C”) or exclusion of the constant lateral bias *β*_*R*_, amounting to 36 models each for either possibility. The second main factor of hysteresis was further subdivided between *n*-back (“N”) hysteresis and exponential (“E”) hysteresis as nonparametric and parametric alternatives—but not mutually exclusive alternatives—with 40 pure *n*-back models, 8 pure exponential models, and 16 hybrid models. Nonparametric *n*-back hysteresis was tested up to 4 trials back in the presence of learning and up to 8 trials back in the absence of learning. Parametric exponential hysteresis was defined by exponential decay but, when hybridized, allowed up to 2 additional degrees of freedom for nonparametric weights on the most recent previous actions. For example, considering 2-parameter GRL models, *n*-back hysteresis was represented up to 4-back in pure form or 3-back in post-exponential form as 2N2, 2N3, 2N4, 2CN2, 2CN3, 2CN4, 2E2, 2E3, 2CE2, and 2CE3. The 2CN4 and 2CE3 models had the greatest number of free parameters with nine in total.

The competing models were all fitted to empirical behavior via maximum-likelihood estimation with independence maintained at the level of individual participants. Free parameters were optimized for overall goodness of fit to a participant’s sequence of actions with randomly seeded iterations of the Nelder-Mead simplex algorithm [436]. All modeling and fitting procedures were programmed with Matlab. The Akaike information criterion with correction for finite sample size (AICc) [62,63] provided a means to adjust for model complexity when comparing models that differ in degrees of freedom. Whereas the XCE1 model with constant bias and exponential hysteresis functioned as the null model for the original model comparison validating GRL [12], here the 0-parameter chance model “X” was used instead for the baseline explanatory power of a completely random action policy. Each free parameter was thus added incrementally with a requirement of statistical justification for every single one.

To further verify the discriminability of the preferred 2CE1 model with its seven free parameters, each fitted instantiation of the model was subsequently used to simulate a data set yoked to that of the respective participant. Another complete model comparison was conducted for these simulated data as a test of model recovery that would indicate whether the 2CE1 model could be discriminated reliably among both simpler and more complex alternatives. Tests of parameter recovery followed with the expectation that the fitted parameters for the simulated data would be correlated with the original fitted parameters for the empirical data that the simulations were derived from. For juxtaposition, these procedures were also repeated with simulations generated by the no-bias model “2” with only GRL.

Following the primary model comparison with its 72 models was the extended model comparison with 44 models spanning six subsets of eight models each. Moreover, each subset of eight models matched the original subset of eight initially highlighted within the primary model comparison—namely, 2, 2N1, 2N2, 2E1, 2C, 2CN1, 2CN2, and 2CE1. The first subset was the original subset itself. The second subset substituted state-dependent hysteresis in six of the original eight models (e.g., “2CsN1”, “2CsN2”, “2CsE1”). The remaining four subsets added each of the four alternative features—namely, state-dependent action hysteresis, state-independent action value, confirmation bias, and asymmetric learning rates—as a fixed component crossed with the original subset of eight models building up to 2CE1 (e.g., “sE1+2CE1”, “Qa+2CE1”, “cLR+2CE1”, “LR+2CE1”). Comparisons were made both within and across the six subsets.

### Data analysis

The group assignments for participants based on learning performance were maintained from the original model comparison [12]. The first measure of performance began with calculating overall accuracy as the proportion of actions for which the participant correctly chose the option that could result in delivery of a reward, excluding choices made for initial encounters with novel cues. Accuracy was compared with the chance level of 50% for each participant using a one-tailed binomial test. A subset of participants was initially set aside as the “Good learner” group if the accuracy score was significantly greater than chance [18]; subsequent modeling could also confirm that this label was appropriate for each individual within the group. The remaining participants with chance accuracy were subsequently assigned to either the “Poor learner” group or the “Nonlearner” group according to whether or not one of the original learning models could yield a significant improvement in goodness of fit relative to the XCE1 model, which was nested within each learning model while retaining bias and hysteresis but omitting any sensitivity to actual reward outcomes [21].

Individually fitted parameters of the 2CE1 model for action-specific effects were first tested against empirical measures for validation. Omitting the Nonlearner group for additional rigor, correlations were tested for between the rightward bias *β*_*R*_ and the probability of a right-hand action, between the repetition bias *β*_*1*_ and the probability of a repeated action, and between overall bias *|β*_*R*_*|+|β*_*1*_*|* and the probability of a correct action (hypothesizing an inverse relation). Linear regression was performed with one-tailed one-sample *t* tests and reported with the Pearson correlation coefficient as well as the Spearman rank-correlation coefficient to test for monotonicity. Given the exclusively right-handed participants, a net rightward bias (*β*_*R*_ > 0) was also tested for across each performance group with a one-tailed one-sample *t* test.

For the preferred 2CE1 model and the other 2-parameter GRL models nested within it (2, 2N1, 2N2, 2E1, 2C, 2CN1, and 2CN2), posterior predictive checks were conducted with simulated data sets that were yoked to the empirical data sets and analyzed in the same fashion after averaging across 1,000 simulations. For the first set of checks focusing on only pure GRL (“2”) and the full 2CE1 model, participants were initially divided according to the three levels of learning performance. Using one-tailed one-sample *t* tests, above-chance probabilities were tested for with respect to correct actions, right-hand actions, and alternated actions. (By design, alternation of actions was more frequent when actions were more correct.) The net right-hand effects in the Poor-learner and Nonlearner groups were compared to those in the Good-learner group with one-tailed independent-samples *t* tests. Analogous comparisons within and between groups were conducted for the raw measures of absolute lateral bias *|P(Right)-50%|* and absolute repetition-or-alternation frequency *|P(Repeat)-50%|*. Moreover, correlations were tested for across the continuous measure of accuracy rather than discrete participant groups.

Individuals across the two learner groups were first reclassified according to the 2CE1 model’s fitted result of either leftward bias (*β*_*R*_ < 0) or rightward bias (*β*_*R*_ > 0). Above-chance probabilities of either left-hand or right-hand actions were then tested for in empirical data as well as simulated data from the eight 2-parameter GRL models. These individuals were next reclassified according to the 2CE1 model’s fitted result of either alternation bias (*β*_*1*_ < 0) or repetition bias (*β*_*1*_ > 0). (Supplementary analyses further divided six intersectional subgroups as well, crossing the three levels of learning performance with either leftward versus rightward or alternation versus repetition.) The alternation-bias and repetition-bias groups were tested for above-chance probabilities of alternated and repeated actions, respectively. Post-hoc tests followed to check between groups in the event of trending but nonsignificant results within a group—in this case using one-tailed independent-samples *t* tests. The probability of repeating versus alternating was also conditioned on previous actions up to eight trials back. Posterior predictive checks for these action-history curves were generated for both the primary model comparison and the extended model comparison.

For psychometric functions, the first logistic-regression model represented the probability of a right-hand action as a function of the difference between the state-dependent action values *Q*_*t*_*(s*_*t*_,*a*_*R*_*)* and *Q*_*t*_*(s*_*t*_,*a*_*L*_*)* corresponding to right and left. The second model represented the probability of repeating the most recent action (independent of state) as a function of the difference between action values that correspond to repetition and alternation. To accommodate interindividual variability in the range of estimated values, differences in action values were normalized with respect to the maximum absolute value for each participant. Parameters for these mixed-effects models were first estimated at the level of individual participants and then assessed within each bias group by way of one-tailed one-sample *t* tests.

## Supporting information

S1 Text **Fig A. Task.** This schematic of the hierarchical reversal-learning task performed during fMRI scanning includes the probabilities of a rewarded outcome in one of 12 blocks. Following an intertrial interval (ITI) with a fixation cross, one of four paired states (i.e., cues) was presented with equal probability, prompting the participant to choose either the left-hand action (“L”) or the right-hand action (“R”). Confirmation of the action at the reaction time (RT) was followed by an interstimulus interval (ISI) and finally an outcome of either a monetary reward or no reward as feedback. The paired state categories were faces and houses for the 3-T version or colors and directions of motion for the 7-T version. Dotted arrows symbolize the two possible actions. Solid arrows represent equally or more likely state transitions, whereas dashed arrows represent less likely transitions. Arrow thickness corresponds to the weight of an outcome’s probability. **(b)** Only one action was rewarded per state, thereby facilitating discriminative action generalization. States were paired within a category as “state A” and “state B” such that opposite actions were rewarded between the two states, thereby facilitating discriminative state generalization. One of two possible arrangements for hierarchical reward structure (independent of probabilities) is shown here, corresponding to the face category for this example block: The upper face is “state A”, and the lower face is “state B”. There was no pairing between the independent categories. **(c)** The second possible arrangement is also shown for comparison. The two possibilities alternated within categories as this anticorrelational rule remained constant through reversals that remapped categories between blocks. For an optimal learner, this binary metastate determines the cognitive map or model of generalizable task structure, which for a proper (cognitive) model-based algorithm is an explicit model but for generalized reinforcement learning is an implicit model. This figure corresponds to Fig 1 of the original report [12]: https://doi.org/10.1002/hbm.25988.
**Fig B in S1 Text. The “generalized reinforcement learning” (GRL) model.** Representative dynamics of value signals and learning signals generated by the GRL model are shown for the final participant in the Good-learner group of the 3-T Face/House data set. Parameters were assigned as follows for this participant: *α* = 0.318, *g*_*A*_ = -0.710, *g*_*S*_ = -0.808, *τ* = 0.408, *β*_*R*_ = 0.178, *β*_*1*_ = -0.067, and *λ*_*H*_ = 0.753. Tracking the probability of reward for the left and right actions (blue and red lines, respectively) in each of four active states, the model’s estimates of action values *Q*_*t*_*(s*,*a)* (solid lines) are plotted along with actual values (dashed lines) over the course of 12 blocks. Plotted below these value signals are time courses of the corresponding action-value-prediction error (AVPE) *δ*^*Q*^_*t+1*_ signals, which represent a distinct type of reward-prediction error (RPE) along with the state-value-prediction error (SVPE) *δ*^*V*^_*t+1*_ (cf. [12,21]. However, throughout this report, the usage of the generic term “RPE” and its variable “*δ*_*t+1*_” with no superscript—rather than “AVPE” and “*δ*^*Q*^_*t+1*_”—is due to omission of the neural model’s SVPE here. Discriminative state and action generalization are evident with counterfactual updates of values for the three nonexperienced state-action pairs within a category. These additional updates occur despite only one state-action pair being experienced with feedback. Each colored tick mark denotes an occurrence of the respective action. This figure corresponds to Fig 7A of the original report [12].
**Table A in S1 Text. Model parameters (unrolled).** See Table 2. Models are listed individually here.
**Fig C in S1 Text. Discriminability of the 2CE1 model: 3-T Face/House version.** Compare to Fig 2. Each fitted instantiation of the preferred 2CE1 model was used to simulate a data set yoked to that of the respective participant. The results from the empirical model comparison were replicated in silico as a demonstration of the discriminability of this 7-parameter model among both simpler and more complex alternatives ranging from 0 to 9 free parameters. Model recovery succeeded inasmuch as the 2CE1 model remained preferred among Good learners, and 2CE1 or its nonlearning analog XCE1 could be recovered for Poor learners or Nonlearners as well. See also Tables G, H, and I.
**Fig D in S1 Text. Discriminability of the 2CE1 model: 7-T Color/Motion version.** Compare to Fig 3 and Fig C. See also Tables J and K.
**Fig E in S1 Text. Discriminability of the no-bias model “2” with only GRL: 3-T Face/House version.** Compare to Fig C. The no-bias model “2” was recovered in lieu of the bias-and-hysteresis model 2CE1 when substituting data simulated with the no-bias model. This converse model recovery again demonstrates an absence of overfitting. See also Tables L, M, and N.
**Fig F in S1 Text. Discriminability of the no-bias model “2” with only GRL: 7-T Color/Motion version.** Compare to Figs D and E. See also Tables O and P.
**Fig G in S1 Text. Reduced model comparison for discriminability of the 2CE1 model: 3-T Face/House version.** Compare to Fig 4 and Fig C.
**Fig H in S1 Text. Reduced model comparison for discriminability of the 2CE1 model: 7-T Color/Motion version.** Compare to Fig 5 and Figs D and G.
**Fig I in S1 Text. Reduced model comparison for discriminability of the no-bias model “2” with only GRL: 3-T Face/House version.** Compare to Fig E.
**Fig J in S1 Text. Reduced model comparison for discriminability of the no-bias model “2” with only GRL: 7-T Color/Motion version.** Compare to Figs F and I.
**Fig K in S1 Text. Model comparison by bias category: 3-T Face/House version.** Compare to Figs 2 (panel d here) and 4 (a) and Figs C (e), E (f), G (b), and I (c). Participant counts for best-fitting models can also be grouped according to four categories: no bias (e.g., “2”), constant bias (e.g., 2C), hysteretic bias (e.g., 2E1), or both constant and hysteretic bias (e.g., 2CE1).
**Fig L in S1 Text. Model comparison by bias category: 7-T Color/Motion version.** Compare to Figs 3 (panel d here) and 5 (a) and Figs D (e), F (f), H (b), J (c), and K.
**Fig M in S1 Text. Confusion matrix and inverse-confusion matrix.** Compare to Figs K and L. The confusion matrix *P(Fit | Simulation)* corresponds to the probability (as a percentage) that simulated data from a given model are best fitted by either the model that actually generated the data or an alternative model. The inversion matrix *P(Simulation | Fit)* instead corresponds to the probability that a model generated the simulated data given that either the same model or an alternative model fitted the data best. **(a)** Limiting the model comparison to only the 2CE1 or “2” models with or without bias and hysteresis, model confusion is minimal as expected. **(b-c)** Expanding the model comparison with a binarized categorization of bias versus none for either 8 (b) or 72 (c) models does leave confusion less minimal as the models with bias outnumber the models without bias, but the expected trend of model recovery still holds true. **(d-f)** Results were replicated in the 7-T Color/Motion version of the experiment.
**Fig N in S1 Text. Parameter recovery with the 2CE1 model more accurate than recovery with the no-bias model “2” including only GRL. (a)** As described previously, the 2CE1 model was fitted to yoked simulated data that were generated with the 2CE1 parameters originally fitted to empirical data. Parameter recovery was especially robust for the Good-learner group across all seven free parameters, including *β*_*R*_, *β*_*1*_, and *λ*_*H*_ for action bias and hysteresis (*p* < 0.05). Although somewhat less robust, recovery of 2CE1 parameters was also successful for the Poor-learner group (*p* < 0.05 with the exception of *τ* from the first data set and *p* < 0.06 for *α* from the second data set). **(b)** The relative significance of bias and hysteresis was found to be greatest among Poor learners. Hence, if instead fitting the no-bias model “2”, the remaining four parameters needed for pure GRL (*α*, *g*_*A*_, *g*_*S*_, *τ*) were not significantly recoverable for the Poor-learner group (*p* > 0.05 with one exception for *g*_*S*_ from the second data set). **(c)** The *p* values for the correlations are plotted separately for Good (“G”) and Poor (“P”) learners when using either the 7-parameter 2CE1 model or the 4-parameter model “2”. **(d-f)** Results were replicated in the 7-T Color/Motion version of the experiment.
**Fig O in S1 Text. Action bias and hysteresis versus learning performance: Individual results.** Compare to Figs 6 and 7.
**Fig P in S1 Text. Hysteresis represented by sequences across trials.** Compare to Fig 12. The distribution of lengths of runs of consecutive repeated actions reveals hysteresis from another perspective. Alternation and repetition biases should result in shorter and longer runs, respectively, as only a model including hysteresis could replicate. Error bars indicate standard errors of the means.
**Fig Q in S1 Text. Constant bias and learning performance.** Compare to Figs 6, 7, and 8. Participants were further divided into six subgroups that separated the two directions of constant lateral bias as well as the three levels of learning performance. Constant bias should still be substantial for Good learners but should be even more pronounced for Poor learners and Nonlearners. Moreover, modeled bias in 2CE1 simulations should still both qualitatively and quantitively replicate the directions and magnitudes of empirical effects of laterality.
**Fig R in S1 Text. Hysteresis and learning performance.** Compare to Figs 6, 7, and 9. Participants were next subdivided with the two directions of hysteretic bias as the first factor crossed with learning performance. As with constant bias, hysteretic bias should still be substantial for Good learners but should be even more pronounced for Poor learners and Nonlearners. Likewise, modeled bias in 2CE1 simulations should still replicate the directions and magnitudes of empirical effects of hysteresis.
**Fig S in S1 Text. Substitution of state-dependent action hysteresis.** Compare to Figs 12 and 14. The alternative of state-dependent hysteresis *H*_*t*_*(s*_*t*_,*a)* was first substituted in place of state-independent hysteresis *H*_*t*_*(a)*. Following the original reduced comparison of eight models, here state-dependent action hysteresis was tested in its 1-back (2CsN1), 2-back (2CsN2) and exponential (2CsE1) forms. As expected because the four states (which this hysteresis depends on) were rotated in sequence, each form of state-dependent hysteresis by itself failed to match the action-history curves here.
**Fig T in S1 Text. Addition of state-dependent action hysteresis to state-independent action hysteresis.** Compare to Figs 12 and 14 and Fig S. State-dependent hysteresis *H*_*t*_*(s*_*t*_,*a)* in exponential form (“sE1+”) was subsequently added to the eight models from the original reduced model comparison with state-independent hysteresis *H*_*t*_*(a)* (2 through 2CE1). Considering that the 2CE1 model in its own right could parsimoniously account for all of these alternation and repetition effects, the expanded sE1+2CE1 model was not justified by any qualitative improvement in fit.
**Fig U in S1 Text. Addition of state-independent action value.** Compare to Figs 12 and 14. State-independent action value *Q*_*t*_*(a)* was added to the eight models from the original reduced model comparison with only state-dependent action value *Q*_*t*_*(s*_*t*_,*a)*. Again, the expanded Qa+2CE1 model was not justified by any qualitative improvement in fit.
**Fig V in S1 Text. Addition of confirmation bias.** Compare to Figs 12 and 14. A second learning rate was added to distinguish updates for positive and negative reward-prediction errors (*α*_*P*_ and *α*_*N*_). Models with confirmation bias (“cLR+”) in particular imposed the constraint *α*_*N*_ < *α*_*P*_ with an assumption of subjective optimism biased toward positive valence. The expanded cLR+2CE1 model was not justified by any qualitative improvement in fit.
**Fig W in S1 Text. Addition of asymmetric learning rates.** Compare to Figs 12 and 14 and Fig V. As before, a second learning rate was added to distinguish updates for positive and negative reward-prediction errors, but here the asymmetric learning rates *α*_*N*_ ≠ *α*_*P*_ had no constraint of confirmation bias such that pessimistic doubt bias was also a possibility. Even in this unconstrained form, the expanded LR+2CE1 model still was not justified by any qualitative improvement in fit.
**Table B in S1 Text. Model comparison: 3-T Face/House version (Good-learner group).** See Fig 2. Listed first for the 72 models fitted to empirical data are absolute scores for deviance and the corrected Akaike information criterion (AICc), where a lower score is better. These absolute scores were translated to residual goodness of fit relative to the null chance model “X”, where a higher score is better. Results with the best fit according to the AICc, which penalizes degrees of freedom, are highlighted with boldface and italics. “df” stands for degrees of freedom. The conventions for displaying this table also apply for Tables C-U.
**Table C in S1 Text. Model comparison: 3-T Face/House version (Poor-learner group).** See Fig 2.
**Table D in S1 Text. Model comparison: 3-T Face/House version (Nonlearner group).** See Fig 2.
**Table E in S1 Text. Model comparison: 7-T Color/Motion version (Good-learner group).** See Fig 3.
**Table F in S1 Text. Model comparison: 7-T Color/Motion version (Poor-learner group).** See Fig 3.
**Table G in S1 Text. Discriminability of the 2CE1 model: 3-T Face/House version (Good-learner group).** See Fig C.
**Table H in S1 Text. Discriminability of the 2CE1 model: 3-T Face/House version (Poor-learner group).** See Fig C.
**Table I in S1 Text. Discriminability of the 2CE1 model: 3-T Face/House version (Nonlearner group).** See Fig C.
**Table J in S1 Text. Discriminability of the 2CE1 model: 7-T Color/Motion version (Good-learner group).** See Fig D.
**Table K in S1 Text. Discriminability of the 2CE1 model: 7-T Color/Motion version (Poor-learner group).** See Fig D.
**Table L in S1 Text. Discriminability of the no-bias model “2” with only GRL: 3-T Face/House version (Good-learner group).** See Fig E.
**Table M in S1 Text. Discriminability of the no-bias model “2” with only GRL: 3-T Face/House version (Poor-learner group).** See Fig E.
**Table N in S1 Text. Discriminability of the no-bias model “2” with only GRL: 3-T Face/House version (Nonlearner group).** See Fig E.
**Table O in S1 Text. Discriminability of the no-bias model “2” with only GRL: 7-T Color/Motion version (Good-learner group).** See Fig F.
**Table P in S1 Text. Discriminability of the no-bias model “2” with only GRL: 7-T Color/Motion version (Poor-learner group).** See Fig F.
**Table Q in S1 Text. Extended model comparison: 3-T Face/House version (Good-learner group).** See Table 4. Results with the best fit within each subset of 8 models are highlighted with boldface and italics. Results with the best fit across all 44 models are also marked with asterisks.
**Table R in S1 Text. Extended model comparison: 3-T Face/House version (Poor-learner group).** See Table 4.
**Table S in S1 Text. Extended model comparison: 3-T Face/House version (Nonlearner group).** See Table 4.
**Table T in S1 Text. Extended model comparison: 7-T Color/Motion version (Good-learner group).** See Table 4.
**Table U in S1 Text. Extended model comparison: 7-T Color/Motion version (Poor-learner group).** See Table 4.
(PDF)

S1 Data Data. All data are included.
(ZIP)
