# Supplementary material for: Active reinforcement learning versus action bias and hysteresis: control with a mixture of experts and nonexperts
Source: PLoS Comput Biol. 2024 Mar 29;20(3):e1011950. doi: 10.1371/journal.pcbi.1011950 (PMC10980507; doi:10.1371/journal.pcbi.1011950)
Supplement: S1 Text — Fig A. Task. This schematic of the hierarchical reversal-learning task performed during fMRI scanning includes the probabilities of a rewarded outcome in one of 12 blocks. Following an intertrial interval (ITI) with a fixation cross, one of four paired states (i.e., cues) was presented with equal probability, prompting the participant to choose either the left-hand action (“L”) or the right-hand action (“R”). Confirmation of the action at the reaction time (RT) was followed by an interstimulus interval (ISI) and finally an outcome of either a monetary reward or no reward as feedback. The paired state categories were faces and houses for the 3-T version or colors and directions of motion for the 7-T version. Dotted arrows symbolize the two possible actions. Solid arrows represent equally or more likely state transitions, whereas dashed arrows represent less likely transitions. Arrow thickness corresponds to the weight of an outcome’s probability. (b) Only one action was rewarded per state, thereby facilitating discriminative action generalization. States were paired within a category as “state A” and “state B” such that opposite actions were rewarded between the two states, thereby facilitating discriminative state generalization. One of two possible arrangements for hierarchical reward structure (independent of probabilities) is shown here, corresponding to the face category for this example block: The upper face is “state A”, and the lower face is “state B”. There was no pairing between the independent categories. (c) The second possible arrangement is also shown for comparison. The two possibilities alternated within categories as this anticorrelational rule remained constant through reversals that remapped categories between blocks. For an optimal learner, this binary metastate determines the cognitive map or model of generalizable task structure, which for a proper (cognitive) model-based algorithm is an explicit model but for generalized reinforcement learning [file pcbi.1011950.s001.pdf]

Active reinforcement learning versus action bias and hysteresis: control with a mixture of experts and nonexperts

Jaron T. Colas[1,2,3]*, John P. O'Doherty[2,3]**, & Scott T. Grafton[1]**

[1]Department of Psychological and Brain Sciences, University of California, Santa Barbara, CA
[2]Division of the Humanities and Social Sciences, California Institute of Technology, Pasadena, CA
[3]Computation and Neural Systems Program, California Institute of Technology, Pasadena, CA

*Correspondence: jcolas@ucsb.edu
**Co-senior authors

2024, *PLOS Computational Biology*

https://doi.org/10.1371/journal.pcbi.1011950

Supporting Information:
Supplementary Figures and Tables

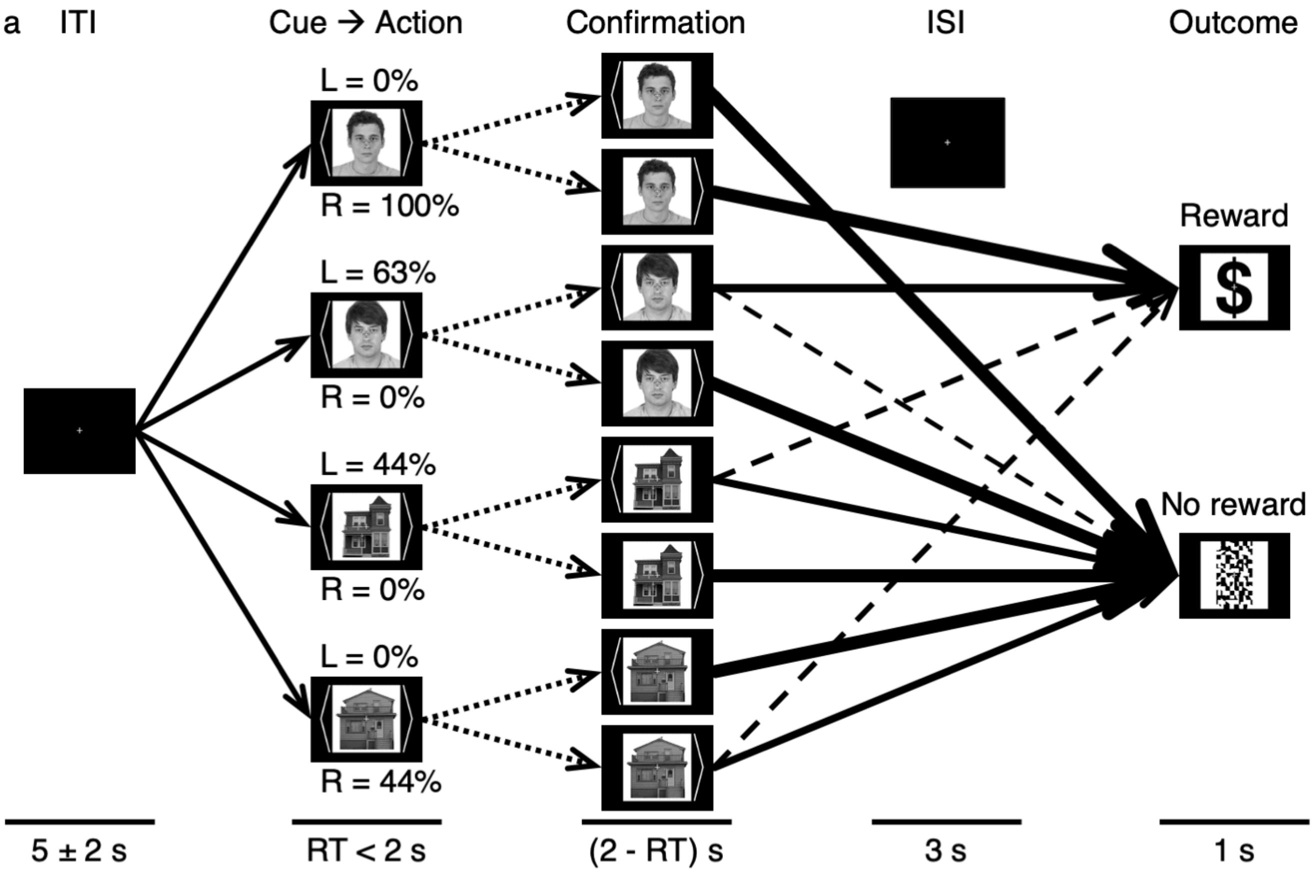

b

Action generalization

State generalization	Action L	Action R
**State A**	No reward	Reward
**State B**	Reward	No reward

c

	**L**	**R**
**A**	0	$
**B**	$	0

	**L**	**R**
**A**	$	0
**B**	0	$

Fig A. Task. (a) This schematic of the hierarchical reversal-learning task performed during fMRI scanning includes the probabilities of a rewarded outcome in one of 12 blocks. Following an intertrial interval (ITI) with a fixation cross, one of four paired states (i.e., cues) was presented with equal probability, prompting the participant to choose either the left-hand action ("L") or the right-hand action ("R"). Confirmation of the action at the reaction time (RT) was followed by an interstimulus interval (ISI) and finally an outcome of either a monetary reward or no reward as feedback. The paired state categories were faces and houses for the 3-T version or colors and directions of motion for the 7-T version. Dotted arrows symbolize the two possible actions. Solid arrows represent equally or more likely state transitions, whereas dashed arrows represent less likely transitions. Arrow thickness corresponds to the weight of an outcome's probability. **(b)** Only one action was rewarded per state, thereby facilitating discriminative action generalization. States were paired within a category as "state A" and

“state B” such that opposite actions were rewarded between the two states, thereby facilitating discriminative state generalization. One of two possible arrangements for hierarchical reward structure (independent of probabilities) is shown here, corresponding to the face category for this example block: The upper face is “state A”, and the lower face is “state B”. There was no pairing between the independent categories. **(c)** The second possible arrangement is also shown for comparison. The two possibilities alternated within categories as this anticorrelational rule remained constant through reversals that remapped categories between blocks. For an optimal learner, this binary metastate determines the cognitive map or model of generalizable task structure, which for a proper (cognitive) model-based algorithm is an explicit model but for generalized reinforcement learning is an implicit model. This figure corresponds to Figure 1 of the original report (Colas et al., 2022): https://doi.org/10.1002/hbm.25988

Colas, J. T., Dundon, N. M., Gerraty, R. T., Saragosa-Harris, N. M., Szymula, K. P., Tanwisuth, K., Tyszka, J. M., van Geen, C., Ju, H., Toga, A. W., Gold, J. I., Bassett, D. S., Hartley, C. A., Shohamy, D., Grafton, S. T., & O’Doherty, J. P. (2022). Reinforcement learning with associative or discriminative generalization across states and actions: fMRI at 3 T and 7 T. *Human Brain Mapping*, 43(15), 4750-4790.

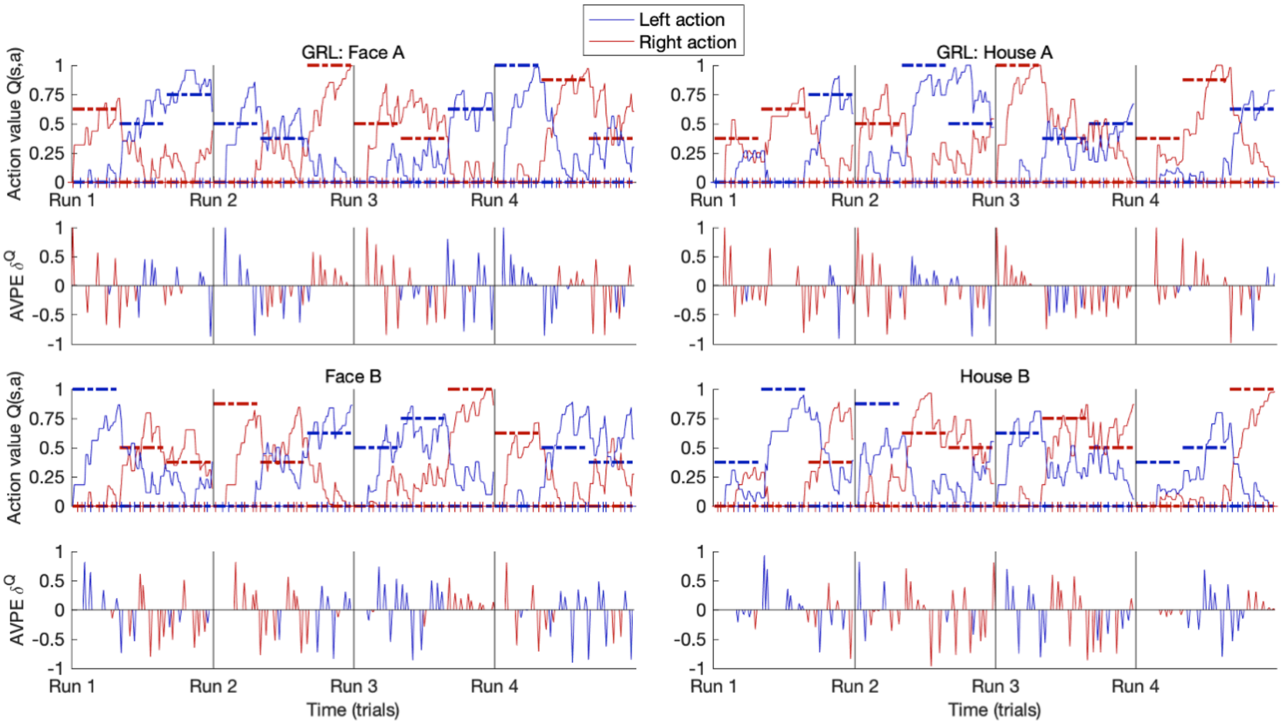

Fig B. The "generalized reinforcement learning" (GRL) model. Representative dynamics of value signals and learning signals generated by the GRL model are shown for the final participant in the Good-learner group of the 3-T Face/House data set. Parameters were assigned as follows for this participant: α = 0.318, g_A = -0.710, g_S = -0.808, τ = 0.408, β_R = 0.178, β_1 = -0.067, and λ_H = 0.753. Tracking the probability of reward for the left and right actions (blue and red lines, respectively) in each of four active states, the model's estimates of action values $Q_t(s,a)$ (solid lines) are plotted along with actual values (dashed lines) over the course of 12 blocks. Plotted below these value signals are time courses of the corresponding action-value-prediction error (AVPE) $\delta^Q{}_{t+1}$ signals, which represent a distinct type of reward-prediction error (RPE) along with the state-value-prediction error (SVPE) $\delta^V{}_{t+1}$ (cf. Colas et al., 2017, 2022). However, throughout this report, the usage of the generic term "RPE" and its variable "δ_{t+1}" with no superscript—rather than "AVPE" and "$\delta^Q{}_{t+1}$"—is due to omission of the neural model's SVPE here. Discriminative state and action generalization are evident with counterfactual updates of values for the three nonexperienced state-action pairs within a category. These additional updates occur despite only one state-action pair being experienced with feedback. Each colored tick mark denotes an occurrence of the respective action. This figure corresponds to Figure 7a of the original report (Colas et al., 2022).

		RL		GRL		Bias	Hysteresis								
Model	df	α	τ	g_1	g_2	β_R	λ_H	β_1	β_2	β_3	β_4	β_5	β_6	β_7	β_8
X	0	-	-	-	-	-	-	-	-	-	-	-	-	-	-
XC	1	-	-	-	-	β_R	-	-	-	-	-	-	-	-	-
XN1	1	-	-	-	-	-	-	β_1	-	-	-	-	-	-	-
XN2	2	-	-	-	-	-	-	β_1	β_2	-	-	-	-	-	-
XN3	3	-	-	-	-	-	-	β_1	β_2	β_3	-	-	-	-	-
XN4	4	-	-	-	-	-	-	β_1	β_2	β_3	β_4	-	-	-	-
XN5	5	-	-	-	-	-	-	β_1	β_2	β_3	β_4	β_5	-	-	-
XN6	6	-	-	-	-	-	-	β_1	β_2	β_3	β_4	β_5	β_6	-	-
XN7	7	-	-	-	-	-	-	β_1	β_2	β_3	β_4	β_5	β_6	β_7	-
XN8	8	-	-	-	-	-	-	β_1	β_2	β_3	β_4	β_5	β_6	β_7	β_8
XCN1	2	-	-	-	-	β_R	-	β_1	-	-	-	-	-	-	-
XCN2	3	-	-	-	-	β_R	-	β_1	β_2	-	-	-	-	-	-
XCN3	4	-	-	-	-	β_R	-	β_1	β_2	β_3	-	-	-	-	-
XCN4	5	-	-	-	-	β_R	-	β_1	β_2	β_3	β_4	-	-	-	-
XCN5	6	-	-	-	-	β_R	-	β_1	β_2	β_3	β_4	β_5	-	-	-
XCN6	7	-	-	-	-	β_R	-	β_1	β_2	β_3	β_4	β_5	β_6	-	-
XCN7	8	-	-	-	-	β_R	-	β_1	β_2	β_3	β_4	β_5	β_6	β_7	-
XCN8	9	-	-	-	-	β_R	-	β_1	β_2	β_3	β_4	β_5	β_6	β_7	β_8
XE1	2	-	-	-	-	-	λ_H	β_1	-	-	-	-	-	-	-
XE2	3	-	-	-	-	-	λ_H	β_1	β_2	-	-	-	-	-	-
XE3	4	-	-	-	-	-	λ_H	β_1	β_2	β_3	-	-	-	-	-
XCE1	3	-	-	-	-	β_R	λ_H	β_1	-	-	-	-	-	-	-
XCE2	4	-	-	-	-	β_R	λ_H	β_1	β_2	-	-	-	-	-	-
XCE3	5	-	-	-	-	β_R	λ_H	β_1	β_2	β_3	-	-	-	-	-

0	2	α	τ	-	-	-	-	-	-	-	-	-	-	-	-
0C	3	α	τ	-	-	β_R	-	-	-	-	-	-	-	-	-
0N1	3	α	τ	-	-	-	-	β_1	-	-	-	-	-	-	-
0N2	4	α	τ	-	-	-	-	β_1	β_2	-	-	-	-	-	-
0N3	5	α	τ	-	-	-	-	β_1	β_2	β_3	-	-	-	-	-
0N4	6	α	τ	-	-	-	-	β_1	β_2	β_3	β_4	-	-	-	-
0CN1	4	α	τ	-	-	β_R	-	β_1	-	-	-	-	-	-	-
0CN2	5	α	τ	-	-	β_R	-	β_1	β_2	-	-	-	-	-	-
0CN3	6	α	τ	-	-	β_R	-	β_1	β_2	β_3	-	-	-	-	-
0CN4	7	α	τ	-	-	β_R	-	β_1	β_2	β_3	β_4	-	-	-	-
0E1	4	α	τ	-	-	-	λ_H	β_1	-	-	-	-	-	-	-
0E2	5	α	τ	-	-	-	λ_H	β_1	β_2	-	-	-	-	-	-
0E3	6	α	τ	-	-	-	λ_H	β_1	β_2	β_3	-	-	-	-	-
0CE1	5	α	τ	-	-	β_R	λ_H	β_1	-	-	-	-	-	-	-
0CE2	6	α	τ	-	-	β_R	λ_H	β_1	β_2	-	-	-	-	-	-
0CE3	7	α	τ	-	-	β_R	λ_H	β_1	β_2	β_3	-	-	-	-	-
1	3	α	τ	g_1	-	-	-	-	-	-	-	-	-	-	-
1C	4	α	τ	g_1	-	β_R	-	-	-	-	-	-	-	-	-
1N1	4	α	τ	g_1	-	-	-	β_1	-	-	-	-	-	-	-
1N2	5	α	τ	g_1	-	-	-	β_1	β_2	-	-	-	-	-	-
1N3	6	α	τ	g_1	-	-	-	β_1	β_2	β_3	-	-	-	-	-
1N4	7	α	τ	g_1	-	-	-	β_1	β_2	β_3	β_4	-	-	-	-
1CN1	5	α	τ	g_1	-	β_R	-	β_1	-	-	-	-	-	-	-
1CN2	6	α	τ	g_1	-	β_R	-	β_1	β_2	-	-	-	-	-	-
1CN3	7	α	τ	g_1	-	β_R	-	β_1	β_2	β_3	-	-	-	-	-
1CN4	8	α	τ	g_1	-	β_R	-	β_1	β_2	β_3	β_4	-	-	-	-

1E1	5	α	τ	g_1	-	-	λ_H	β_1	-	-	-	-	-	-	-
1E2	6	α	τ	g_1	-	-	λ_H	β_1	β_2	-	-	-	-	-	-
1E3	7	α	τ	g_1	-	-	λ_H	β_1	β_2	β_3	-	-	-	-	-
1CE1	6	α	τ	g_1	-	β_R	λ_H	β_1	-	-	-	-	-	-	-
1CE2	7	α	τ	g_1	-	β_R	λ_H	β_1	β_2	-	-	-	-	-	-
1CE3	8	α	τ	g_1	-	β_R	λ_H	β_1	β_2	β_3	-	-	-	-	-
2	4	α	τ	g_1	g_2	-	-	-	-	-	-	-	-	-	-
2C	5	α	τ	g_1	g_2	β_R	-	-	-	-	-	-	-	-	-
2N1	5	α	τ	g_1	g_2	-	-	β_1	-	-	-	-	-	-	-
2N2	6	α	τ	g_1	g_2	-	-	β_1	β_2	-	-	-	-	-	-
2N3	7	α	τ	g_1	g_2	-	-	β_1	β_2	β_3	-	-	-	-	-
2N4	8	α	τ	g_1	g_2	-	-	β_1	β_2	β_3	β_4	-	-	-	-
2CN1	6	α	τ	g_1	g_2	β_R	-	β_1	-	-	-	-	-	-	-
2CN2	7	α	τ	g_1	g_2	β_R	-	β_1	β_2	-	-	-	-	-	-
2CN3	8	α	τ	g_1	g_2	β_R	-	β_1	β_2	β_3	-	-	-	-	-
2CN4	9	α	τ	g_1	g_2	β_R	-	β_1	β_2	β_3	β_4	-	-	-	-
2E1	6	α	τ	g_1	g_2	-	λ_H	β_1	-	-	-	-	-	-	-
2E2	7	α	τ	g_1	g_2	-	λ_H	β_1	β_2	-	-	-	-	-	-
2E3	8	α	τ	g_1	g_2	-	λ_H	β_1	β_2	β_3	-	-	-	-	-
2CE1	7	α	τ	g_1	g_2	β_R	λ_H	β_1	-	-	-	-	-	-	-
2CE2	8	α	τ	g_1	g_2	β_R	λ_H	β_1	β_2	-	-	-	-	-	-
2CE3	9	α	τ	g_1	g_2	β_R	λ_H	β_1	β_2	β_3	-	-	-	-	-

Table A. Model parameters (unrolled). See Table 2. Models are listed individually here.

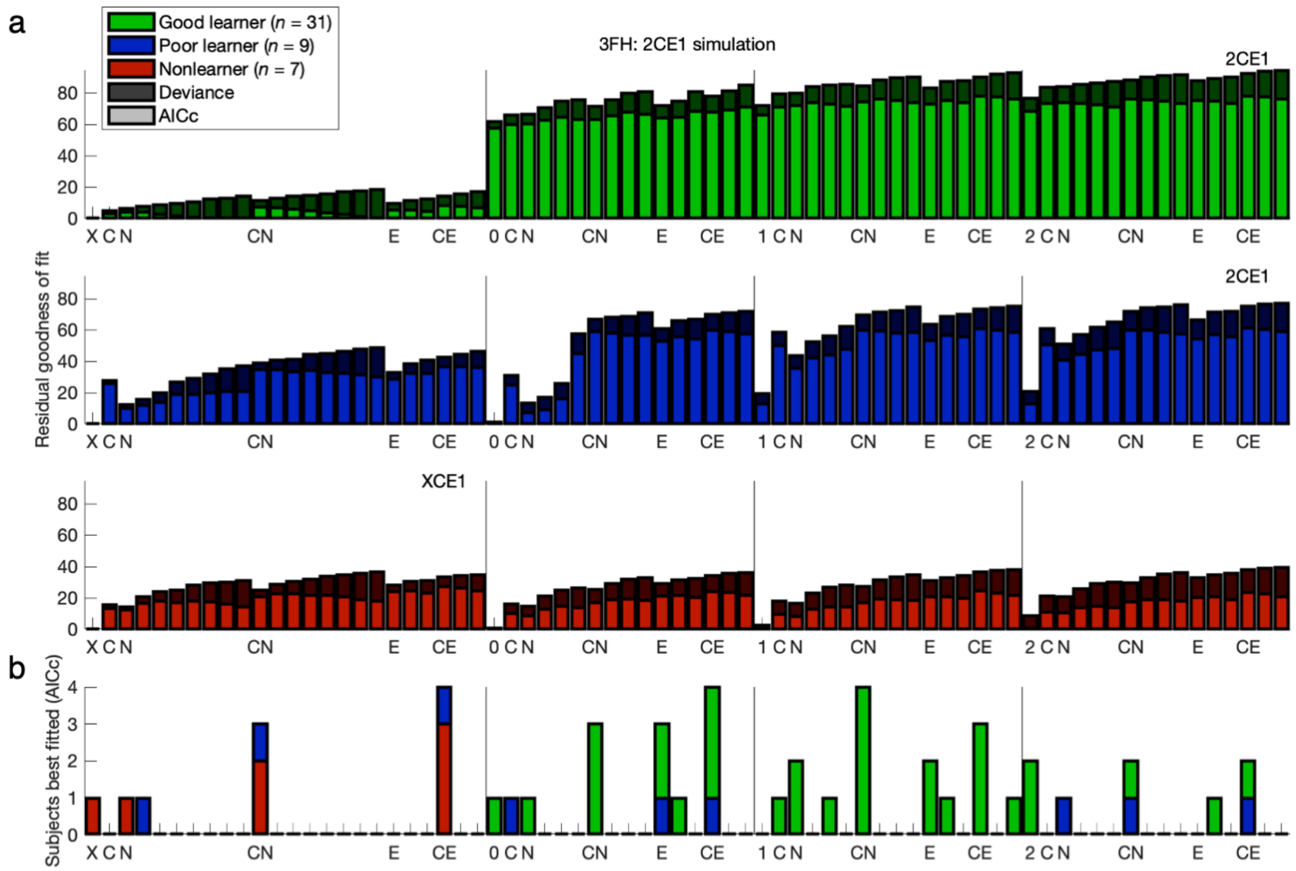

Fig C. Discriminability of the 2CE1 model: 3-T Face/House version. Compare to Fig 2. Each fitted instantiation of the preferred 2CE1 model was used to simulate a data set yoked to that of the respective participant. The results from the empirical model comparison were replicated in silico as a demonstration of the discriminability of this 7-parameter model among both simpler and more complex alternatives ranging from 0 to 9 free parameters. Model recovery succeeded inasmuch as the 2CE1 model remained preferred among Good learners, and 2CE1 or its nonlearning analog XCE1 could be recovered for Poor learners or Nonlearners as well. See also Tables G, H, and I.

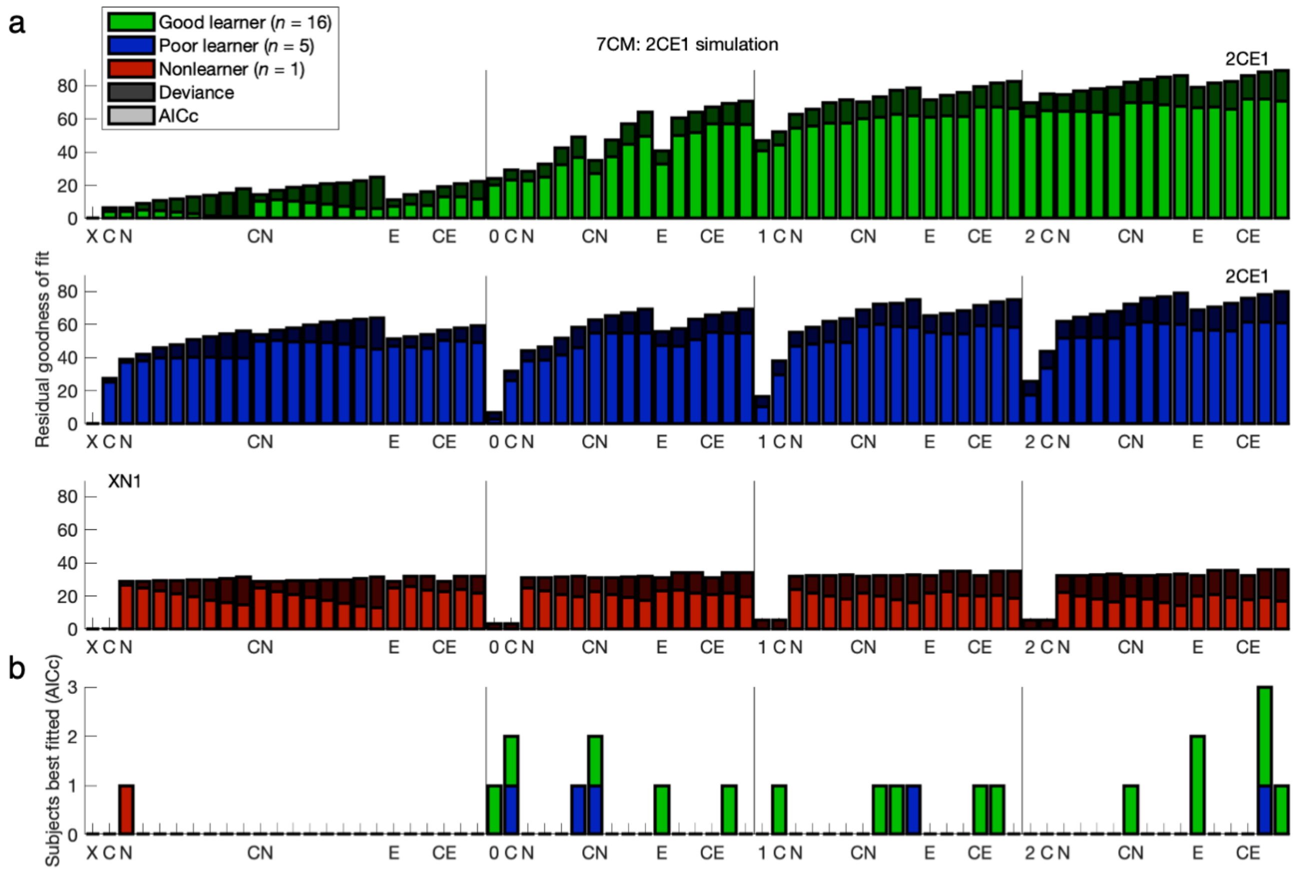

Fig D. Discriminability of the 2CE1 model: 7-T Color/Motion version. Compare to Fig 3 and Fig C. See also Tables J and K.

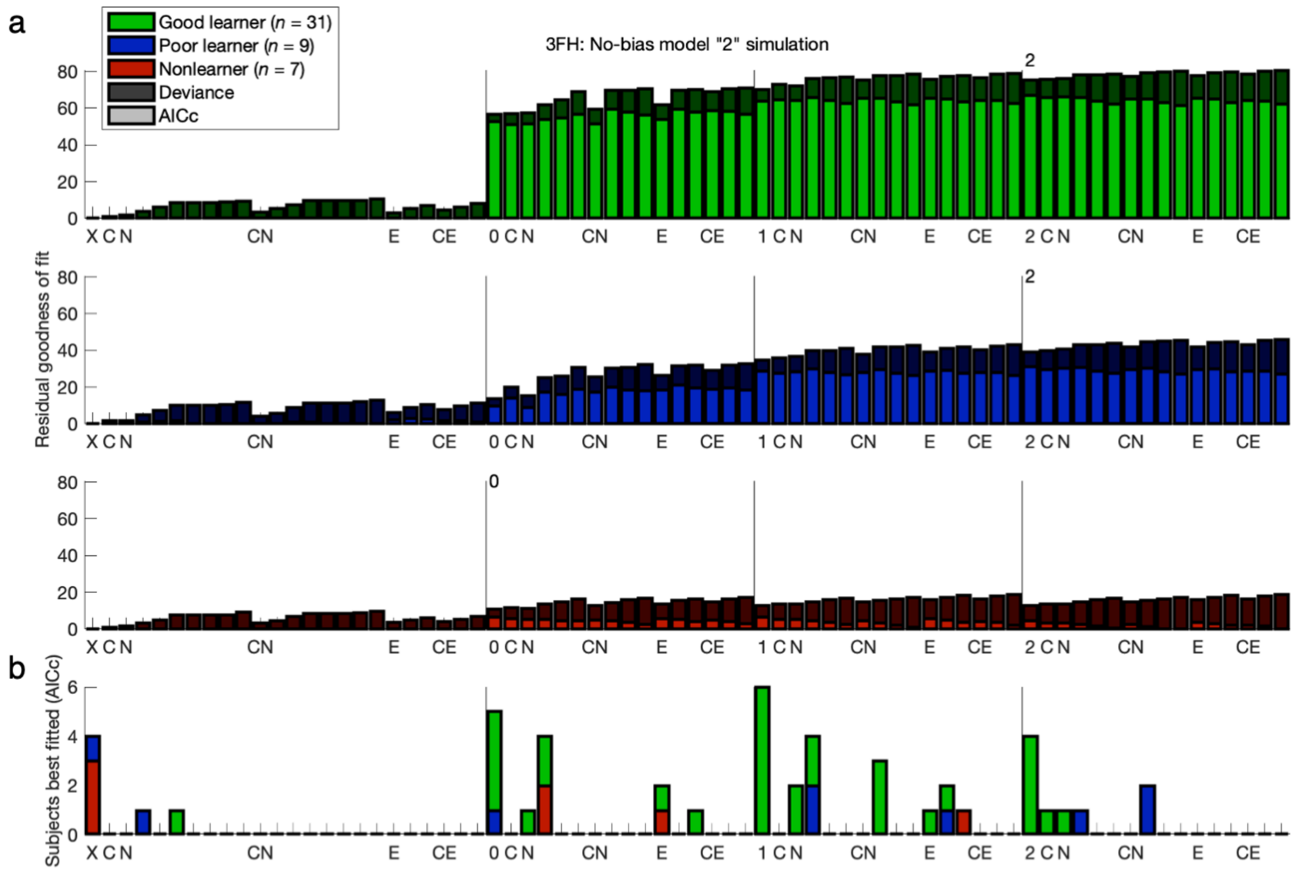

Fig E. Discriminability of the no-bias model "2" with only GRL: 3-T Face/House version. Compare to Fig C. The no-bias model "2" was recovered in lieu of the bias-and-hysteresis model 2CE1 when substituting data simulated with the no-bias model. This converse model recovery again demonstrates an absence of overfitting. See also Tables L, M, and N.

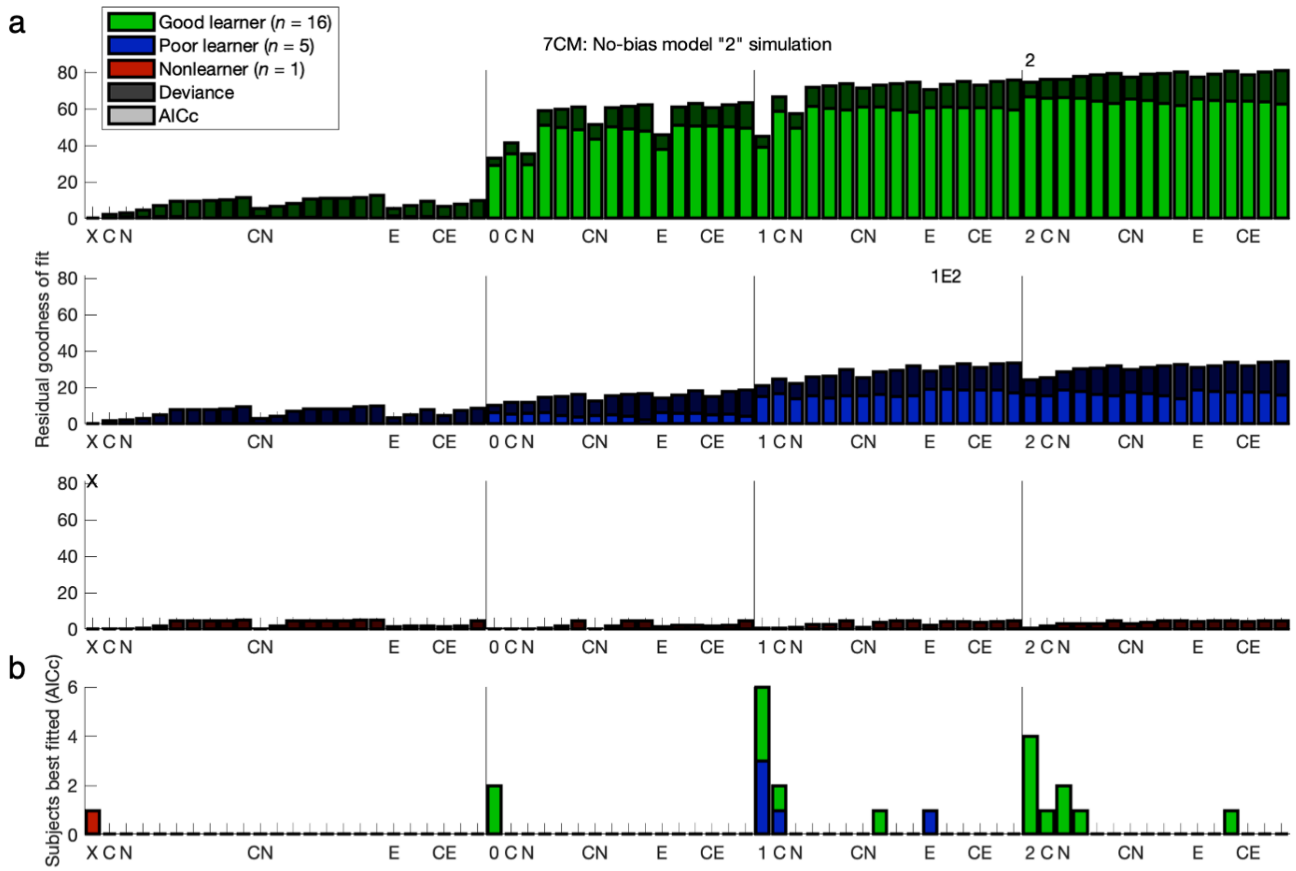

Fig F. Discriminability of the no-bias model "2" with only GRL: 7-T Color/Motion version. Compare to Figs D and E. See also Tables O and P.

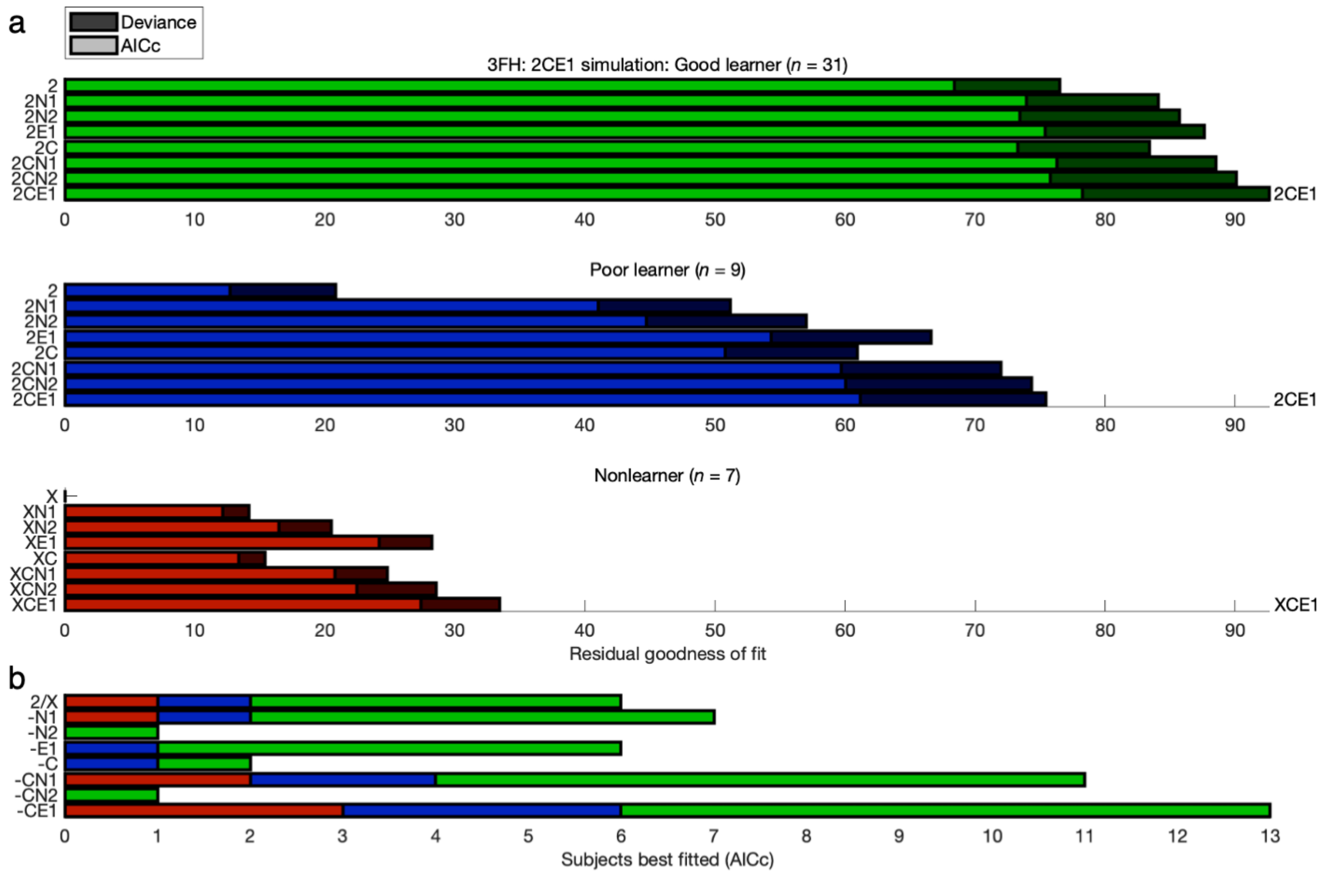

Fig G. Reduced model comparison for discriminability of the 2CE1 model: 3-T Face/House version. Compare to Fig 4 and Fig C.

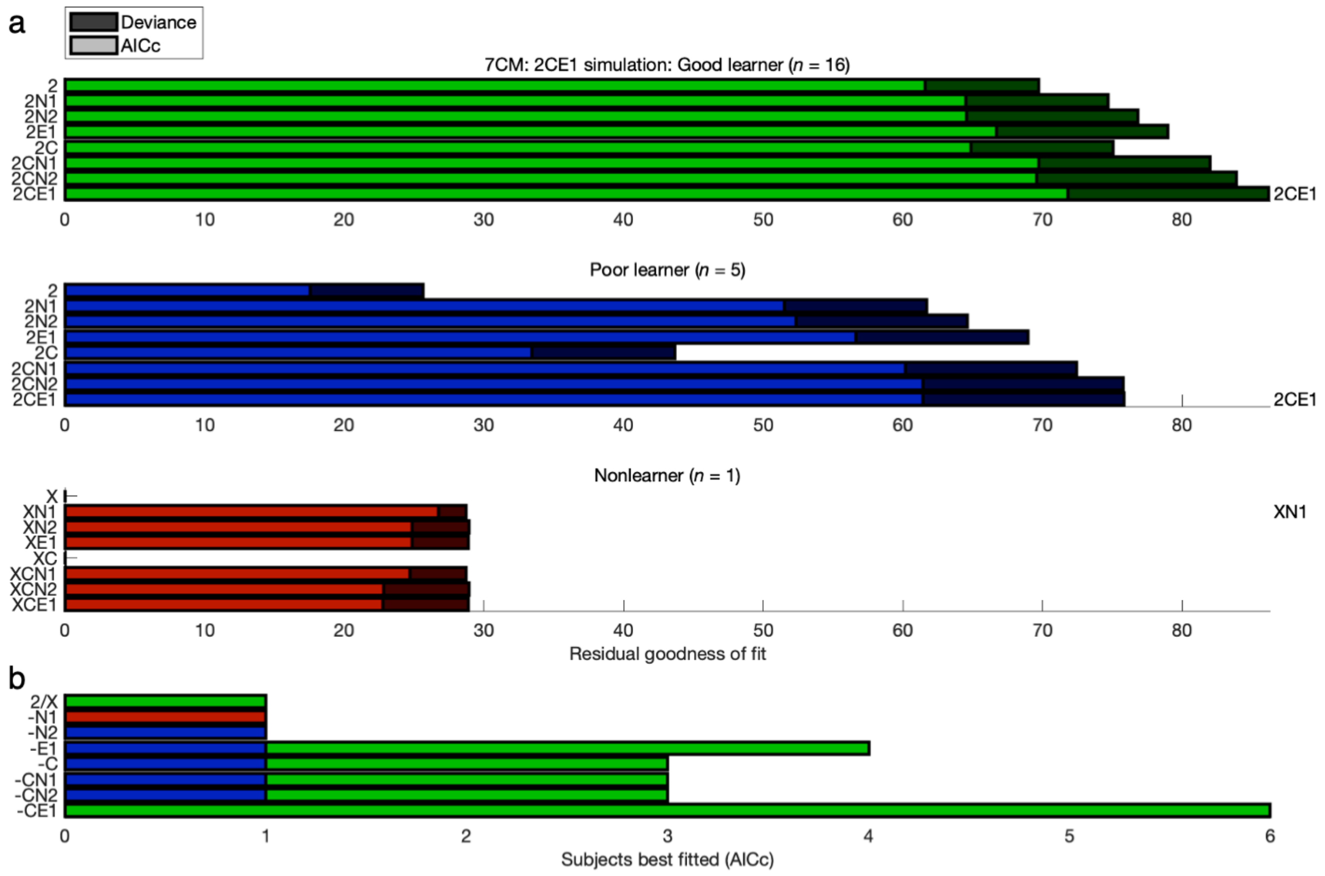

Fig H. Reduced model comparison for discriminability of the 2CE1 model: 7-T Color/Motion version. Compare to Fig 5 and Figs D and G.

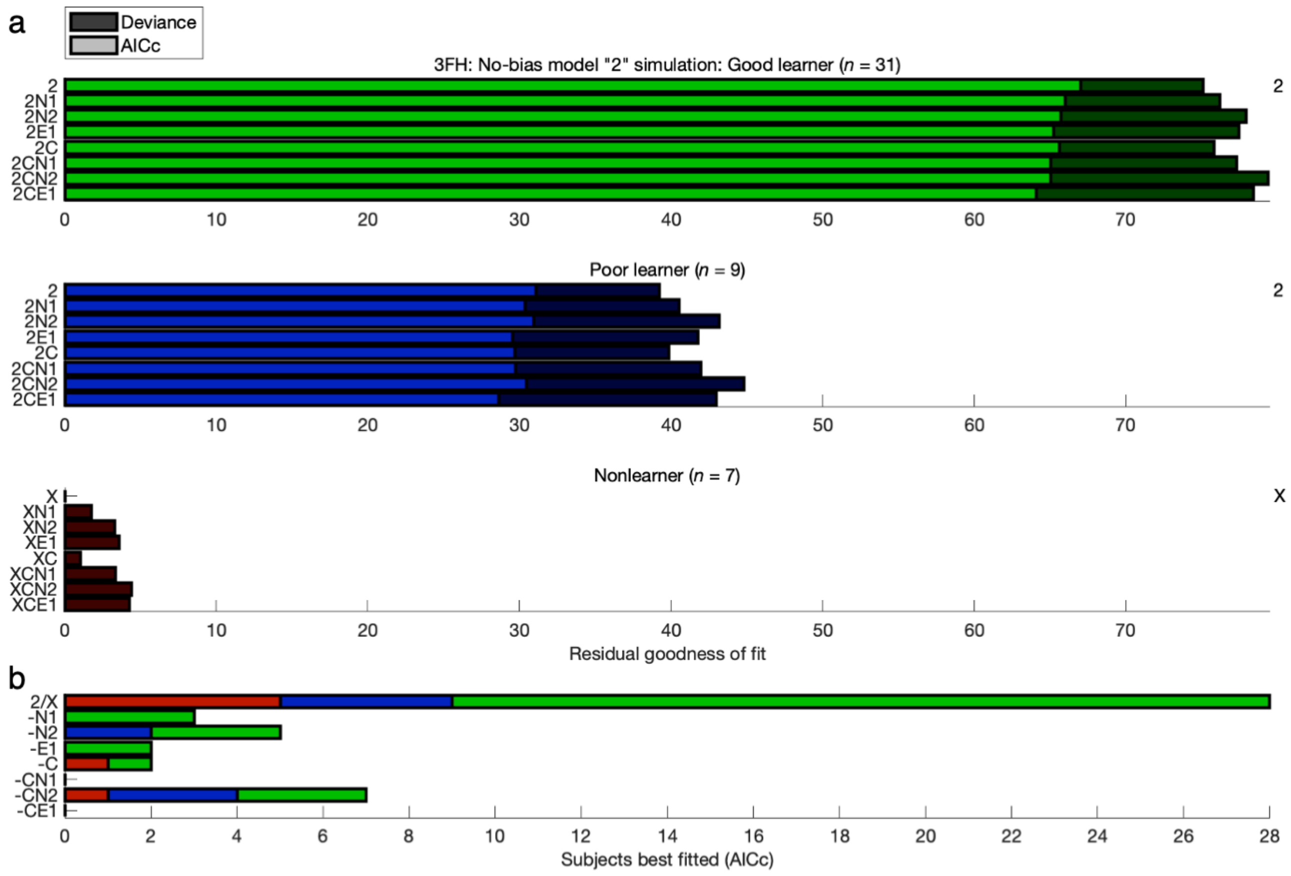

Fig I. Reduced model comparison for discriminability of the no-bias model "2" with only GRL: 3-T Face/House version. Compare to Fig E.

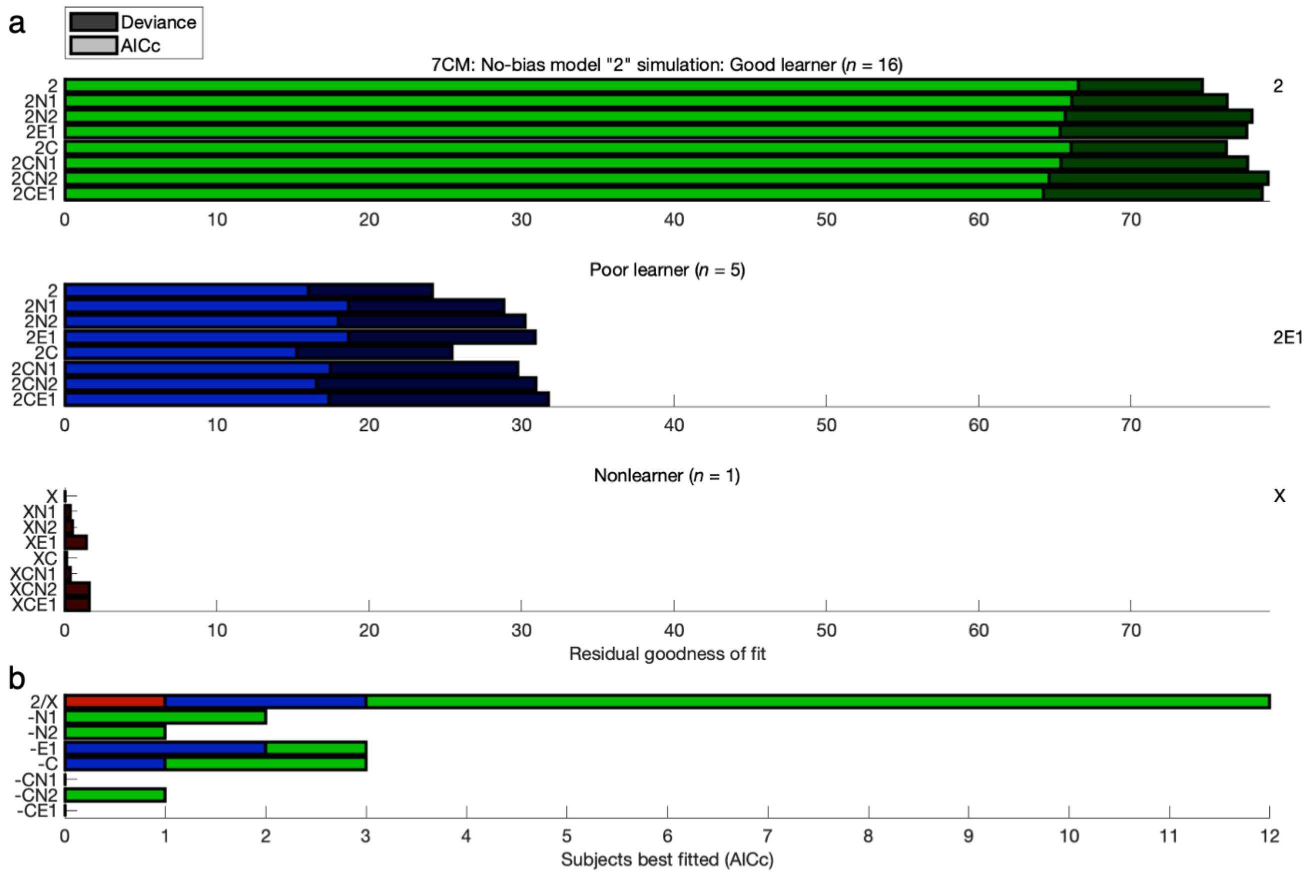

Fig J. Reduced model comparison for discriminability of the no-bias model "2" with only GRL: 7-T Color/Motion version. Compare to Figs F and I.

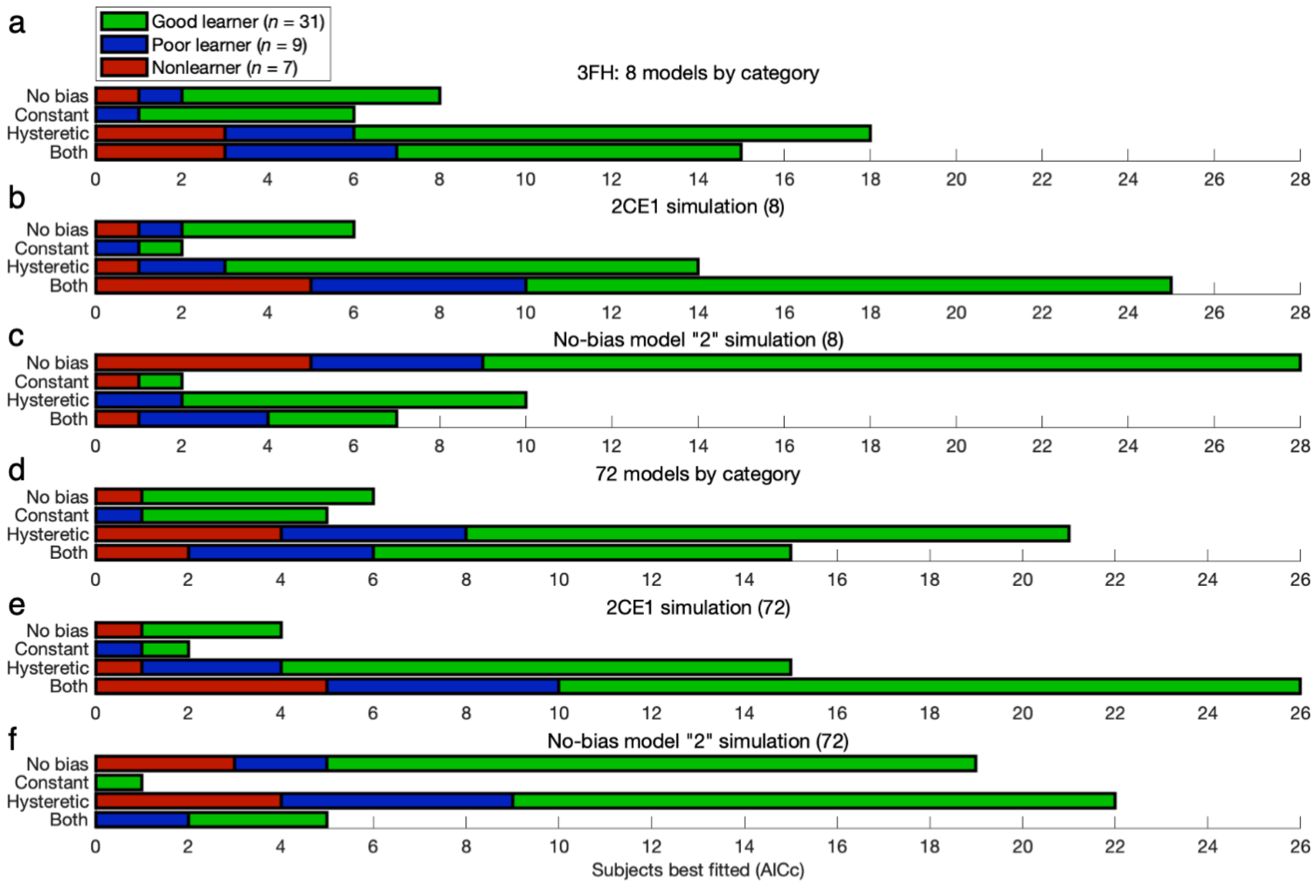

Fig K. Model comparison by bias category: 3-T Face/House version. Compare to Figs 2 (panel d here) and 4 (a) and Figs C (e), E (f), G (b), and I (c). Participant counts for best-fitting models can also be grouped according to four categories: no bias (e.g., “2”), constant bias (e.g., 2C), hysteretic bias (e.g., 2E1), or both constant and hysteretic bias (e.g., 2CE1).

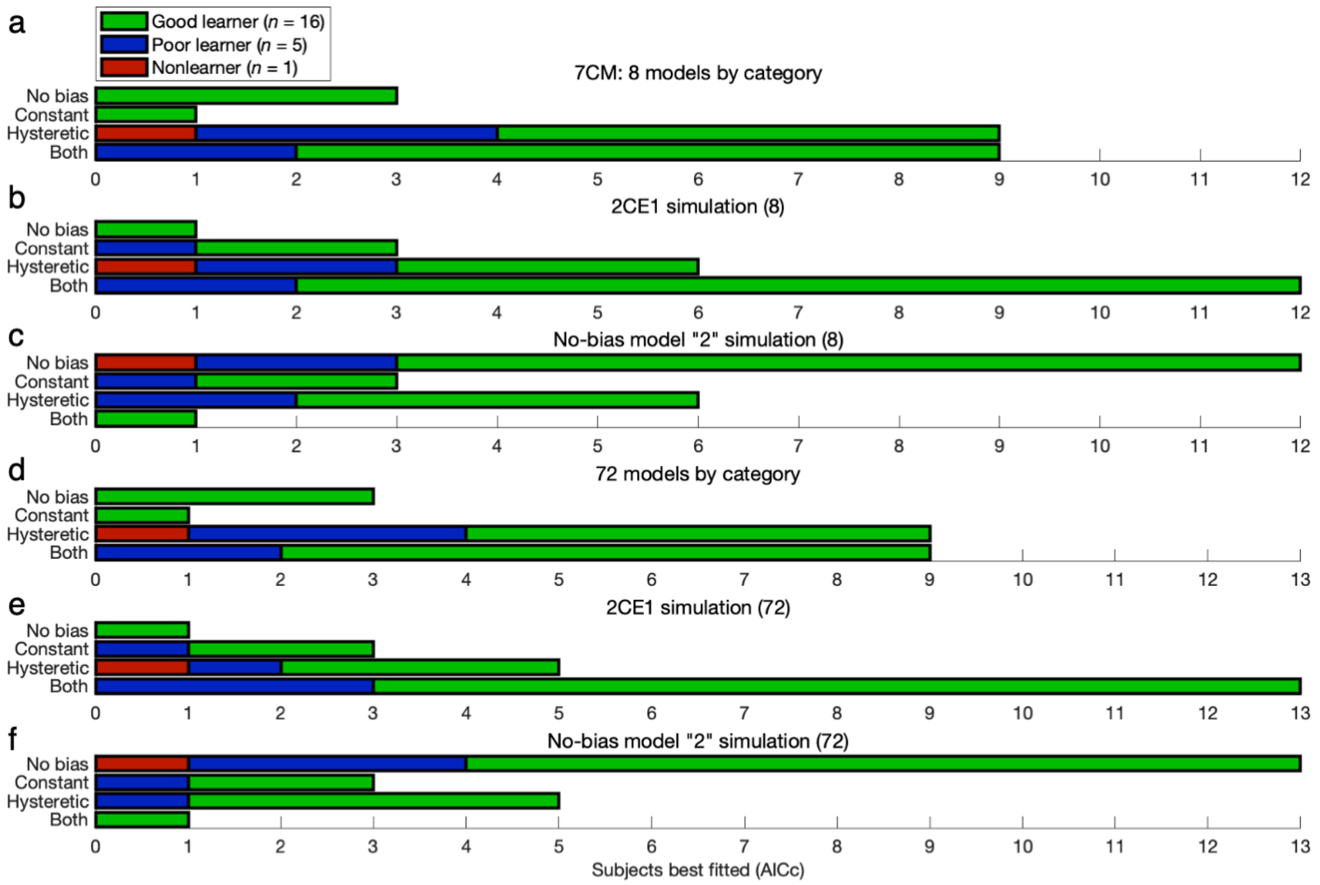

Fig L. Model comparison by bias category: 7-T Color/Motion version. Compare to Figs 3 (panel d here) and 5 (a) and Figs D (e), F (f), H (b), J (c), and K.

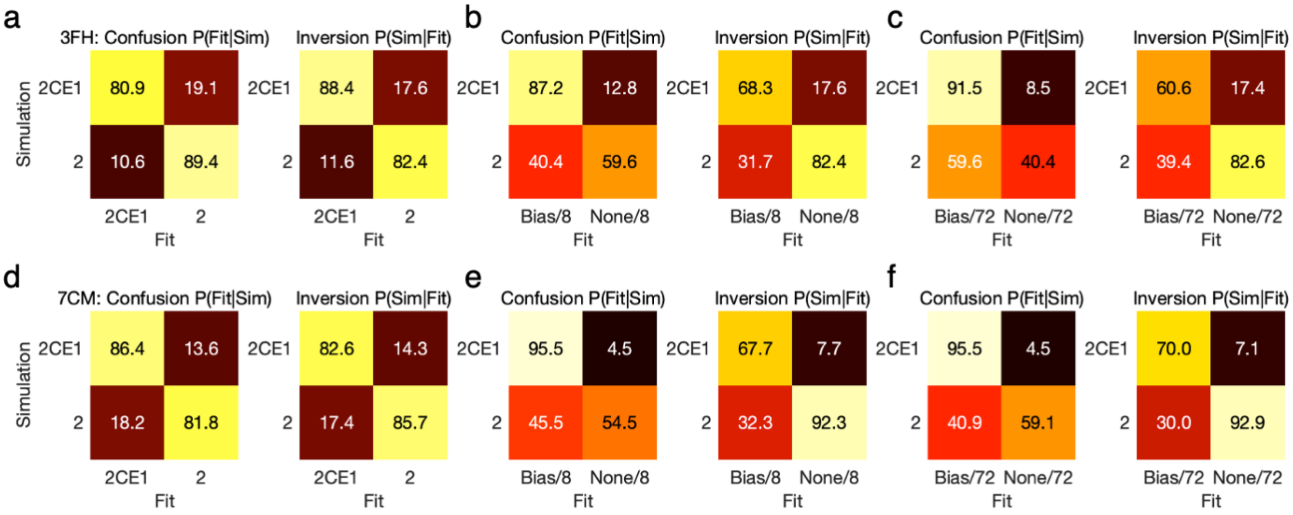

Fig M. Confusion matrix and inverse-confusion matrix. Compare to Figs K and L. The confusion matrix *P(Fit | Simulation)* corresponds to the probability (as a percentage) that simulated data from a given model are best fitted by either the model that actually generated the data or an alternative model. The inversion matrix *P(Simulation | Fit)* instead corresponds to the probability that a model generated the simulated data given that either the same model or an alternative model fitted the data best. **(a)** Limiting the model comparison to only the 2CE1 or "2" models with or without bias and hysteresis, model confusion is minimal as expected. **(b-c)** Expanding the model comparison with a binarized categorization of bias versus none for either 8 (b) or 72 (c) models does leave confusion less minimal as the models with bias outnumber the models without bias, but the expected trend of model recovery still holds true. **(d-f)** Results were replicated in the 7-T Color/Motion version of the experiment.

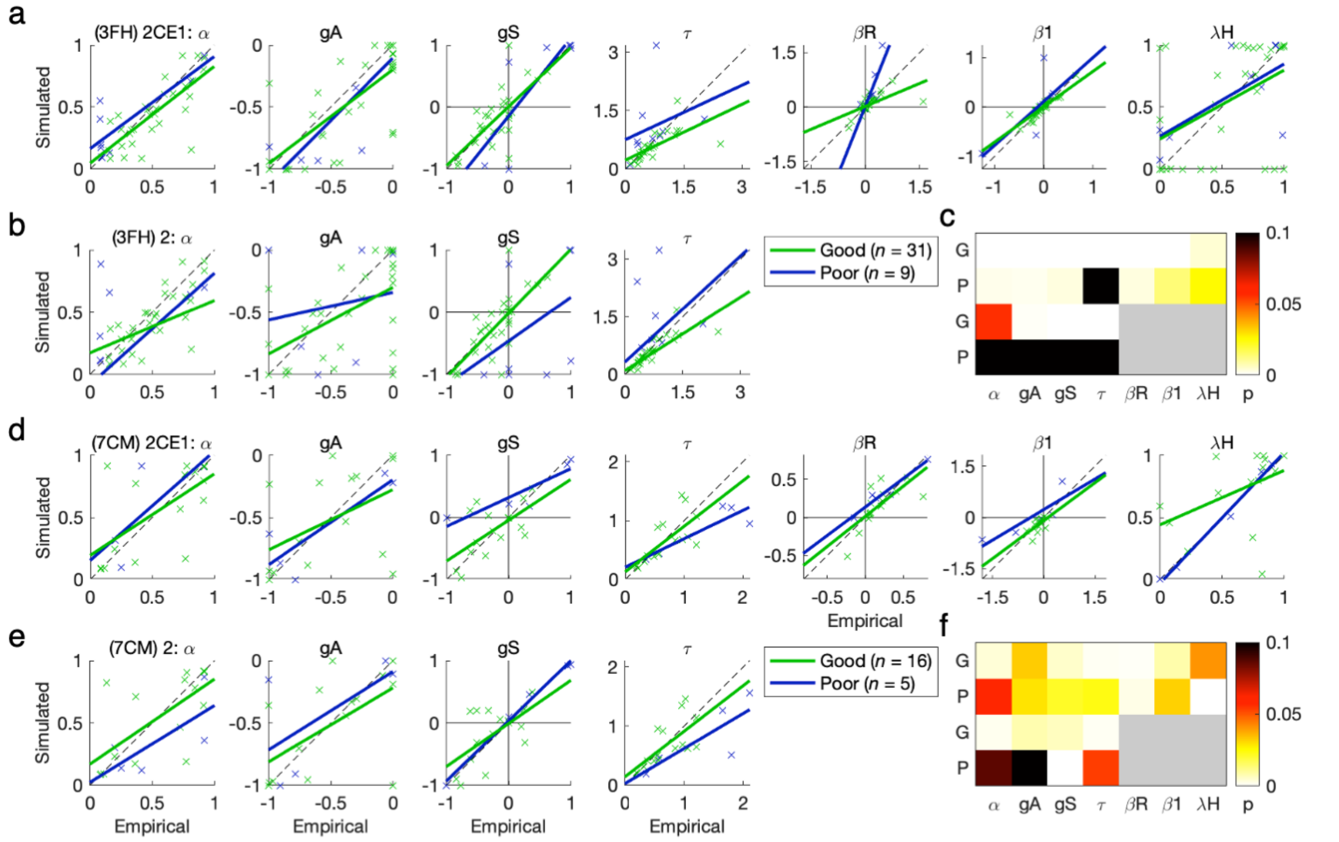

Fig N. Parameter recovery with the 2CE1 model more accurate than recovery with the no-bias model "2" including only GRL. (a) As described previously, the 2CE1 model was fitted to yoked simulated data that were generated with the 2CE1 parameters originally fitted to empirical data. Parameter recovery was especially robust for the Good-learner group across all seven free parameters, including β_R, β_1, and λ_H for action bias and hysteresis ($p < 0.05$). Although somewhat less robust, recovery of 2CE1 parameters was also successful for the Poor-learner group ($p < 0.05$ with the exception of τ from the first data set and $p < 0.06$ for α from the second data set). **(b)** The relative significance of bias and hysteresis was found to be greatest among Poor learners. Hence, if instead fitting the no-bias model "2", the remaining four parameters needed for pure GRL (α, g_A, g_S, τ) were not significantly recoverable for the Poor-learner group ($p > 0.05$ with one exception for g_S from the second data set). **(c)** The p values for the correlations are plotted separately for Good ("G") and Poor ("P") learners when using either the 7-parameter 2CE1 model or the 4-parameter model "2". **(d-f)** Results were replicated in the 7-T Color/Motion version of the experiment.

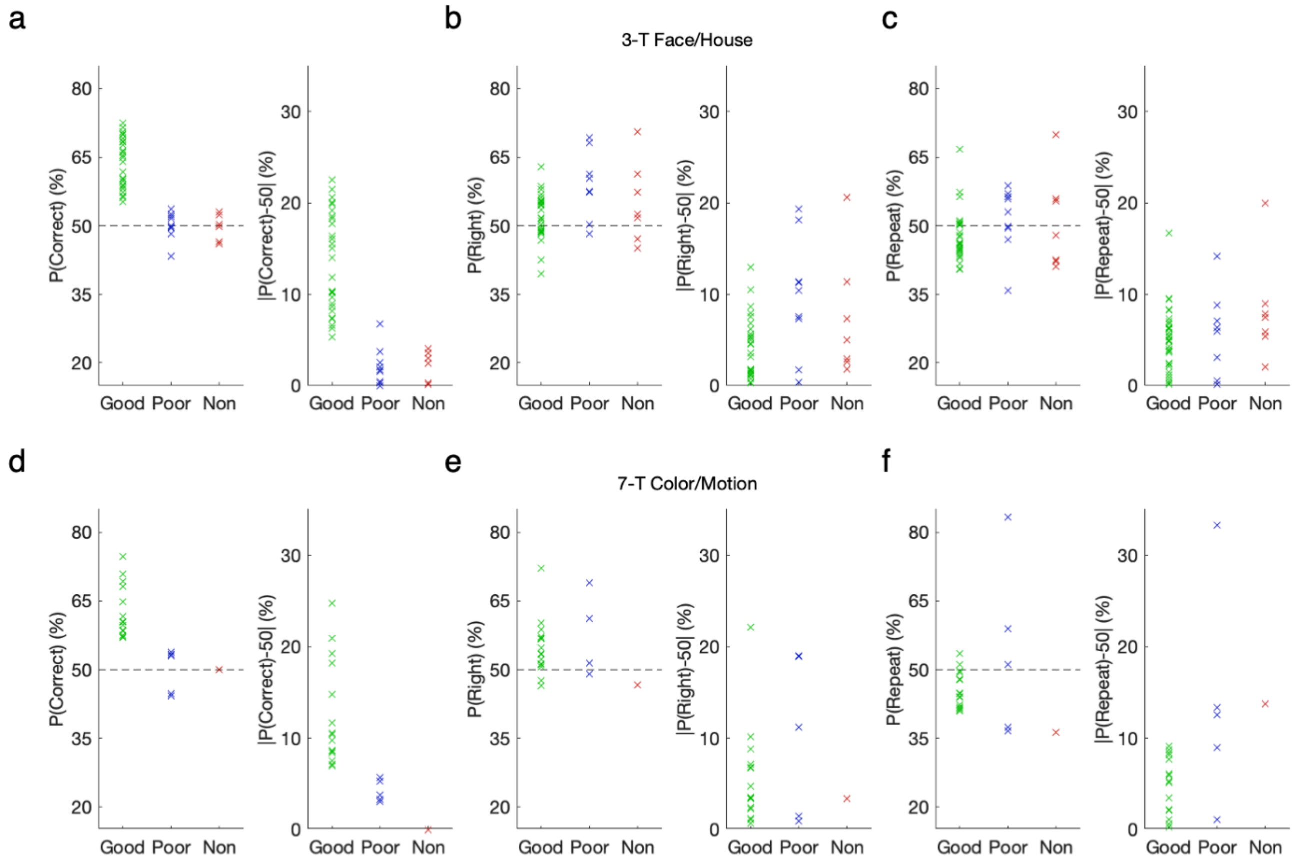

Fig O. Action bias and hysteresis versus learning performance: Individual results. Compare to Figs 6 and 7.

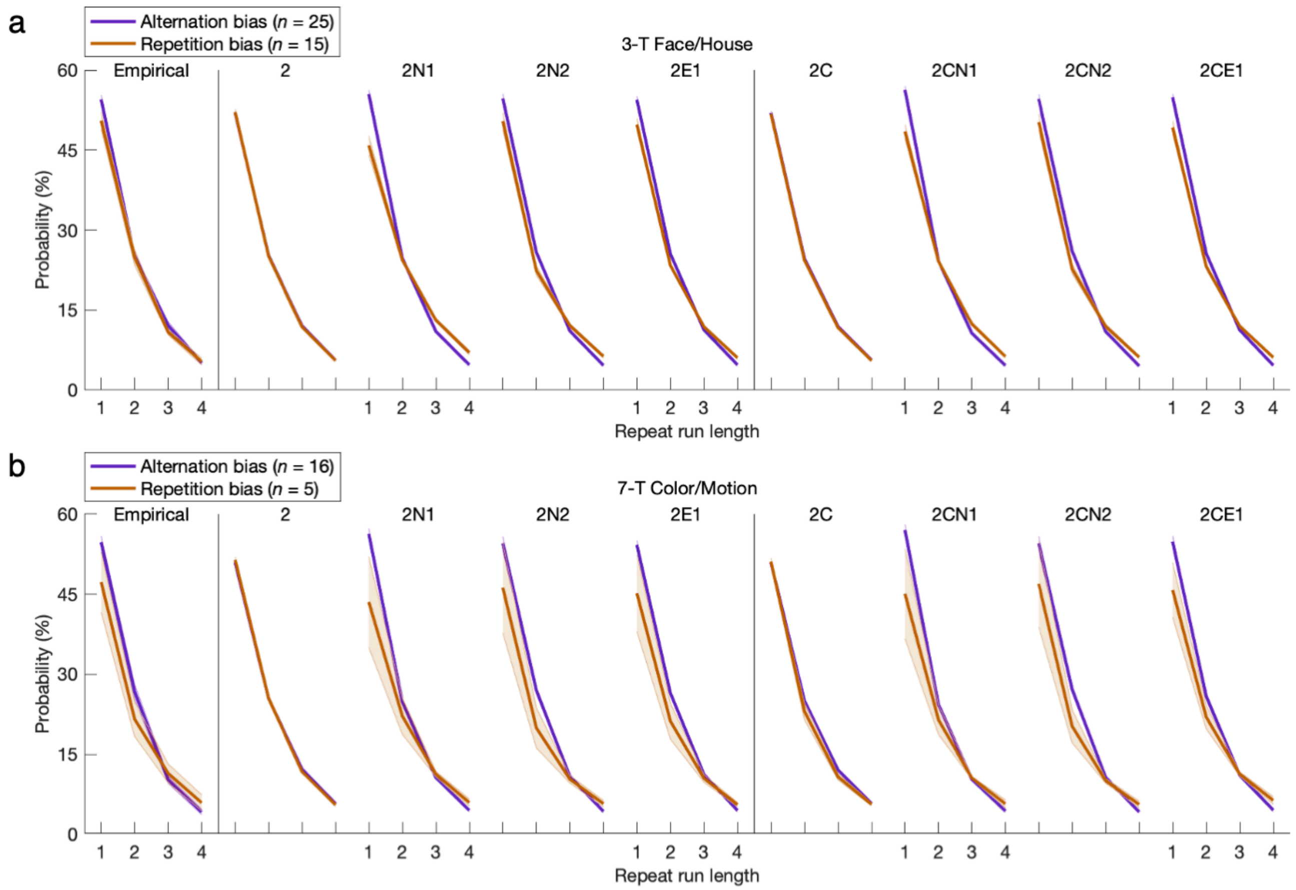

Fig P. Hysteresis represented by sequences across trials. Compare to Fig 12. The distribution of lengths of runs of consecutive repeated actions reveals hysteresis from another perspective. Alternation and repetition biases should result in shorter and longer runs, respectively, as only a model including hysteresis could replicate. Error bars indicate standard errors of the means.

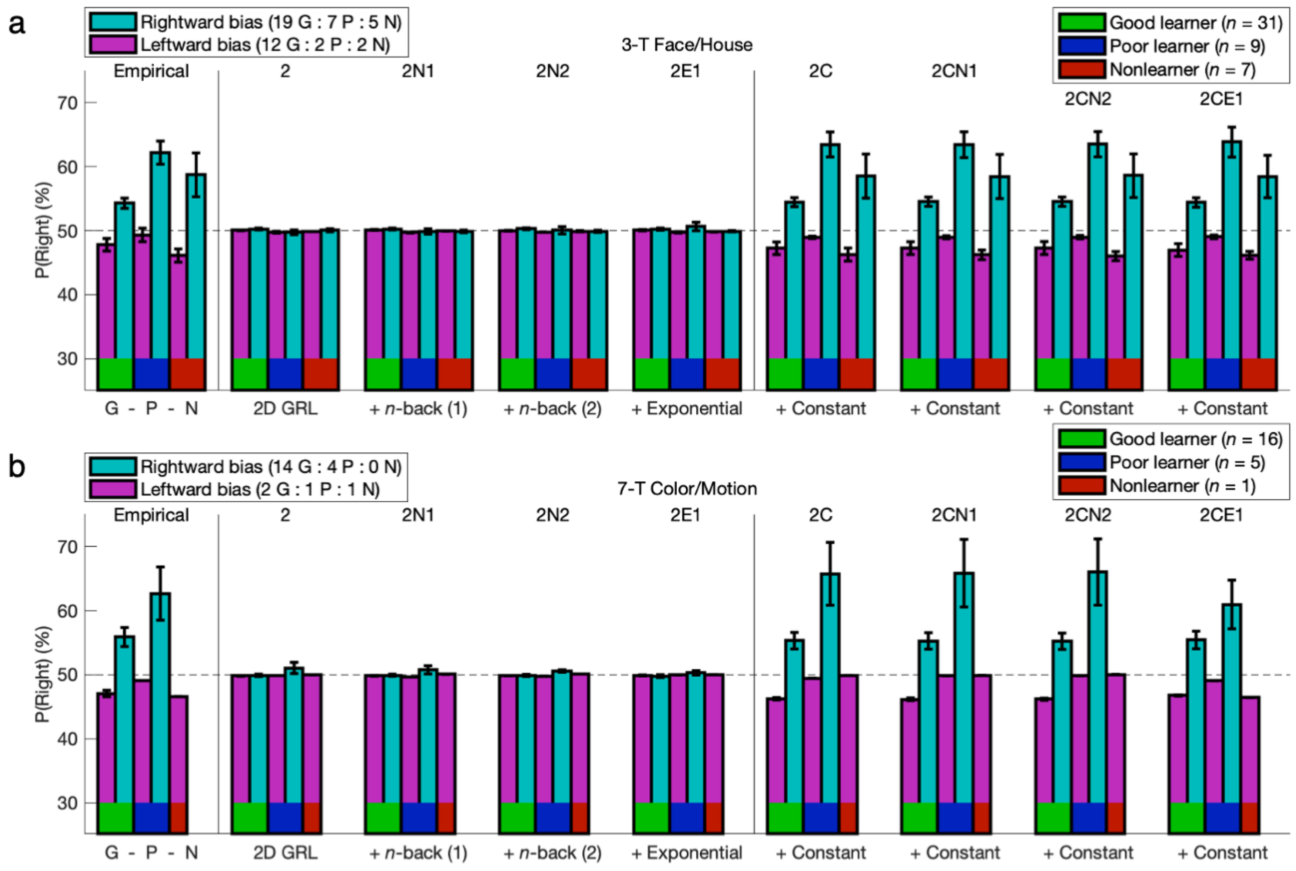

Fig Q. Constant bias and learning performance. Compare to Figs 6, 7, and 8. Participants were further divided into six subgroups that separated the two directions of constant lateral bias as well as the three levels of learning performance. Constant bias should still be substantial for Good learners but should be even more pronounced for Poor learners and Nonlearners. Moreover, modeled bias in 2CE1 simulations should still both qualitatively and quantitively replicate the directions and magnitudes of empirical effects of laterality.

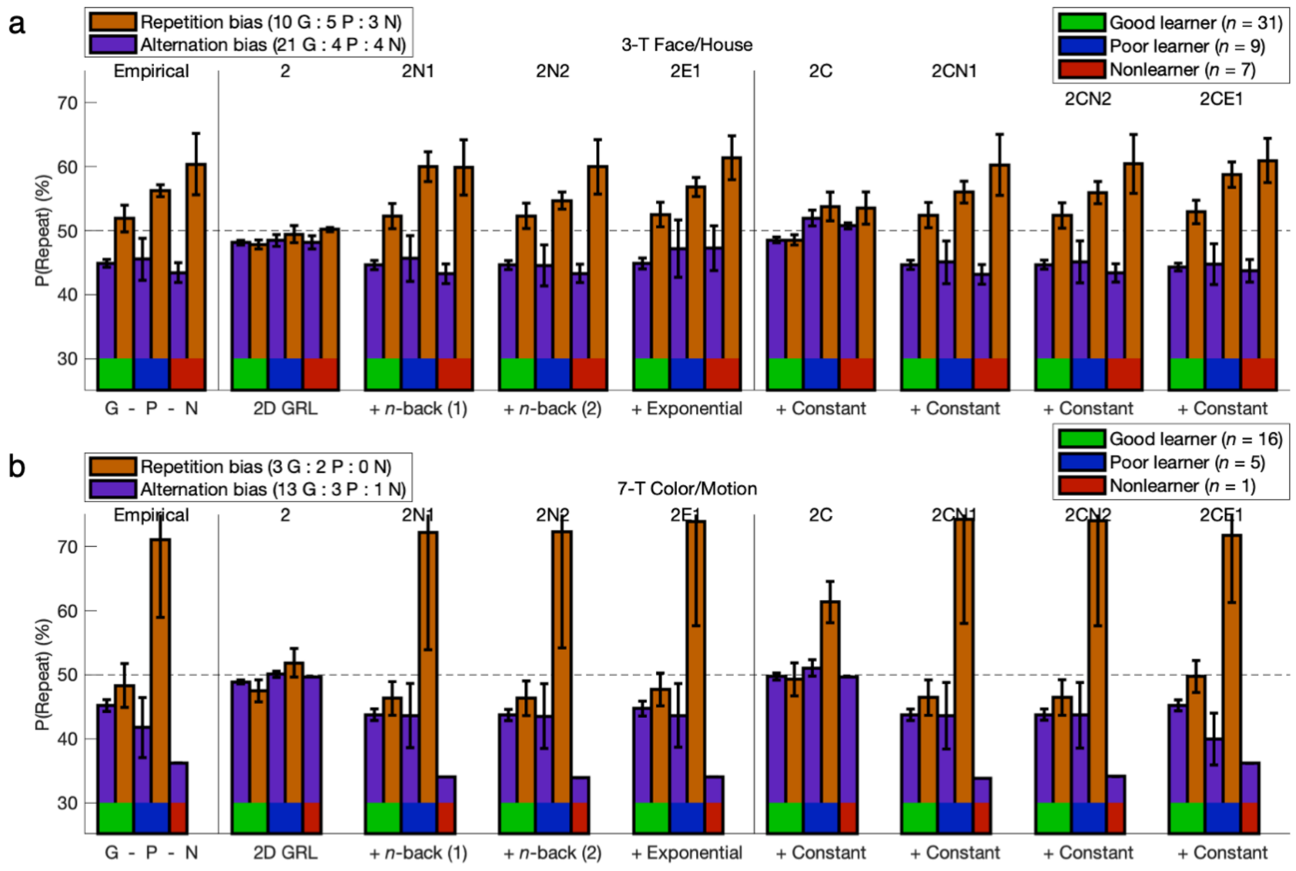

Fig R. Hysteresis and learning performance. Compare to Figs 6, 7, and 9. Participants were next subdivided with the two directions of hysteretic bias as the first factor crossed with learning performance. As with constant bias, hysteretic bias should still be substantial for Good learners but should be even more pronounced for Poor learners and Nonlearners. Likewise, modeled bias in 2CE1 simulations should still replicate the directions and magnitudes of empirical effects of hysteresis.

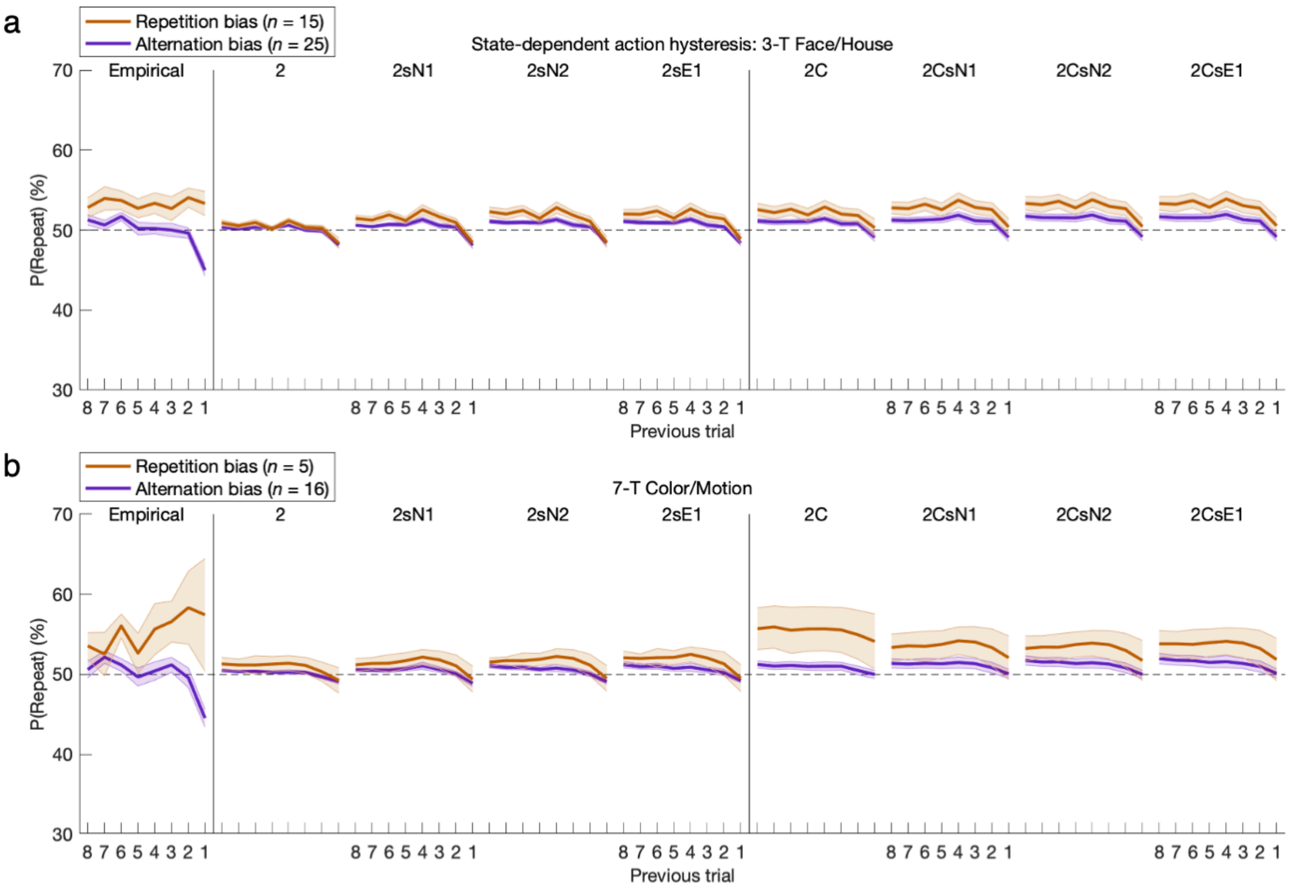

Fig S. Substitution of state-dependent action hysteresis. Compare to Figs 12 and 14. The alternative of state-dependent hysteresis $H_t(s_t,a)$ was first substituted in place of state-independent hysteresis $H_t(a)$. Following the original reduced comparison of eight models, here state-dependent action hysteresis was tested in its 1-back (2CsN1), 2-back (2CsN2) and exponential (2CsE1) forms. As expected because the four states (which this hysteresis depends on) were rotated in sequence, each form of state-dependent hysteresis by itself failed to match the action-history curves here.

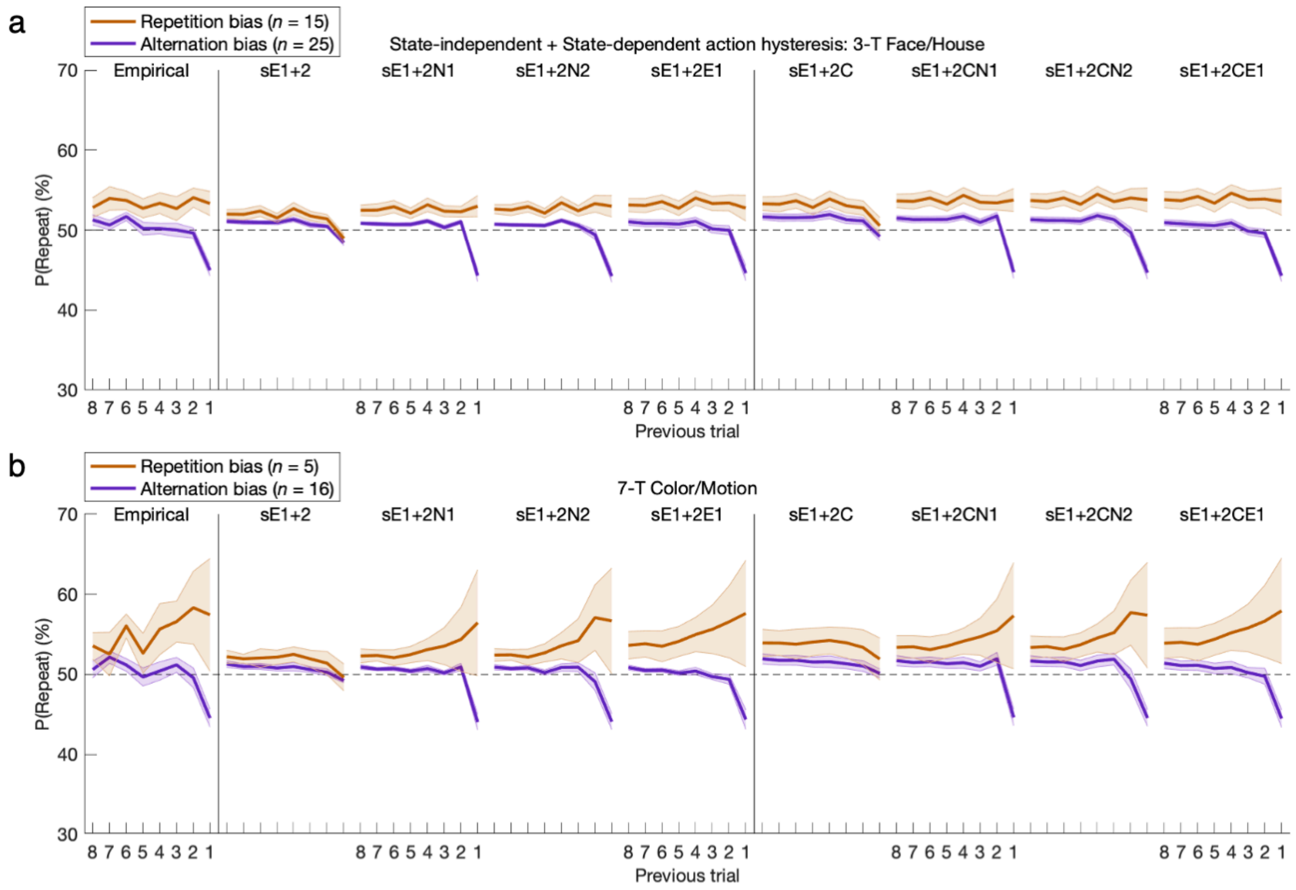

Fig T. Addition of state-dependent action hysteresis to state-independent action hysteresis. Compare to Figs 12 and 14 and Fig S. State-dependent hysteresis $H_t(s_t,a)$ in exponential form ("sE1+") was subsequently added to the eight models from the original reduced model comparison with state-independent hysteresis $H_t(a)$ (2 through 2CE1). Considering that the 2CE1 model in its own right could parsimoniously account for all of these alternation and repetition effects, the expanded sE1+2CE1 model was not justified by any qualitative improvement in fit.

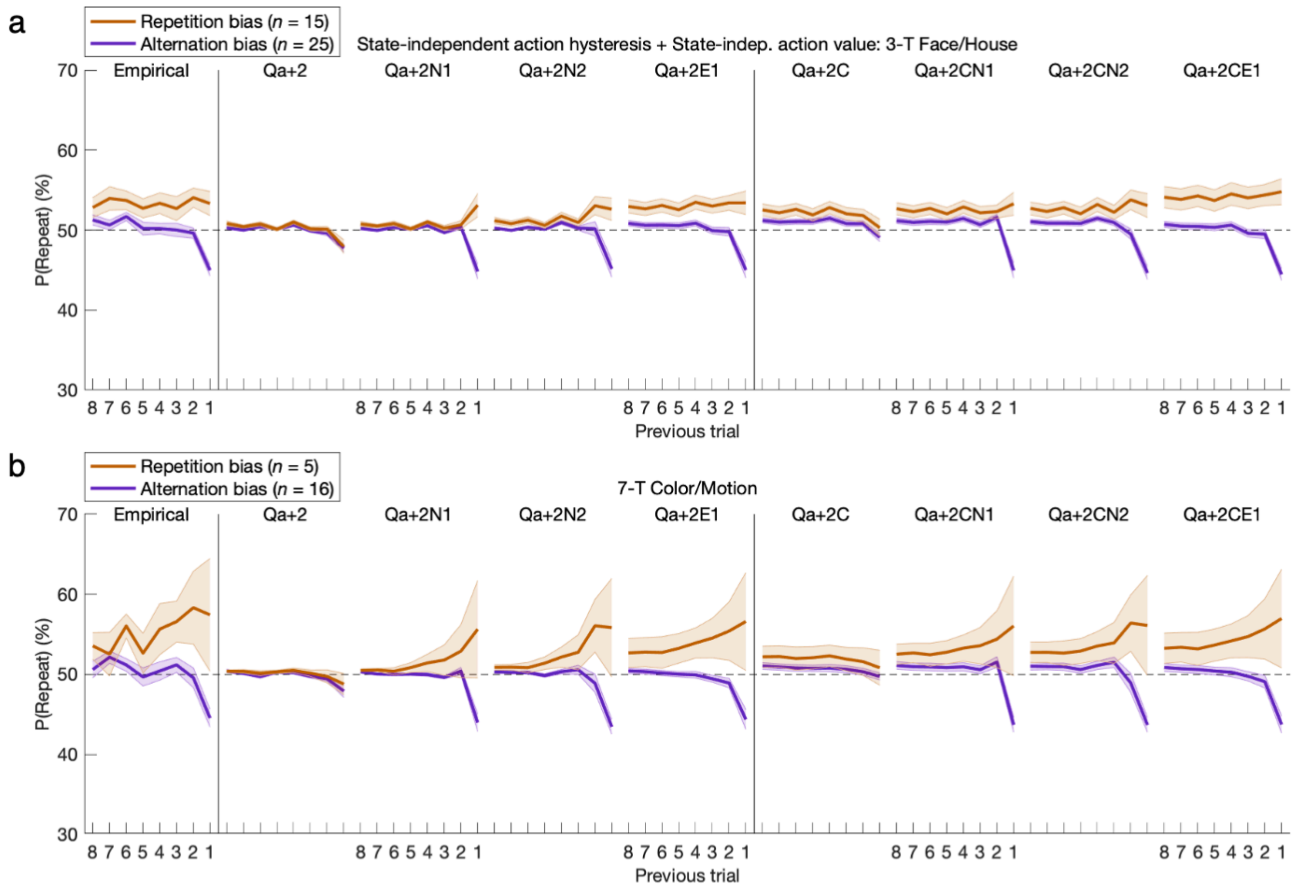

Fig U. Addition of state-independent action value. Compare to Figs 12 and 14. State-independent action value $Q_t(a)$ was added to the eight models from the original reduced model comparison with only state-dependent action value $Q_t(s_t,a)$. Again, the expanded Qa+2CE1 model was not justified by any qualitative improvement in fit.

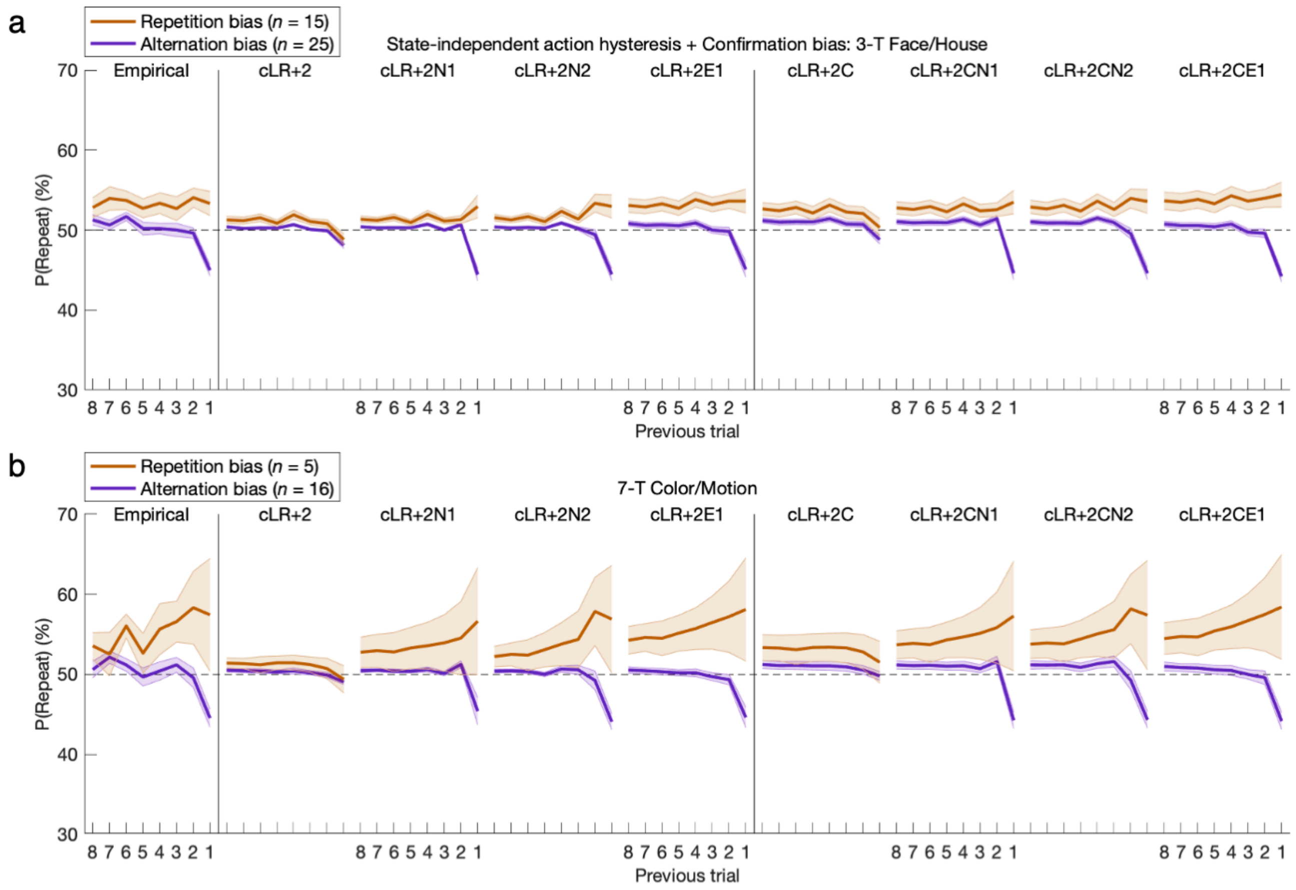

Fig V. Addition of confirmation bias. Compare to Figs 12 and 14. A second learning rate was added to distinguish updates for positive and negative reward-prediction errors (α_P and α_N). Models with confirmation bias ("cLR+") in particular imposed the constraint $\alpha_N < \alpha_P$ with an assumption of subjective optimism biased toward positive valence. The expanded cLR+2CE1 model was not justified by any qualitative improvement in fit.

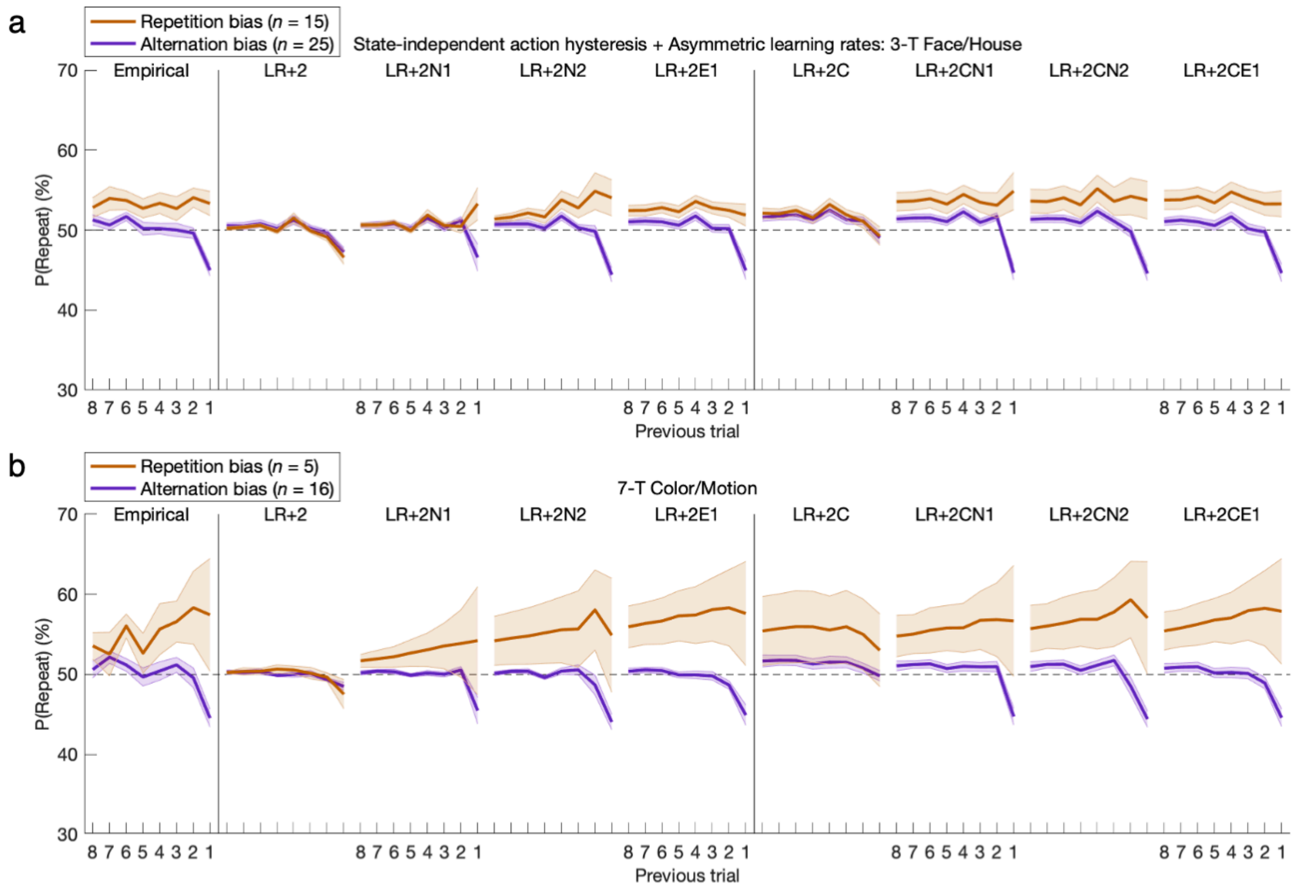

Fig W. Addition of asymmetric learning rates. Compare to Figs 12 and 14 and Fig V. As before, a second learning rate was added to distinguish updates for positive and negative reward-prediction errors, but here the asymmetric learning rates $\alpha_N \neq \alpha_P$ had no constraint of confirmation bias such that pessimistic doubt bias was also a possibility. Even in this unconstrained form, the expanded LR+2CE1 model still was not justified by any qualitative improvement in fit.

3-T Face/House Empirical data		Good learner (n = 31)			
		Absolute		Residual	
Model	df	Dev.	AICc	Dev.	AICc
X	0	501.26	501.26	0	0
XC	1	497.80	499.81	3.45	1.44
XN1	1	496.11	498.12	5.15	3.14
XN2	2	494.75	498.78	6.51	2.47
XN3	3	493.23	499.30	8.02	1.96
XN4	4	492.40	500.51	8.86	0.74
XN5	5	491.10	501.27	10.16	-0.01
XN6	6	489.55	501.80	11.70	-0.54
XN7	7	488.54	502.86	12.72	-1.60
XN8	8	487.60	504.02	13.66	-2.76
XCN1	2	492.35	496.38	8.91	4.88
XCN2	3	490.80	496.87	10.46	4.39
XCN3	4	489.30	497.42	11.95	3.84
XCN4	5	488.58	498.75	12.68	2.50
XCN5	6	487.34	499.58	13.92	1.68
XCN6	7	486.07	500.39	15.19	0.86
XCN7	8	485.07	501.48	16.19	-0.23
XCN8	9	483.98	502.50	17.28	-1.24
XE1	2	493.78	497.81	7.48	3.44
XE2	3	491.43	497.50	9.83	3.76
XE3	4	490.32	498.43	10.94	2.83
XCE1	3	489.80	495.87	11.46	5.39
XCE2	4	487.93	496.05	13.32	5.21
XCE3	5	486.97	497.14	14.28	4.11

0	2	438.45	442.48	62.81	58.77
0C	3	435.21	441.28	66.05	59.98
0N1	3	434.02	440.09	67.24	61.17
0N2	4	432.95	441.06	68.31	60.20
0N3	5	431.29	441.46	69.97	59.80
0N4	6	430.08	442.32	71.18	58.94
0CN1	4	430.70	438.81	70.56	62.44
0CN2	5	429.50	439.67	71.76	61.59
0CN3	6	427.42	439.66	73.84	61.60
0CN4	7	425.81	440.13	75.45	61.12
0E1	4	431.72	439.83	69.54	61.43
0E2	5	427.35	437.52	73.91	63.74
0E3	6	426.21	438.45	75.05	62.80
0CE1	5	427.89	438.06	73.37	63.20
0CE2	6	424.12	436.36	77.13	64.89
0CE3	7	422.92	437.24	78.34	64.02
1	3	434.00	440.07	67.25	61.18
1C	4	430.18	438.29	71.08	62.96
1N1	4	429.33	437.44	71.93	63.82
1N2	5	428.20	438.37	73.05	62.88
1N3	6	425.21	437.45	76.04	63.80
1N4	7	422.95	437.27	78.31	63.98
1CN1	5	425.24	435.41	76.01	65.84
1CN2	6	422.63	434.87	78.63	66.39
1CN3	7	420.43	434.75	80.83	66.51
1CN4	8	419.29	435.71	81.96	65.55

1E1	5	426.57	436.74	74.68	64.51
1E2	6	422.47	434.71	78.78	66.54
1E3	7	421.40	435.72	79.86	65.53
1CE1	6	421.34	433.58	79.91	67.67
1CE2	7	419.27	433.59	81.99	67.66
1CE3	8	418.09	434.50	83.17	66.76
2	4	431.38	439.50	69.88	61.76
2C	5	426.66	436.83	74.59	64.42
2N1	5	425.20	435.37	76.06	65.89
2N2	6	424.09	436.33	77.16	64.92
2N3	7	422.50	436.82	78.76	64.44
2N4	8	421.48	437.89	79.78	63.36
2CN1	6	421.78	434.02	79.48	67.24
2CN2	7	420.53	434.86	80.72	66.40
2CN3	8	418.91	435.32	82.35	65.93
2CN4	9	417.98	436.50	83.27	64.75
2E1	6	422.81	435.05	78.45	66.21
2E2	7	421.03	435.35	80.23	65.90
2E3	8	420.03	436.44	81.23	64.82
2CE1	7	***419.25***	***433.57***	***82.01***	***67.69***
2CE2	8	417.60	434.02	83.65	67.24
2CE3	9	416.62	435.14	84.64	66.12

Table B. Model comparison: 3-T Face/House version (Good-learner group). See Fig 2. Listed first for the 72 models fitted to empirical data are absolute scores for deviance and the corrected Akaike information criterion (AICc), where a lower score is better. These absolute scores were translated to residual goodness of fit relative to the null chance model "X", where a higher score is better. Results with the best fit according to the AICc, which penalizes

degrees of freedom, are highlighted with boldface and italics. “df” stands for degrees of freedom. The conventions for displaying this table also apply for Tables C-U.

3-T Face/House		Poor learner (n = 9)			
Empirical data		Absolute		Residual	
Model	df	Dev.	AICc	Dev.	AICc
X	0	489.05	489.05	0	0
XC	1	469.39	471.41	19.66	17.65
XN1	1	483.11	485.12	5.95	3.94
XN2	2	478.00	482.03	11.06	7.02
XN3	3	469.90	475.97	19.16	13.09
XN4	4	463.21	471.33	25.84	17.73
XN5	5	457.77	467.94	31.29	21.11
XN6	6	455.91	468.16	33.14	20.89
XN7	7	452.37	466.70	36.69	22.36
XN8	8	450.42	466.85	38.63	22.21
XCN1	2	463.87	467.91	25.18	21.15
XCN2	3	459.85	465.92	29.20	23.13
XCN3	4	455.48	463.59	33.58	25.46
XCN4	5	450.79	460.96	38.26	28.09
XCN5	6	447.05	459.30	42.00	29.75
XCN6	7	446.03	460.36	43.02	28.69
XCN7	8	443.02	459.45	46.03	29.61
XCN8	9	442.25	460.78	46.81	28.28
XE1	2	454.00	458.03	35.05	31.02
XE2	3	450.93	457.00	38.13	32.06
XE3	4	447.95	456.07	41.10	32.99
XCE1	3	449.67	455.74	39.39	33.32
XCE2	4	446.91	455.02	42.15	34.03
XCE3	5	444.06	454.23	45.00	34.82

0	2	489.04	493.08	0.01	-4.02
0C	3	469.08	475.15	19.98	13.91
0N1	3	483.11	489.18	5.95	-0.12
0N2	4	478.00	486.11	11.06	2.94
0N3	5	444.13	454.30	44.92	34.75
0N4	6	433.89	446.14	55.16	42.92
0CN1	4	463.30	471.42	25.75	17.64
0CN2	5	457.48	467.65	31.58	21.40
0CN3	6	424.81	437.05	64.25	52.00
0CN4	7	423.12	437.45	65.93	51.60
0E1	4	429.77	437.89	59.28	51.16
0E2	5	426.26	436.44	62.79	52.62
0E3	6	424.39	436.64	64.66	52.41
0CE1	5	425.32	435.50	63.73	53.56
0CE2	6	422.28	434.53	66.77	54.52
0CE3	7	420.26	434.59	68.79	54.46
1	3	473.20	479.27	15.86	9.79
1C	4	439.08	447.20	49.97	41.86
1N1	4	452.17	460.29	36.88	28.77
1N2	5	442.74	452.92	46.31	36.13
1N3	6	432.35	444.60	56.70	44.46
1N4	7	429.13	443.45	59.93	45.60
1CN1	5	428.14	438.32	60.91	50.74
1CN2	6	425.11	437.60	63.94	51.70
1CN3	7	421.44	435.77	67.61	53.28
1CN4	8	419.20	435.62	69.86	53.43

1E1	5	426.95	437.12	62.11	51.93
1E2	6	424.15	436.39	64.91	52.66
1E3	7	421.56	435.89	67.49	53.16
1CE1	6	422.23	434.48	66.82	54.58
1CE2	7	419.79	434.12	69.26	54.93
1CE3	8	***417.57***	***434.00***	***71.48***	***55.06***
2	4	466.69	474.80	22.37	14.25
2C	5	432.05	442.23	57.00	46.82
2N1	5	447.10	457.28	41.95	31.77
2N2	6	437.29	449.54	51.76	39.52
2N3	7	430.10	444.43	58.95	44.62
2N4	8	427.17	443.59	61.89	45.46
2CN1	6	425.47	437.72	63.58	51.34
2CN2	7	422.92	437.25	66.13	51.81
2CN3	8	419.49	435.91	69.56	53.14
2CN4	9	417.46	435.99	71.59	53.06
2E1	6	425.45	437.70	63.60	51.35
2E2	7	422.51	436.84	66.55	52.22
2E3	8	420.33	436.76	68.72	52.30
2CE1	7	420.67	435.00	68.39	54.06
2CE2	8	418.48	434.90	70.57	54.15
2CE3	9	416.25	434.78	72.80	54.27

Table C. Model comparison: 3-T Face/House version (Poor-learner group). See Fig 2.

3-T Face/House		Nonlearner (n = 7)			
Empirical data		Absolute		Residual	
Model	df	Dev.	AICc	Dev.	AICc
X	0	481.64	481.64	0	0
XC	1	468.01	470.03	13.62	11.61
XN1	1	467.55	469.56	14.09	12.08
XN2	2	461.17	465.20	20.47	16.44
XN3	3	455.81	461.88	25.83	19.75
XN4	4	452.55	460.66	29.09	20.97
XN5	5	451.03	461.20	30.61	20.43
XN6	6	449.53	461.78	32.10	19.85
XN7	7	447.95	462.28	33.69	19.36
XN8	8	447.06	463.49	34.58	18.15
XCN1	2	457.70	461.73	23.94	19.91
XCN2	3	453.37	459.44	28.27	22.20
XCN3	4	449.22	457.34	32.42	24.30
XCN4	5	446.57	456.75	35.07	24.89
XCN5	6	445.82	458.07	35.82	23.57
XCN6	7	444.19	458.52	37.45	23.11
XCN7	8	443.07	459.50	38.57	22.14
XCN8	9	442.38	460.92	39.26	20.72
XE1	2	457.75	461.79	23.89	19.85
XE2	3	448.25	454.32	33.39	27.31
XE3	4	447.42	455.54	34.21	26.10
XCE1	3	452.72	458.79	28.92	22.85
XCE2	4	***445.46***	***453.58***	***36.18***	***28.06***
XCE3	5	444.58	454.75	37.06	26.88

0	2	481.41	485.44	0.23	-3.80
0C	3	468.01	474.09	13.62	7.55
0N1	3	467.55	473.62	14.09	8.02
0N2	4	460.44	468.56	21.20	13.08
0N3	5	455.23	465.41	26.40	16.23
0N4	6	452.14	464.38	29.50	17.25
0CN1	4	457.44	465.56	24.20	16.08
0CN2	5	453.12	463.30	28.52	18.34
0CN3	6	448.93	461.18	32.71	20.46
0CN4	7	446.35	460.68	35.29	20.95
0E1	4	457.75	465.86	23.89	15.77
0E2	5	448.10	458.28	33.53	23.36
0E3	6	447.12	459.37	34.51	22.27
0CE1	5	452.50	462.68	29.13	18.96
0CE2	6	445.17	457.42	36.47	24.22
0CE3	7	444.31	458.64	37.33	23.00
1	3	481.09	487.16	0.54	-5.53
1C	4	467.50	475.62	14.14	6.02
1N1	4	465.70	473.82	15.94	7.82
1N2	5	459.69	469.87	21.95	11.77
1N3	6	454.62	466.87	27.01	14.77
1N4	7	451.29	465.63	30.35	16.01
1CN1	5	457.07	467.25	24.57	14.39
1CN2	6	452.43	464.68	29.21	16.96
1CN3	7	448.43	462.77	33.21	18.87
1CN4	8	445.78	462.21	35.85	19.42

1E1	5	456.80	466.98	24.84	14.66
1E2	6	447.40	459.65	34.23	21.99
1E3	7	446.64	460.98	34.99	20.66
1CE1	6	452.06	464.31	29.58	17.33
1CE2	7	444.67	459.00	36.97	22.64
1CE3	8	443.84	460.27	37.80	21.37
2	4	477.44	485.55	4.20	-3.91
2C	5	465.74	475.92	15.90	5.72
2N1	5	463.53	473.71	18.11	7.93
2N2	6	457.80	470.05	23.84	11.59
2N3	7	453.07	467.40	28.57	14.24
2N4	8	450.34	466.77	31.30	14.87
2CN1	6	455.60	467.85	26.04	13.79
2CN2	7	451.35	465.68	30.29	15.95
2CN3	8	447.69	464.12	33.94	17.51
2CN4	9	445.20	463.74	36.44	17.90
2E1	6	445.81	468.06	25.82	13.58
2E2	7	446.51	460.84	35.13	20.80
2E3	8	445.70	462.13	35.93	19.50
2CE1	7	451.26	465.60	30.37	16.04
2CE2	8	443.76	460.19	37.88	21.45
2CE3	9	442.94	461.48	38.70	20.16

Table D. Model comparison: 3-T Face/House version (Nonlearner group). See Fig 2.

7-T Color/Motion<br>Empirical data<br>Model	 <br>df	Good learner (n = 16)<br>Absolute<br>Dev.	 <br>AICc	 Residual<br>Dev.	 <br>AICc
X	0	478.79	478.79	0	0
XC	1	470.80	472.82	7.99	5.98
XN1	1	474.34	476.35	4.45	2.44
XN2	2	471.87	475.90	6.92	2.89
XN3	3	470.31	476.38	8.48	2.41
XN4	4	467.83	475.95	10.96	2.84
XN5	5	465.56	475.74	13.23	3.05
XN6	6	464.61	476.86	14.18	1.93
XN7	7	462.95	477.28	15.85	1.51
XN8	8	462.02	478.45	16.78	0.34
XCN1	2	464.53	468.56	14.26	10.23
XCN2	3	463.00	469.07	15.79	9.72
XCN3	4	461.79	469.91	17.01	8.89
XCN4	5	460.43	470.61	18.36	8.18
XCN5	6	458.95	471.20	19.84	7.59
XCN6	7	457.90	472.24	20.89	6.55
XCN7	8	456.12	472.55	22.67	6.24
XCN8	9	455.61	474.15	23.18	4.64
XE1	2	467.56	471.59	11.23	7.20
XE2	3	465.48	471.56	13.31	7.24
XE3	4	464.06	472.18	14.73	6.61
XCE1	3	461.66	467.73	17.13	11.06
XCE2	4	460.94	469.05	17.86	9.74
XCE3	5	459.72	469.90	19.07	8.89

0	2	454.26	458.29	24.53	20.50
0C	3	443.46	449.53	35.33	29.26
0N1	3	444.00	450.07	34.80	28.72
0N2	4	429.41	437.53	49.38	41.27
0N3	5	427.01	437.19	51.79	41.61
0N4	6	424.14	436.39	54.65	42.40
0CN1	4	428.99	437.11	49.80	41.68
0CN2	5	420.31	430.49	58.48	48.30
0CN3	6	417.28	429.53	61.51	49.26
0CN4	7	412.93	427.27	65.86	51.52
0E1	4	440.25	448.37	38.54	30.42
0E2	5	421.38	431.55	57.42	47.24
0E3	6	417.18	429.44	61.61	49.36
0CE1	5	423.83	434.01	54.96	44.78
0CE2	6	413.25	425.50	65.54	53.29
0CE3	7	410.79	425.12	68.01	53.67
1	3	436.64	442.71	42.15	36.08
1C	4	415.90	424.02	62.89	54.77
1N1	4	425.40	433.52	53.39	45.27
1N2	5	417.14	427.32	61.65	51.47
1N3	6	411.49	423.75	67.30	55.05
1N4	7	409.49	423.82	69.30	54.97
1CN1	5	407.44	417.62	71.35	61.17
1CN2	6	405.97	418.22	72.82	60.57
1CN3	7	404.58	418.91	74.22	59.88
1CN4	8	402.64	419.07	76.16	59.72

1E1	5	410.41	420.59	68.38	58.20
1E2	6	408.38	420.63	70.41	58.16
1E3	7	406.98	421.31	71.82	57.48
1CE1	6	404.13	416.39	74.66	62.41
1CE2	7	403.11	417.45	75.68	61.34
1CE3	8	402.21	418.64	76.58	60.15
2	4	415.73	423.85	63.06	54.94
2C	5	403.95	414.13	74.85	64.67
2N1	5	405.96	416.13	72.84	62.66
2N2	6	404.04	416.29	74.75	62.50
2N3	7	402.83	417.16	75.97	61.63
2N4	8	400.92	417.36	77.87	61.44
2CN1	6	399.09	411.34	79.71	67.45
2CN2	7	397.72	412.06	81.07	66.73
2CN3	8	396.55	412.99	82.24	65.80
2CN4	9	394.97	413.51	83.82	65.28
2E1	6	403.02	415.27	75.77	63.52
2E2	7	401.19	415.53	77.60	63.26
2E3	8	399.92	416.36	78.87	62.44
2CE1	7	***396.84***	***411.17***	***81.95***	***67.62***
2CE2	8	395.77	412.20	83.02	66.59
2CE3	9	394.97	413.51	83.82	65.28

Table E. Model comparison: 7-T Color/Motion version (Good-learner group). See Fig 3.

7-T Color/Motion		Poor learner (*n* = 5)			
Empirical data		Absolute		Residual	
Model	df	Dev.	AICc	Dev.	AICc
X	0	447.50	447.50	0	0
XC	1	428.76	430.78	18.73	16.72
XN1	1	416.56	418.58	30.93	28.92
XN2	2	406.48	410.52	41.01	36.97
XN3	3	405.69	411.77	41.81	35.73
XN4	4	401.38	409.52	46.11	37.98
XN5	5	400.74	410.94	46.76	36.56
XN6	6	397.44	409.72	50.06	37.78
XN7	7	395.91	410.29	51.58	37.21
XN8	8	395.16	411.65	52.34	35.85
XCN1	2	404.20	408.24	43.29	39.25
XCN2	3	395.17	401.25	52.33	46.25
XCN3	4	394.53	402.66	52.97	44.83
XCN4	5	391.95	402.15	55.54	45.34
XCN5	6	391.13	403.41	56.37	44.08
XCN6	7	389.38	403.76	58.12	43.74
XCN7	8	388.17	404.66	59.32	42.84
XCN8	9	387.67	406.29	59.82	41.21
XE1	2	402.00	406.04	45.50	41.46
XE2	3	396.59	402.67	50.91	44.83
XE3	4	395.30	403.43	52.20	44.06
XCE1	3	396.93	403.01	50.57	44.49
XCE2	4	392.14	400.28	55.35	47.22
XCE3	5	391.67	401.87	55.83	45.63

0	2	444.86	448.90	2.64	-1.40
0C	3	424.23	430.31	23.27	17.19
0N1	3	413.35	419.43	34.15	28.07
0N2	4	403.50	411.63	44.00	35.86
0N3	5	400.96	411.16	46.54	36.34
0N4	6	397.56	409.85	49.93	37.65
0CN1	4	400.45	408.58	47.05	38.92
0CN2	5	391.63	401.83	55.87	45.66
0CN3	6	390.56	402.84	56.93	44.65
0CN4	7	388.74	403.12	58.76	44.38
0E1	4	398.39	406.52	49.10	40.97
0E2	5	393.96	404.16	53.54	43.34
0E3	6	392.65	404.93	54.85	42.56
0CE1	5	393.22	403.42	54.27	44.07
0CE2	6	388.98	401.26	58.52	46.24
0CE3	7	387.97	402.35	59.53	45.15
1	3	427.93	434.01	19.57	13.49
1C	4	416.62	424.76	30.87	22.74
1N1	4	403.00	411.13	44.50	36.36
1N2	5	393.54	403.74	53.96	43.76
1N3	6	392.54	404.82	54.96	42.67
1N4	7	389.23	403.61	58.27	43.89
1CN1	5	394.84	405.04	52.66	42.46
1CN2	6	385.87	398.15	61.62	49.34
1CN3	7	385.28	399.66	62.21	47.84
1CN4	8	383.23	399.72	64.27	47.78

1E1	5	391.87	402.07	55.63	45.43
1E2	6	387.20	399.48	60.29	48.01
1E3	7	386.32	400.70	61.17	46.79
1CE1	6	387.72	400.00	59.77	47.49
1CE2	7	***383.31***	***397.69***	***64.19***	***49.81***
1CE3	8	382.86	399.35	64.64	48.15
2	4	426.37	434.51	21.12	12.99
2C	5	415.89	426.09	31.60	21.40
2N1	5	402.03	412.23	45.47	35.27
2N2	6	392.33	404.61	55.16	42.88
2N3	7	391.71	406.09	55.78	41.41
2N4	8	388.80	405.28	58.70	42.21
2CN1	6	393.88	406.16	53.61	41.33
2CN2	7	384.97	399.34	62.53	48.15
2CN3	8	384.54	401.03	62.95	46.46
2CN4	9	382.82	401.44	64.67	46.06
2E1	6	390.92	403.20	56.58	44.29
2E2	7	386.02	400.40	61.47	47.09
2E3	8	385.09	401.57	62.41	45.92
2CE1	7	386.62	401.00	60.88	46.50
2CE2	8	382.00	398.49	65.49	49.00
2CE3	9	381.44	400.05	66.06	47.45

Table F. Model comparison: 7-T Color/Motion version (Poor-learner group). See Fig 3.

3-T Face/House 2CE1 simulation		Good learner (n = 31)			
		Absolute		Residual	
Model	df	Dev.	AICc	Dev.	AICc
X	0	501.26	501.26	0	0
XC	1	496.29	498.30	4.97	2.96
XN1	1	495.09	497.10	6.17	4.16
XN2	2	493.42	497.45	7.84	3.80
XN3	3	492.42	498.49	8.84	2.77
XN4	4	491.49	499.60	9.77	1.66
XN5	5	490.54	500.71	10.72	0.55
XN6	6	488.98	501.23	12.27	0.03
XN7	7	488.38	502.70	12.88	-1.44
XN8	8	487.14	503.56	14.11	-2.30
XCN1	2	489.84	493.88	11.41	7.38
XCN2	3	488.34	494.41	12.92	6.85
XCN3	4	487.17	495.28	14.09	5.97
XCN4	5	486.38	496.56	14.87	4.70
XCN5	6	485.61	497.85	15.64	3.40
XCN6	7	484.27	498.60	16.98	2.66
XCN7	8	483.72	500.14	17.53	1.12
XCN8	9	482.68	501.20	18.58	0.06
XE1	2	491.73	495.76	9.53	5.50
XE2	3	489.79	495.86	11.47	5.40
XE3	4	488.75	496.86	12.51	4.40
XCE1	3	487.14	493.20	14.12	8.05
XCE2	4	485.51	493.63	15.74	7.63
XCE3	5	484.36	494.53	16.90	6.73

0	2	439.61	443.64	61.65	57.62
0C	3	435.38	441.45	65.88	59.81
0N1	3	434.68	440.75	66.57	60.51
0N2	4	430.56	438.67	70.70	62.59
0N3	5	426.39	436.57	74.86	64.69
0N4	6	425.73	437.97	75.53	63.29
0CN1	4	429.79	437.90	71.47	63.36
0CN2	5	425.64	435.81	75.62	65.45
0CN3	6	421.15	433.39	80.11	67.87
0CN4	7	420.46	434.78	80.80	66.47
0E1	4	429.15	437.27	72.11	63.99
0E2	5	426.67	436.84	74.59	64.42
0E3	6	420.56	432.80	80.70	68.46
0CE1	5	423.30	433.47	77.96	67.79
0CE2	6	419.72	431.96	81.54	69.30
0CE3	7	416.03	430.35	85.23	70.90
1	3	429.20	435.26	72.06	65.99
1C	4	421.98	430.10	79.27	71.16
1N1	4	421.22	429.33	80.04	71.92
1N2	5	417.14	427.32	84.11	73.94
1N3	6	416.27	428.51	84.99	72.75
1N4	7	415.57	429.89	85.69	71.36
1CN1	5	416.83	427.00	84.43	74.26
1CN2	6	412.80	425.04	88.46	76.22
1CN3	7	411.78	426.11	89.47	75.15
1CN4	8	411.12	427.54	90.13	73.72

1E1	5	418.15	428.33	83.10	72.93
1E2	6	413.87	426.11	87.39	75.14
1E3	7	413.23	427.55	88.03	73.71
1CE1	6	410.90	423.14	90.36	78.12
1CE2	7	409.20	423.53	92.05	77.73
1CE3	8	408.48	424.90	92.78	76.36
2	4	424.76	432.88	76.49	68.38
2C	5	417.83	428.01	83.42	73.25
2N1	5	417.14	427.31	84.11	73.94
2N2	6	415.57	427.81	85.69	73.45
2N3	7	414.66	428.99	86.59	72.27
2N4	8	413.91	430.32	87.35	70.93
2CN1	6	412.74	424.98	88.52	76.28
2CN2	7	411.16	425.49	90.09	75.77
2CN3	8	410.14	426.55	91.12	74.71
2CN4	9	409.46	427.98	91.80	73.28
2E1	6	413.64	425.88	87.62	75.37
2E2	7	411.97	426.29	89.29	74.96
2E3	8	411.25	427.66	90.01	73.59
2CE1	7	***408.67***	***422.99***	***92.59***	***78.27***
2CE2	8	407.43	423.85	93.82	77.41
2CE3	9	406.70	425.22	94.56	76.04

Table G. Discriminability of the 2CE1 model: 3-T Face/House version (Good-learner group). See Fig C.

3-T Face/House 2CE1 simulation		Poor learner (n = 9)			
		Absolute		Residual	
Model	df	Dev.	AICc	Dev.	AICc
X	0	489.05	489.05	0	0
XC	1	461.11	463.12	27.95	25.93
XN1	1	476.81	478.82	12.25	10.23
XN2	2	473.27	477.31	15.78	11.75
XN3	3	469.39	475.46	19.66	13.59
XN4	4	462.10	470.21	26.96	18.84
XN5	5	460.11	470.28	28.95	18.77
XN6	6	457.06	469.31	31.99	19.74
XN7	7	453.91	468.24	35.14	20.81
XN8	8	452.02	468.44	37.03	20.61
XCN1	2	450.08	454.12	38.97	34.94
XCN2	3	448.31	454.38	40.74	34.67
XCN3	4	447.69	455.80	41.37	33.25
XCN4	5	444.40	454.58	44.65	34.47
XCN5	6	443.89	456.14	45.16	32.91
XCN6	7	442.54	456.87	46.51	32.18
XCN7	8	441.22	457.65	47.83	31.41
XCN8	9	440.51	459.05	48.54	30.01
XE1	2	456.39	460.42	32.67	28.63
XE2	3	450.39	456.46	38.66	32.59
XE3	4	448.48	456.60	40.57	32.45
XCE1	3	446.53	452.60	42.53	36.46
XCE2	4	444.49	452.61	44.56	36.44
XCE3	5	442.85	453.02	46.21	36.03

0	2	487.68	491.71	1.38	-2.66
0C	3	458.08	464.15	30.97	24.90
0N1	3	475.87	481.94	13.18	7.11
0N2	4	471.91	480.03	17.14	9.02
0N3	5	463.01	473.18	26.05	15.87
0N4	6	431.64	443.89	57.41	45.16
0CN1	4	422.22	430.34	66.83	58.72
0CN2	5	421.01	431.18	68.05	57.87
0CN3	6	420.12	432.37	68.93	56.68
0CN4	7	418.07	432.40	70.98	56.65
0E1	4	428.20	436.32	60.85	52.73
0E2	5	423.10	433.28	65.95	55.77
0E3	6	422.40	434.65	66.65	54.40
0CE1	5	418.93	429.11	70.12	59.95
0CE2	6	417.77	430.02	71.28	59.04
0CE3	7	417.27	431.60	71.78	57.45
1	3	469.94	476.01	19.12	13.05
1C	4	430.64	438.76	58.41	50.29
1N1	4	445.44	453.55	43.62	35.50
1N2	5	436.52	446.70	52.53	42.35
1N3	6	432.78	445.02	56.28	44.03
1N4	7	426.98	441.31	62.08	47.75
1CN1	5	419.18	429.36	69.87	59.69
1CN2	6	417.37	429.61	71.69	59.44
1CN3	7	416.59	430.92	72.46	58.13
1CN4	8	414.29	430.71	74.77	58.34

1E1	5	425.30	435.48	63.75	53.57
1E2	6	420.15	432.39	68.91	56.66
1E3	7	418.87	433.20	70.18	55.85
1CE1	6	415.81	428.06	73.24	61.00
1CE2	7	414.74	429.07	74.32	59.99
1CE3	8	413.98	430.41	75.07	58.65
2	4	468.23	476.35	20.82	12.71
2C	5	428.12	438.29	60.94	50.76
2N1	5	437.88	448.05	51.18	41.00
2N2	6	432.07	444.32	56.98	44.74
2N3	7	427.33	441.66	61.72	47.39
2N4	8	424.17	440.60	64.88	48.46
2CN1	6	417.10	429.35	71.95	59.71
2CN2	7	414.71	429.04	74.34	60.01
2CN3	8	414.31	430.74	74.74	58.32
2CN4	9	412.80	431.33	76.26	57.73
2E1	6	422.48	434.73	66.57	54.33
2E2	7	417.65	431.98	71.41	57.08
2E3	8	416.89	433.31	72.17	55.74
2CE1	7	***413.59***	***427.92***	***75.47***	***61.14***
2CE2	8	412.43	428.85	76.62	60.20
2CE3	9	411.79	430.32	77.26	58.73

Table H. Discriminability of the 2CE1 model: 3-T Face/House version (Poor-learner group). See Fig C.

3-T Face/House 2CE1 simulation		Nonlearner (n = 7)			
		Absolute		Residual	
Model	df	Dev.	AICc	Dev.	AICc
X	0	481.64	481.64	0	0
XC	1	466.24	468.25	15.40	13.39
XN1	1	467.46	469.47	14.18	12.16
XN2	2	461.12	465.15	20.52	16.49
XN3	3	457.81	463.88	23.83	17.76
XN4	4	456.55	464.67	25.09	16.97
XN5	5	453.30	463.47	28.34	18.16
XN6	6	452.09	464.34	29.55	17.30
XN7	7	451.35	465.69	30.29	15.95
XN8	8	450.77	467.20	30.87	14.44
XCN1	2	456.85	460.88	24.79	20.76
XCN2	3	453.09	459.16	28.55	22.48
XCN3	4	450.91	459.03	30.72	22.61
XCN4	5	449.86	460.03	31.78	21.61
XCN5	6	447.67	459.92	33.96	21.71
XCN6	7	446.73	461.06	34.91	20.57
XCN7	8	446.18	462.61	35.46	19.03
XCN8	9	445.26	463.80	36.38	17.84
XE1	2	453.38	457.42	28.26	24.22
XE2	3	451.05	457.13	30.58	24.51
XE3	4	450.68	458.79	30.96	22.84
XCE1	3	***448.16***	***454.23***	***33.47***	***27.40***
XCE2	4	447.40	455.52	34.24	26.12
XCE3	5	446.83	457.00	34.81	24.63

0	2	481.02	485.05	0.62	-3.42
0C	3	465.70	471.77	15.94	9.87
0N1	3	466.85	472.92	14.78	8.71
0N2	4	460.51	468.63	21.13	13.01
0N3	5	456.83	467.00	24.81	14.63
0N4	6	455.43	467.68	26.21	13.96
0CN1	4	456.32	464.44	25.32	17.20
0CN2	5	452.56	462.73	29.08	18.90
0CN3	6	449.90	462.15	31.73	19.48
0CN4	7	448.89	463.22	32.75	18.41
0E1	4	452.35	460.47	29.29	21.17
0E2	5	450.01	460.19	31.63	21.45
0E3	6	449.31	461.56	32.32	20.07
0CE1	5	447.26	457.44	34.38	24.20
0CE2	6	445.96	458.21	35.68	23.43
0CE3	7	445.46	459.79	36.18	21.85
1	3	479.19	485.26	2.45	-3.62
1C	4	463.85	471.97	17.79	9.67
1N1	4	465.15	473.27	16.49	8.37
1N2	5	458.41	468.59	23.22	13.05
1N3	6	454.96	467.21	26.68	14.43
1N4	7	453.26	467.59	28.38	14.04
1CN1	5	454.53	464.71	27.10	16.93
1CN2	6	450.18	462.43	31.46	19.21
1CN3	7	448.44	462.77	33.20	18.87
1CN4	8	446.90	463.33	34.74	18.31

1E1	5	450.78	460.96	30.86	20.68
1E2	6	448.61	460.85	33.03	20.78
1E3	7	447.58	461.91	34.06	19.73
1CE1	6	445.04	457.29	36.60	24.35
1CE2	7	444.03	458.37	37.61	23.27
1CE3	8	443.56	459.99	38.08	21.65
2	4	472.92	481.04	8.72	0.60
2C	5	460.39	470.56	21.25	11.08
2N1	5	460.79	470.96	20.85	10.67
2N2	6	455.87	468.12	25.77	13.52
2N3	7	452.71	467.04	28.93	14.60
2N4	8	451.47	467.90	30.16	13.73
2CN1	6	451.85	464.10	29.79	17.54
2CN2	7	448.62	462.95	33.02	18.68
2CN3	8	446.46	462.89	35.18	18.75
2CN4	9	445.35	463.89	36.29	17.75
2E1	6	448.98	461.23	32.66	20.41
2E2	7	446.71	461.04	34.93	20.60
2E3	8	446.12	462.55	53.52	19.09
2CE1	7	443.28	457.61	38.36	24.03
2CE2	8	442.59	459.02	39.04	22.61
2CE3	9	442.14	460.68	39.49	20.96

Table I. Discriminability of the 2CE1 model: 3-T Face/House version (Nonlearner group). See Fig C.

7-T Color/Motion 2CE1 simulation		Good learner (n = 16)			
		Absolute		Residual	
Model	df	Dev.	AICc	Dev.	AICc
X	0	478.79	478.79	0	0
XC	1	472.41	474.42	6.38	4.37
XN1	1	472.53	474.54	6.26	4.25
XN2	2	469.86	473.89	8.93	4.90
XN3	3	467.94	474.01	10.85	4.78
XN4	4	467.04	475.16	11.75	3.63
XN5	5	465.69	475.87	13.10	2.92
XN6	6	464.99	477.24	13.81	1.55
XN7	7	463.55	477.88	15.24	0.91
XN8	8	461.15	477.59	17.64	1.21
XCN1	2	464.28	468.31	14.51	10.48
XCN2	3	461.71	467.78	17.08	11.01
XCN3	4	460.16	468.28	18.64	10.52
XCN4	5	459.19	469.37	19.60	9.42
XCN5	6	457.88	470.13	20.91	8.66
XCN6	7	457.31	471.64	21.48	7.15
XCN7	8	456.21	472.65	22.58	6.14
XCN8	9	454.10	472.64	24.69	6.15
XE1	2	467.39	471.43	11.40	7.36
XE2	3	464.30	470.37	14.49	8.42
XE3	4	462.82	470.94	15.97	7.85
XCE1	3	459.79	465.86	19.00	12.93
XCE2	4	457.79	465.91	21.00	12.88
XCE3	5	456.77	466.95	22.02	11.84

0	2	454.67	458.71	24.12	20.08
0C	3	449.42	455.49	29.37	23.30
0N1	3	450.22	456.29	28.57	22.50
0N2	4	445.95	454.07	32.84	24.72
0N3	5	436.34	446.52	42.45	32.27
0N4	6	429.86	442.11	48.93	36.68
0CN1	4	443.60	451.72	35.19	27.07
0CN2	5	431.32	441.50	47.47	37.30
0CN3	6	421.86	434.11	56.94	44.68
0CN4	7	414.98	429.32	63.81	49.47
0E1	4	437.94	446.06	40.85	32.73
0E2	5	418.50	428.68	60.29	50.11
0E3	6	414.77	427.02	64.02	51.77
0CE1	5	411.55	421.72	67.25	57.07
0CE2	6	409.59	421.84	69.20	56.95
0CE3	7	407.97	422.31	70.82	56.48
1	3	431.88	437.95	46.92	40.84
1C	4	426.56	434.68	52.23	44.11
1N1	4	416.16	424.28	62.63	54.51
1N2	5	412.96	423.14	65.83	55.65
1N3	6	408.92	421.17	69.87	57.62
1N4	7	407.15	421.48	71.65	57.31
1CN1	5	408.78	418.95	70.02	59.84
1CN2	6	405.69	417.95	73.10	60.85
1CN3	7	401.68	416.02	77.11	62.77
1CN4	8	400.37	416.80	78.42	61.99

1E1	5	407.52	417.69	71.28	61.10
1E2	6	404.52	416.77	74.28	62.02
1E3	7	403.10	417.43	75.69	61.36
1CE1	6	399.57	411.82	79.22	66.97
1CE2	7	397.29	411.62	81.51	67.17
1CE3	8	396.21	412.64	82.58	66.15
2	4	409.09	417.21	69.70	61.58
2C	5	403.75	413.93	75.04	64.86
2N1	5	404.12	414.30	74.67	64.49
2N2	6	401.99	414.24	76.80	64.55
2N3	7	400.66	415.00	78.13	63.79
2N4	8	399.67	416.10	79.12	62.69
2CN1	6	396.80	409.05	81.99	69.74
2CN2	7	394.91	409.24	83.88	69.55
2CN3	8	393.83	410.27	84.96	68.52
2CN4	9	392.75	411.29	86.05	67.50
2E1	6	399.82	412.07	78.97	66.72
2E2	7	397.23	411.57	81.56	67.22
2E3	8	396.44	412.88	82.35	65.92
2CE1	7	***392.63***	***406.96***	***86.16***	***71.83***
2CE2	8	390.55	406.98	88.24	71.81
2CE3	9	389.57	408.11	89.22	70.68

Table J. Discriminability of the 2CE1 model: 7-T Color/Motion version (Good-learner group). See Fig D.

7-T Color/Motion 2CE1 simulation		Poor learner (n = 5)			
		Absolute		Residual	
Model	df	Dev.	AICc	Dev.	AICc
X	0	447.50	447.50	0	0
XC	1	420.10	422.11	27.39	25.38
XN1	1	408.40	410.41	39.10	37.08
XN2	2	405.50	409.54	42.00	37.96
XN3	3	401.54	407.62	45.96	39.88
XN4	4	399.56	407.69	47.94	39.80
XN5	5	396.85	407.05	50.64	40.44
XN6	6	394.89	407.17	52.61	40.33
XN7	7	393.35	407.72	54.15	39.77
XN8	8	391.31	407.79	56.19	39.70
XCN1	2	393.47	397.51	54.02	49.98
XCN2	3	390.93	397.01	56.57	50.49
XCN3	4	389.76	397.89	57.74	49.60
XCN4	5	387.96	398.16	59.54	49.34
XCN5	6	386.14	398.42	61.36	49.08
XCN6	7	385.05	399.43	62.44	48.06
XCN7	8	384.55	401.04	62.94	46.45
XCN8	9	383.61	402.22	63.89	45.28
XE1	2	396.39	400.43	51.11	47.07
XE2	3	394.99	401.07	52.51	46.43
XE3	4	393.69	401.82	53.81	45.67
XCE1	3	391.11	397.19	56.38	50.30
XCE2	4	389.58	397.72	57.91	49.78
XCE3	5	388.22	398.42	59.28	49.08

0	2	440.50	444.54	6.99	2.95
0C	3	415.39	421.47	32.11	26.03
0N1	3	403.27	409.35	44.22	38.14
0N2	4	400.96	409.09	46.54	38.40
0N3	5	395.86	406.06	51.64	41.44
0N4	6	389.37	401.66	58.12	45.84
0CN1	4	384.62	392.75	62.88	54.74
0CN2	5	382.39	392.59	65.11	54.91
0CN3	6	380.29	392.57	67.21	54.93
0CN4	7	378.26	392.64	69.24	54.86
0E1	4	391.91	400.05	55.58	47.45
0E2	5	390.27	400.47	57.23	47.03
0E3	6	384.54	396.82	62.96	50.67
0CE1	5	381.87	392.07	65.62	55.42
0CE2	6	380.60	392.88	66.90	54.62
0CE3	7	378.33	392.70	69.17	54.79
1	3	431.15	437.23	16.34	10.26
1C	4	409.54	417.67	37.96	29.82
1N1	4	392.32	400.46	55.17	47.04
1N2	5	389.21	399.41	58.28	48.08
1N3	6	385.85	398.13	61.64	49.36
1N4	7	383.92	398.29	63.58	49.20
1CN1	5	378.73	388.93	68.76	58.56
1CN2	6	375.34	387.62	72.16	59.88
1CN3	7	374.57	388.95	72.92	58.54
1CN4	8	372.65	389.14	74.85	58.36

1E1	5	382.26	392.46	65.24	55.03
1E2	6	380.94	393.22	66.56	54.28
1E3	7	378.99	393.36	68.51	54.13
1CE1	6	375.87	388.16	71.62	59.34
1CE2	7	373.94	388.32	73.56	59.18
1CE3	8	372.52	389.00	74.98	58.49
2	4	421.81	429.94	25.68	17.55
2C	5	403.83	414.03	43.66	33.46
2N1	5	385.79	395.99	61.70	51.50
2N2	6	382.87	395.15	64.62	52.34
2N3	7	381.11	395.49	66.39	52.01
2N4	8	379.52	396.01	67.97	51.48
2CN1	6	375.05	387.33	72.45	60.16
2CN2	7	371.72	386.09	75.78	61.40
2CN3	8	370.62	387.11	76.88	60.39
2CN4	9	368.75	387.36	78.75	60.14
2E1	6	378.58	390.86	68.92	56.63
2E2	7	376.77	391.15	70.73	56.35
2E3	8	374.81	391.30	72.68	56.19
2CE1	7	***371.68***	***386.06***	***75.81***	***61.44***
2CE2	8	369.59	386.08	77.90	61.14
2CE3	9	367.82	386.43	79.67	61.06

Table K. Discriminability of the 2CE1 model: 7-T Color/Motion version (Poor-learner group). See Fig D.

3-T Face/House "2" simulation		Good learner (n = 31)			
		Absolute		Residual	
Model	df	Dev.	AICc	Dev.	AICc
X	0	501.26	501.26	0	0
XC	1	500.40	502.41	0.86	-1.16
XN1	1	499.44	501.46	1.81	-0.20
XN2	2	497.43	501.47	3.83	-0.21
XN3	3	495.13	501.20	6.13	0.06
XN4	4	492.83	500.94	8.43	0.32
XN5	5	492.66	502.83	8.60	-1.58
XN6	6	492.62	504.86	8.64	-3.60
XN7	7	492.39	506.71	8.87	-5.45
XN8	8	491.85	508.27	9.41	-7.01
XCN1	2	498.05	502.08	3.21	-0.83
XCN2	3	496.05	502.12	5.20	-0.86
XCN3	4	494.01	502.12	7.25	-0.87
XCN4	5	491.57	501.74	9.69	-0.48
XCN5	6	491.57	503.81	9.69	-2.55
XCN6	7	491.53	505.85	9.73	-4.59
XCN7	8	491.39	507.81	9.87	-6.55
XCN8	9	490.66	509.18	10.60	-7.92
XE1	2	498.14	502.17	3.12	-0.92
XE2	3	495.97	502.04	5.29	-0.78
XE3	4	494.26	502.37	7.00	-1.12
XCE1	3	496.80	502.87	4.46	-1.61
XCE2	4	495.06	503.17	6.20	-1.92
XCE3	5	493.30	503.47	7.96	-2.21

0	2	444.81	448.84	56.45	52.41
0C	3	444.19	450.25	57.07	51.00
0N1	3	443.81	449.88	57.45	51.38
0N2	4	439.44	447.55	61.82	53.71
0N3	5	436.64	446.81	64.62	54.44
0N4	6	432.43	444.68	68.82	56.58
0CN1	4	441.80	449.92	59.46	51.34
0CN2	5	431.60	441.77	69.66	59.48
0CN3	6	431.44	443.68	69.82	57.58
0CN4	7	430.68	445.00	70.57	56.25
0E1	4	439.33	447.45	61.92	53.81
0E2	5	431.64	441.81	69.62	59.45
0E3	6	431.18	443.42	70.07	57.83
0CE1	5	432.55	442.72	68.71	58.54
0CE2	6	430.70	442.94	70.56	58.32
0CE3	7	430.22	444.54	71.04	56.72
1	3	431.33	437.40	69.93	63.86
1C	4	428.59	436.71	72.66	64.55
1N1	4	429.11	437.23	72.14	64.03
1N2	5	425.21	435.38	76.05	65.88
1N3	6	425.01	437.25	76.24	64.00
1N4	7	424.47	438.80	76.78	62.46
1CN1	5	425.91	436.08	75.35	65.18
1CN2	6	423.66	435.90	77.60	65.36
1CN3	7	423.55	437.87	77.71	63.39
1CN4	8	422.99	439.40	78.27	61.86

1E1	5	425.73	435.90	75.53	65.35
1E2	6	424.07	436.31	77.19	64.95
1E3	7	423.62	437.94	77.64	63.32
1CE1	6	424.80	437.04	76.46	64.22
1CE2	7	422.95	437.28	78.30	63.98
1CE3	8	422.47	438.89	78.78	62.37
2	4	***426.14***	***434.26***	***75.11***	***67.00***
2C	5	425.45	435.62	75.81	65.64
2N1	5	425.06	435.23	76.20	66.03
2N2	6	423.31	435.55	77.95	65.71
2N3	7	423.14	437.47	78.11	63.79
2N4	8	422.66	439.08	78.59	62.18
2CN1	6	423.96	436.20	77.30	65.06
2CN2	7	421.88	436.20	79.38	65.06
2CN3	8	421.77	438.18	79.49	63.07
2CN4	9	421.30	439.82	79.96	61.44
2E1	6	423.79	436.03	77.47	65.23
2E2	7	422.19	436.51	79.07	64.75
2E3	8	421.75	438.16	79.51	63.09
2CE1	7	422.85	437.17	78.41	64.09
2CE2	8	421.17	437.59	80.08	63.67
2CE3	9	420.69	439.21	80.57	62.05

Table L. Discriminability of the no-bias model “2” with only GRL: 3-T Face/House version (Good-learner group). See Fig E.

3-T Face/House "2" simulation		Poor learner (*n* = 9)			
		Absolute		Residual	
Model	df	Dev.	AICc	Dev.	AICc
X	0	489.05	489.05	0	0
XC	1	487.32	489.33	1.74	-0.27
XN1	1	487.11	489.12	1.95	-0.06
XN2	2	484.17	488.20	4.88	0.85
XN3	3	481.72	487.79	7.33	1.26
XN4	4	479.02	487.14	10.03	1.91
XN5	5	478.92	489.10	10.13	-0.04
XN6	6	478.78	491.03	10.27	-1.97
XN7	7	478.37	492.69	10.69	-3.64
XN8	8	477.50	493.92	11.56	-4.87
XCN1	2	484.92	488.96	4.13	0.10
XCN2	3	483.20	489.27	5.85	-0.22
XCN3	4	480.00	488.12	9.05	0.93
XCN4	5	477.74	487.91	11.32	1.14
XCN5	6	477.69	489.93	11.37	-0.88
XCN6	7	477.54	491.87	11.51	-2.82
XCN7	8	476.93	493.35	12.13	-4.30
XCN8	9	476.28	494.81	12.77	-5.76
XE1	2	482.96	487.00	6.09	2.05
XE2	3	479.99	486.06	9.06	2.99
XE3	4	478.53	486.65	10.52	2.40
XCE1	3	481.27	487.34	7.78	1.71
XCE2	4	479.33	487.45	9.72	1.61
XCE3	5	477.83	488.01	11.22	1.04

0	2	475.17	479.20	13.88	9.85
0C	3	468.81	474.88	20.24	14.17
0N1	3	473.85	479.92	15.20	9.13
0N2	4	463.73	471.84	25.33	17.21
0N3	5	462.96	473.14	26.09	15.92
0N4	6	458.09	470.34	30.96	18.72
0CN1	4	463.57	471.69	25.48	17.37
0CN2	5	458.65	468.82	30.40	20.23
0CN3	6	458.29	470.54	30.76	18.52
0CN4	7	456.74	471.07	32.31	17.98
0E1	4	462.53	470.64	26.53	18.41
0E2	5	457.65	467.82	31.41	21.23
0E3	6	457.03	469.28	32.02	19.78
0CE1	5	459.87	470.04	29.18	19.01
0CE2	6	457.05	469.30	32.00	19.76
0CE3	7	456.23	470.56	32.83	18.50
1	3	454.23	460.30	34.83	28.76
1C	4	453.22	461.34	35.84	27.72
1N1	4	452.48	460.59	36.58	28.46
1N2	5	449.03	459.21	40.02	29.85
1N3	6	448.96	461.21	40.09	27.85
1N4	7	448.12	462.44	40.94	26.61
1CN1	5	451.08	461.25	37.98	27.80
1CN2	6	447.20	459.45	41.85	29.61
1CN3	7	447.12	461.45	41.93	27.60
1CN4	8	446.27	462.69	42.78	26.36

1E1	5	450.07	460.25	38.98	28.80
1E2	6	447.81	460.05	41.25	29.00
1E3	7	447.13	461.46	41.93	27.60
1CE1	6	448.84	461.08	40.22	27.97
1CE2	7	446.67	461.00	42.38	28.05
1CE3	8	446.09	462.51	42.96	26.54
2	4	***449.84***	***457.96***	***39.21***	***31.10***
2C	5	449.19	459.37	39.86	29.69
2N1	5	448.52	458.69	40.54	30.36
2N2	6	445.87	458.11	43.19	30.94
2N3	7	445.78	460.11	43.27	28.94
2N4	8	445.13	461.56	43.92	27.50
2CN1	6	447.07	459.32	41.98	29.74
2CN2	7	444.24	458.57	44.82	30.49
2CN3	8	444.07	460.50	44.98	28.56
2CN4	9	443.48	462.02	45.57	27.04
2E1	6	447.27	459.51	41.79	29.54
2E2	7	444.82	459.15	44.23	29.90
2E3	8	444.39	460.81	44.67	28.24
2CE1	7	446.10	460.42	42.96	28.63
2CE2	8	443.71	460.13	45.35	28.92
2CE3	9	443.15	461.68	45.91	27.38

Table M. Discriminability of the no-bias model "2" with only GRL: 3-T Face/House version (Poor-learner group). See Fig E.

3-T Face/House “2” simulation		Nonlearner (n = 7)			
		Absolute		Residual	
Model	df	Dev.	AICc	Dev.	AICc
X	0	481.64	481.64	0	0
XC	1	480.58	482.59	1.06	-0.96
XN1	1	479.89	481.90	1.75	-0.26
XN2	2	478.34	482.38	3.30	-0.74
XN3	3	476.67	482.74	4.96	-1.11
XN4	4	473.76	481.88	7.87	-0.24
XN5	5	473.76	483.94	7.87	-2.30
XN6	6	473.76	486.01	7.87	-4.37
XN7	7	473.75	488.09	7.89	-6.45
XN8	8	472.41	488.84	9.22	-7.21
XCN1	2	478.29	482.32	3.35	-0.68
XCN2	3	477.26	483.33	4.38	-1.69
XCN3	4	474.76	482.88	6.88	-1.24
XCN4	5	473.13	483.30	8.51	-1.67
XCN5	6	473.13	483.38	8.51	-3.74
XCN6	7	473.13	487.46	8.51	-5.82
XCN7	8	472.77	489.20	8.87	-7.56
XCN8	9	471.84	490.37	9.80	-8.74
XE1	2	478.06	482.09	3.58	-0.45
XE2	3	476.73	482.81	4.90	-1.17
XE3	4	475.60	483.72	6.03	-2.08
XCE1	3	477.37	483.44	4.27	-1.80
XCE2	4	476.11	484.22	5.53	-2.59
XCE3	5	474.60	484.78	7.04	-3.14

0	2	***470.88***	***474.91***	***10.76***	***6.73***
0C	3	469.82	475.89	11.82	5.75
0N1	3	470.15	476.22	11.49	5.42
0N2	4	467.97	476.09	13.66	5.55
0N3	5	466.73	476.91	14.91	4.73
0N4	6	464.97	477.22	16.67	4.42
0CN1	4	468.75	476.86	12.89	4.77
0CN2	5	466.96	477.14	14.68	4.50
0CN3	6	465.75	478.00	15.89	3.64
0CN4	7	464.60	478.94	17.03	2.70
0E1	4	467.90	476.02	13.74	5.62
0E2	5	465.97	476.15	15.66	5.49
0E3	6	465.07	477.32	16.56	4.32
0CE1	5	466.57	476.74	15.07	4.89
0CE2	6	465.29	477.54	16.35	4.10
0CE3	7	464.47	478.81	17.17	2.83
1	3	468.87	474.94	12.77	6.70
1C	4	467.99	476.11	13.65	5.53
1N1	4	468.13	476.24	13.51	5.39
1N2	5	466.71	476.89	14.92	4.75
1N3	6	465.68	477.93	15.96	3.71
1N4	7	464.61	478.94	17.03	2.69
1CN1	5	466.84	477.02	14.80	4.62
1CN2	6	465.86	478.11	15.78	3.53
1CN3	7	465.00	479.34	16.64	2.30
1CN4	8	464.23	480.66	17.41	0.98

1E1	5	465.58	475.76	16.05	5.88
1E2	6	464.55	476.80	17.09	4.84
1E3	7	463.35	477.68	18.29	3.96
1CE1	6	465.12	477.37	16.52	4.27
1CE2	7	463.58	477.92	18.06	3.72
1CE3	8	462.90	479.33	18.74	2.31
2	4	468.80	476.92	12.83	4.72
2C	5	467.92	478.09	13.72	3.54
2N1	5	467.98	478.15	13.66	3.48
2N2	6	466.65	478.90	14.99	2.74
2N3	7	465.67	480.00	15.97	1.64
2N4	8	464.60	481.04	17.03	0.60
2CN1	6	466.75	479.00	14.89	2.64
2CN2	7	465.83	480.16	15.81	1.47
2CN3	8	464.97	481.40	16.66	0.23
2CN4	9	464.22	482.76	17.42	-1.12
2E1	6	465.50	477.75	16.13	3.88
2E2	7	464.47	478.80	17.17	2.83
2E3	8	463.19	479.62	18.45	2.02
2CE1	7	465.04	479.38	16.59	2.26
2CE2	8	463.42	479.85	18.22	1.79
2CE3	9	462.75	481.29	18.89	0.35

Table N. Discriminability of the no-bias model “2” with only GRL: 3-T Face/House version (Nonlearner group). See Fig E.

7-T Color/Motion "2" simulation		Good learner (n = 16)			
		Absolute		Residual	
Model	df	Dev.	AICc	Dev.	AICc
X	0	478.79	478.79	0	0
XC	1	476.49	478.50	2.30	0.29
XN1	1	475.89	477.90	2.90	0.89
XN2	2	474.14	478.17	4.65	0.62
XN3	3	471.66	477.73	7.13	1.06
XN4	4	469.55	477.67	9.24	1.12
XN5	5	469.34	479.52	9.45	-0.73
XN6	6	469.13	481.38	9.66	-2.59
XN7	7	468.71	483.05	10.08	-4.26
XN8	8	467.54	483.98	11.25	-5.19
XCN1	2	473.58	477.62	5.21	1.17
XCN2	3	472.32	478.40	6.47	0.40
XCN3	4	470.64	478.76	8.15	0.03
XCN4	5	468.11	478.29	10.68	0.50
XCN5	6	467.90	480.15	10.89	-1.36
XCN6	7	467.69	482.03	11.10	-3.23
XCN7	8	467.22	483.65	11.57	-4.86
XCN8	9	466.18	484.72	12.61	-5.93
XE1	2	473.31	477.34	5.48	1.45
XE2	3	471.76	477.83	7.03	0.96
XE3	4	469.50	477.62	9.29	1.17
XCE1	3	472.20	478.27	6.59	0.52
XCE2	4	470.91	479.02	7.89	-0.23
XCE3	5	468.92	479.10	9.87	-0.31

0	2	445.87	449.90	32.93	28.89
0C	3	437.26	443.33	41.53	35.46
0N1	3	443.48	449.55	35.31	29.24
0N2	4	419.64	427.76	59.15	51.03
0N3	5	418.76	428.94	60.03	49.85
0N4	6	417.75	430.00	61.04	48.79
0CN1	4	427.34	435.46	51.45	43.33
0CN2	5	418.17	428.35	60.62	50.44
0CN3	6	417.48	429.73	61.31	49.06
0CN4	7	416.47	430.80	62.33	47.99
0E1	4	432.89	441.01	45.90	37.78
0E2	5	417.65	427.83	61.14	50.96
0E3	6	415.90	428.16	62.89	50.64
0CE1	5	418.02	428.20	60.77	50.59
0CE2	6	416.32	428.57	62.47	50.22
0CE3	7	415.13	429.47	63.66	49.32
1	3	433.54	439.62	45.25	39.18
1C	4	412.00	420.12	66.79	58.67
1N1	4	421.16	429.28	57.63	49.51
1N2	5	406.95	417.13	71.84	61.66
1N3	6	406.07	418.32	72.73	60.47
1N4	7	404.92	419.26	73.87	59.54
1CN1	5	407.40	417.58	71.39	61.21
1CN2	6	405.55	417.80	73.24	60.99
1CN3	7	405.03	419.37	73.76	59.42
1CN4	8	403.99	420.43	74.80	58.37

1E1	5	408.10	418.28	70.69	60.51
1E2	6	405.27	417.52	73.52	61.27
1E3	7	403.61	417.95	75.18	60.85
1CE1	6	405.80	418.05	72.99	60.74
1CE2	7	403.78	418.11	75.01	60.68
1CE3	8	402.93	419.36	75.87	59.43
2	4	***404.12***	***412.24***	***75.67***	***66.55***
2C	5	402.53	412.71	76.26	66.08
2N1	5	402.49	412.67	76.30	66.12
2N2	6	400.85	413.10	77.94	65.69
2N3	7	400.26	414.59	78.54	64.20
2N4	8	399.20	415.63	79.59	63.16
2CN1	6	401.17	413.42	77.62	65.37
2CN2	7	399.83	414.16	78.96	64.63
2CN3	8	399.43	415.87	79.36	62.93
2CN4	9	398.50	417.04	80.29	61.75
2E1	6	401.20	413.45	77.59	65.34
2E2	7	399.76	414.10	79.03	64.69
2E3	8	398.14	414.57	80.65	64.22
2CE1	7	400.21	414.55	78.58	64.24
2CE2	8	398.34	414.77	80.46	64.02
2CE3	9	397.54	416.08	81.25	62.71

Table O. Discriminability of the no-bias model "2" with only GRL: 7-T Color/Motion version (Good-learner group). See Fig F.

7-T Color/Motion "2" simulation		Poor learner (*n* = 5)			
		Absolute		Residual	
Model	df	Dev.	AICc	Dev.	AICc
X	0	447.50	447.50	0	0
XC	1	445.68	447.69	1.82	-0.20
XN1	1	445.34	447.35	2.16	0.14
XN2	2	444.53	448.57	2.97	-1.07
XN3	3	442.43	448.51	5.06	-1.02
XN4	4	439.56	447.70	7.93	-0.20
XN5	5	439.56	449.77	7.93	-2.27
XN6	6	439.51	451.79	7.99	-4.30
XN7	7	439.46	453.84	8.03	-6.34
XN8	8	438.16	454.64	9.34	-7.15
XCN1	2	444.30	448.34	3.20	-0.84
XCN2	3	443.30	449.38	4.20	-1.88
XCN3	4	440.29	448.43	7.20	-0.93
XCN4	5	439.16	449.36	8.34	-1.86
XCN5	6	439.16	451.44	8.34	-3.94
XCN6	7	439.14	453.51	8.36	-6.02
XCN7	8	437.97	454.46	9.52	-6.97
XCN8	9	437.79	456.40	9.71	-8.90
XE1	2	444.10	448.14	3.39	-0.65
XE2	3	442.46	448.54	5.04	-1.04
XE3	4	439.53	447.67	7.96	-0.17
XCE1	3	442.92	449.00	4.58	-1.50
XCE2	4	440.24	448.37	7.26	-0.88
XCE3	5	438.82	449.02	8.68	-1.52

0	2	437.40	441.44	10.10	6.06
0C	3	435.87	441.95	11.63	5.55
0N1	3	435.68	441.76	11.81	5.73
0N2	4	433.00	441.14	14.49	6.36
0N3	5	432.66	442.86	14.84	4.64
0N4	6	431.36	443.65	16.13	3.85
0CN1	4	434.74	442.87	12.76	4.62
0CN2	5	432.26	442.47	15.23	5.03
0CN3	6	431.09	443.37	16.40	4.12
0CN4	7	430.84	445.21	16.66	2.28
0E1	4	433.16	441.29	14.34	6.20
0E2	5	431.60	441.81	15.89	5.69
0E3	6	429.42	441.70	18.08	5.79
0CE1	5	432.48	442.68	15.01	4.81
0CE2	6	429.62	441.90	17.88	5.59
0CE3	7	429.00	443.38	18.50	4.12
1	3	426.41	432.49	21.08	15.00
1C	4	422.69	430.82	24.81	16.67
1N1	4	425.44	433.57	22.06	13.92
1N2	5	421.73	431.93	25.77	15.56
1N3	6	421.10	433.38	26.40	14.12
1N4	7	417.74	432.11	29.76	15.38
1CN1	5	422.01	432.22	25.48	15.28
1CN2	6	418.82	431.10	28.68	16.40
1CN3	7	417.91	432.29	29.58	15.20
1CN4	8	415.72	432.21	31.78	15.29

1E1	5	418.47	428.67	29.03	18.83
1E2	6	***416.16***	***428.44***	***31.34***	***19.05***
1E3	7	414.38	428.76	33.12	18.74
1CE1	6	416.42	428.71	31.07	18.79
1CE2	7	414.38	428.76	33.11	18.73
1CE3	8	414.01	430.50	33.49	17.00
2	4	423.36	431.49	24.14	16.00
2C	5	422.06	432.26	25.43	15.23
2N1	5	418.67	428.87	28.82	18.62
2N2	6	417.26	429.54	30.24	17.96
2N3	7	416.72	431.09	30.78	16.40
2N4	8	415.49	431.98	32.01	15.52
2CN1	6	417.78	430.06	29.72	17.44
2CN2	7	416.56	430.94	30.93	16.55
2CN3	8	415.60	432.09	31.90	15.41
2CN4	9	414.88	433.49	32.62	14.00
2E1	6	416.59	428.87	30.91	18.62
2E2	7	415.47	429.84	32.03	17.65
2E3	8	413.54	430.03	33.96	17.47
2CE1	7	415.76	430.13	31.74	17.36
2CE2	8	413.56	430.05	33.94	17.45
2CE3	9	413.22	431.83	34.28	15.67

Table P. Discriminability of the no-bias model “2” with only GRL: 7-T Color/Motion version (Poor-learner group). See Fig F.

3-T Face/House		Good learner (*n* = 31)			
Empirical data		Absolute		Residual	
Model	df	Dev.	AICc	Dev.	AICc
State-independent action hysteresis					
2	4	431.38	439.50	69.88	61.76
2N1	5	425.20	435.37	76.06	65.89
2N2	6	424.09	436.33	77.16	64.92
2E1	6	422.81	435.05	78.45	66.21
2C	5	426.66	436.83	74.59	64.42
2CN1	6	421.78	434.02	79.48	67.24
2CN2	7	420.53	434.86	80.72	66.40
2CE1	7	***419.25***	***433.57***	***82.01***	***67.69***
State-dependent action hysteresis					
2	4	431.38	439.50	69.88	61.76
2sN1	5	427.55	437.72	73.71	63.54
2sN2	6	425.43	437.67	75.83	63.58
2sE1	6	421.88	434.12	79.38	67.14
2C	5	426.66	436.83	74.59	64.42
2CsN1	6	422.91	435.15	78.35	66.10
2CsN2	7	420.86	435.18	80.40	66.07
2CsE1	7	***419.47***	***433.79***	***81.79***	***67.47***
State-independent + State-dependent action hysteresis					
sE1+2	6	421.88	434.12	79.38	67.14
sE1+2N1	7	417.45	431.78	83.80	69.48
sE1+2N2	8	416.39	432.80	84.87	68.46
sE1+2E1	8	415.05	431.46	86.21	69.80
sE1+2C	7	419.47	433.79	81.79	67.47

sE1+2CN1	8	414.97	431.38	86.29	69.87
sE1+2CN2	9	413.70	432.22	87.56	69.04
sE1+2CE1	9	***411.60****	***430.13****	***89.65****	***71.13****
State-independent hysteresis + State-indep. action value					
Qa+2	6	428.56	440.80	72.69	60.45
Qa+2N1	7	424.24	438.56	77.01	62.69
Qa+2N2	8	423.08	439.50	78.17	61.76
Qa+2E1	8	421.53	437.95	79.72	63.31
Qa+2C	7	424.94	439.26	76.32	62.00
Qa+2CN1	8	420.63	437.04	80.63	64.22
Qa+2CN2	9	419.35	437.87	81.91	63.39
Qa+2CE1	9	***417.81***	***436.34***	***83.44***	***64.92***
State-independent hysteresis + Confirmation bias					
cLR+2	5	428.64	438.81	72.62	62.45
cLR+2N1	6	422.68	434.92	78.58	66.34
cLR+2N2	7	421.43	435.75	79.83	65.51
cLR+2E1	7	420.14	434.46	81.12	66.80
cLR+2C	6	423.49	435.73	77.77	65.53
cLR+2CN1	7	419.07	433.39	82.19	67.87
cLR+2CN2	8	417.79	434.20	83.47	67.06
cLR+2CE1	8	***416.52***	***432.94***	***84.74***	***68.32***
State-independent hysteresis + Asymmetric learning rates					
LR+2	5	425.79	435.97	75.46	65.29
LR+2N1	6	421.37	433.61	79.89	67.65
LR+2N2	7	420.09	434.41	81.17	66.85
LR+2E1	7	418.85	433.17	82.40	68.08
LR+2C	6	422.33	434.58	78.92	66.68

LR+2CN1	7	417.84	432.16	83.42	69.09
LR+2CN2	8	416.55	432.97	84.71	68.29
LR+2CE1	8	***415.27***	***431.69***	***85.99***	***69.57***

Table Q. Extended model comparison: 3-T Face/House version (Good-learner group). See Table 4. Results with the best fit within each subset of 8 models are highlighted with boldface and italics. Results with the best fit across all 44 models are also marked with asterisks.

3-T Face/House		Poor learner (*n* = 9)			
Empirical data		Absolute		Residual	
Model	df	Dev.	AICc	Dev.	AICc
State-independent action hysteresis					
2	4	466.69	474.80	22.37	14.25
2N1	5	447.10	457.28	41.95	31.77
2N2	6	437.29	449.54	51.76	39.52
2E1	6	425.45	437.70	63.60	51.35
2C	5	432.05	442.23	57.00	46.82
2CN1	6	425.47	437.72	63.58	51.34
2CN2	7	422.92	437.25	66.13	51.81
2CE1	7	***420.67***	***435.00***	***68.39***	***54.06***
State-dependent action hysteresis					
2	4	466.69	474.80	22.37	14.25
2sN1	5	435.25	445.43	53.80	43.63
2sN2	6	422.66	434.90	66.40	54.15
2sE1	6	417.89	430.14	71.17	58.92
2C	5	432.05	442.23	57.00	46.82
2CsN1	6	420.17	432.42	68.88	56.64
2CsN2	7	413.49	427.82	75.56	61.23
2CsE1	7	***412.33***	***426.66***	***76.73***	***62.40***
State-independent + State-dependent action hysteresis					
sE1+2	6	417.89	430.14	71.17	58.92
sE1+2N1	7	413.19	427.52	75.87	61.54
sE1+2N2	8	411.48	427.91	77.57	61.15
sE1+2E1	8	410.06	426.49	78.99	62.57
sE1+2C	7	412.33	426.66	76.73	62.40

sE1+2CN1	8	***407.13****	***423.55****	***81.93****	***65.50****
sE1+2CN2	9	405.13	423.66	83.93	65.39
sE1+2CE1	9	405.57	424.10	83.48	64.95
State-independent hysteresis + State-indep. action value					
Qa+2	6	460.14	472.38	28.92	16.67
Qa+2N1	7	446.54	460.87	42.51	28.18
Qa+2N2	8	436.60	453.02	52.46	36.03
Qa+2E1	8	424.86	441.29	64.19	47.77
Qa+2C	7	430.46	444.79	58.59	44.26
Qa+2CN1	8	424.91	441.33	64.15	47.72
Qa+2CN2	9	422.34	440.87	66.71	48.18
Qa+2CE1	9	***420.20***	***438.74***	***68.85***	***50.32***
State-independent hysteresis + Confirmation bias					
cLR+2	5	441.61	451.79	47.44	37.27
cLR+2N1	6	434.37	446.62	54.68	42.44
cLR+2N2	7	431.22	445.55	57.83	43.51
cLR+2E1	7	422.59	436.92	66.46	52.13
cLR+2C	6	426.61	438.86	62.44	50.19
cLR+2CN1	7	421.57	435.90	67.49	53.16
cLR+2CN2	8	419.39	435.82	69.66	53.23
cLR+2CE1	8	***417.96***	***434.39***	***71.09***	***54.67***
State-independent hysteresis + Asymmetric learning rates					
LR+2	5	441.58	451.76	47.47	37.29
LR+2N1	6	434.35	446.60	54.70	42.45
LR+2N2	7	431.21	445.54	57.84	43.51
LR+2E1	7	422.44	436.77	66.62	52.29
LR+2C	6	426.57	438.81	62.49	50.24

LR+2CN1	7	421.56	435.88	67.50	53.17
LR+2CN2	8	419.39	435.81	69.67	53.24
LR+2CE1	8	***417.90***	***434.32***	***71.15***	***54.73***

Table R. Extended model comparison: 3-T Face/House version (Poor-learner group).
See Table 4.

3-T Face/House		Nonlearner (n = 7)			
Empirical data		Absolute		Residual	
Model	df	Dev.	AICc	Dev.	AICc
State-independent action hysteresis					
2	4	477.44	485.55	4.20	-3.91
2N1	5	463.53	473.71	18.11	7.93
2N2	6	457.80	470.05	23.84	11.59
2E1	6	455.81	468.06	25.82	13.58
2C	5	465.74	475.92	15.90	5.72
2CN1	6	455.60	467.85	26.04	13.79
2CN2	7	451.35	465.68	30.29	15.95
2CE1	7	***451.26****	***465.60****	***30.37****	***16.04****
State-dependent action hysteresis					
2	4	477.44	485.55	4.20	-3.91
2sN1	5	471.16	481.33	10.48	0.30
2sN2	6	467.23	479.48	14.41	2.16
2sE1	6	464.49	476.74	17.15	4.90
2C	5	465.74	475.92	15.90	5.72
2CsN1	6	463.93	476.18	17.71	5.46
2CsN2	7	461.77	476.10	19.87	5.54
2CsE1	7	***461.37***	***475.70***	***20.27***	***5.94***
State-independent + State-dependent action hysteresis					
sE1+2	6	464.49	476.74	17.15	4.90
sE1+2N1	7	455.33	469.67	26.30	11.97
sE1+2N2	8	451.97	468.40	29.67	13.24
sE1+2E1	8	451.43	467.86	30.21	13.78
sE1+2C	7	461.37	475.70	20.27	5.94

sE1+2CN1	8	450.79	467.22	30.85	14.42
sE1+2CN2	9	447.67	466.21	33.97	15.43
sE1+2CE1	9	***447.14***	***465.68***	***34.50***	***15.96***
State-independent hysteresis + State-indep. action value					
Qa+2	6	474.29	486.54	7.35	-4.90
Qa+2N1	7	459.51	473.84	22.13	7.80
Qa+2N2	8	454.95	471.38	26.69	10.26
Qa+2E1	8	452.44	468.87	29.20	12.77
Qa+2C	7	462.51	476.85	19.12	4.79
Qa+2CN1	8	452.67	469.10	28.97	12.54
Qa+2CN2	9	448.66	467.20	32.98	14.44
Qa+2CE1	9	***448.28***	***466.82***	***33.36***	***14.82***
State-independent hysteresis + Confirmation bias					
cLR+2	5	473.61	483.79	8.03	-2.15
cLR+2N1	6	462.11	474.36	19.53	7.28
cLR+2N2	7	457.47	471.81	24.17	9.83
cLR+2E1	7	454.77	469.10	26.87	12.54
cLR+2C	6	465.52	477.77	16.12	3.87
cLR+2CN1	7	455.06	469.39	26.58	12.25
cLR+2CN2	8	451.16	467.59	30.48	14.05
cLR+2CE1	8	***450.78***	***467.21***	***30.86***	***14.43***
State-independent hysteresis + Asymmetric learning rates					
LR+2	5	473.60	483.78	8.04	-2.14
LR+2N1	6	461.97	474.21	19.67	7.42
LR+2N2	7	457.19	471.53	24.45	10.11
LR+2E1	7	454.48	468.82	27.16	12.82
LR+2C	6	465.41	477.65	16.23	3.98

LR+2CN1	7	455.05	469.39	26.59	12.25
LR+2CN2	8	450.96	467.39	30.68	14.25
LR+2CE1	8	***450.49***	***466.92***	***31.15***	***14.72***

Table S. Extended model comparison: 3-T Face/House version (Nonlearner group). See Table 4.

7-T Color/Motion<br>Empirical data<br>Model	df	Good learner (n = 16)<br>Absolute<br>Dev.	 <br>AICc	 Residual<br>Dev.	 <br>AICc
State-independent action hysteresis					
2	4	415.73	423.85	63.06	54.94
2N1	5	405.96	416.13	72.84	62.66
2N2	6	404.04	416.29	74.75	62.50
2E1	6	403.02	415.27	75.77	63.52
2C	5	403.95	414.13	74.85	64.67
2CN1	6	399.09	411.34	79.71	67.45
2CN2	7	397.72	412.06	81.07	66.73
2CE1	7	***396.84***	***411.17***	***81.95***	***67.62***
State-dependent action hysteresis					
2	4	415.73	423.85	63.06	54.94
2sN1	5	405.15	415.33	73.64	63.46
2sN2	6	403.41	415.66	75.38	63.13
2sE1	6	397.47	409.72	81.32	69.07
2C	5	403.95	414.13	74.85	64.67
2CsN1	6	400.65	412.91	78.14	65.89
2CsN2	7	399.08	413.42	79.71	65.37
2CsE1	7	***394.52***	***408.86***	***84.27***	***69.93***
State-independent + State-dependent action hysteresis					
sE1+2	6	397.47	409.72	81.32	69.07
sE1+2N1	7	394.16	408.49	84.63	70.30
sE1+2N2	8	393.10	409.54	85.69	69.25
sE1+2E1	8	392.26	408.70	86.53	70.09
sE1+2C	7	394.52	408.86	84.27	69.93

sE1+2CN1	8	390.10	406.53	88.69	72.26
sE1+2CN2	9	388.98	407.52	89.81	71.27
sE1+2CE1	9	***387.61****	***406.15****	***91.18****	***72.64****
State-independent hysteresis + State-indep. action value					
Qa+2	6	407.40	419.65	71.39	59.14
Qa+2N1	7	403.45	417.79	75.34	61.00
Qa+2N2	8	401.70	418.14	77.09	60.65
Qa+2E1	8	400.66	417.10	78.13	61.69
Qa+2C	7	401.97	416.31	76.82	62.48
Qa+2CN1	8	396.64	413.07	82.15	65.72
Qa+2CN2	9	395.23	413.78	83.56	65.01
Qa+2CE1	9	***394.18***	***412.72***	***84.62***	***66.07***
State-independent hysteresis + Confirmation bias					
cLR+2	5	406.87	417.05	71.92	61.74
cLR+2N1	6	403.88	416.13	74.91	62.66
cLR+2N2	7	402.31	416.64	76.49	62.15
cLR+2E1	7	401.36	415.69	77.44	63.10
cLR+2C	6	402.15	414.40	76.64	64.39
cLR+2CN1	7	397.52	411.85	81.28	66.94
cLR+2CN2	8	396.19	412.63	82.60	66.17
cLR+2CE1	8	***395.30***	***411.74***	***83.49***	***67.06***
State-independent hysteresis + Asymmetric learning rates					
LR+2	5	406.31	416.49	72.48	62.30
LR+2N1	6	403.33	415.59	75.46	63.21
LR+2N2	7	401.65	415.99	77.14	62.80
LR+2E1	7	400.80	415.14	77.99	63.66
LR+2C	6	401.75	414.00	77.04	64.79

LR+2CN1	7	397.08	411.42	81.71	67.37
LR+2CN2	8	395.67	412.10	83.12	66.69
LR+2CE1	8	***394.81***	***411.24***	***83.98***	***67.55***

Table T. Extended model comparison: 7-T Color/Motion version (Good-learner group). See Table 4.

7-T Color/Motion		Poor learner (n = 5)			
Empirical data		Absolute		Residual	
Model	df	Dev.	AICc	Dev.	AICc
State-independent action hysteresis					
2	4	426.37	434.51	21.12	12.99
2N1	5	402.03	412.23	45.47	35.27
2N2	6	392.33	404.61	55.16	42.88
2E1	6	390.92	403.20	56.58	44.29
2C	5	415.89	426.09	31.60	21.40
2CN1	6	393.88	406.16	53.61	41.33
2CN2	7	***384.97***	***399.34***	***62.53***	***48.15***
2CE1	7	386.62	401.00	60.88	46.50
State-dependent action hysteresis					
2	4	426.37	434.51	21.12	12.99
2sN1	5	423.69	433.89	23.80	13.60
2sN2	6	420.67	432.95	26.82	14.54
2sE1	6	415.54	427.82	31.96	19.68
2C	5	415.89	426.09	31.60	21.40
2CsN1	6	413.73	426.01	33.76	21.48
2CsN2	7	411.56	425.94	35.93	21.55
2CsE1	7	***410.09***	***424.47***	***37.40***	***23.02***
State-independent + State-dependent action hysteresis					
sE1+2	6	415.54	427.82	31.96	19.68
sE1+2N1	7	393.56	407.94	53.94	39.56
sE1+2N2	8	384.64	401.13	62.86	46.37
sE1+2E1	8	385.78	402.27	61.72	45.23
sE1+2C	7	410.09	424.47	37.40	23.02

sE1+2CN1	8	388.61	405.10	58.88	42.39
sE1+2CN2	9	***379.59***	***398.20***	***67.91***	***49.30***
sE1+2CE1	9	381.68	400.29	65.82	47.21
State-independent hysteresis + State-indep. action value					
Qa+2	6	417.29	429.57	30.21	17.93
Qa+2N1	7	396.52	410.90	50.98	36.60
Qa+2N2	8	386.89	403.38	60.60	44.11
Qa+2E1	8	385.33	401.81	62.17	45.68
Qa+2C	7	407.07	421.45	40.43	26.05
Qa+2CN1	8	388.04	404.52	59.46	42.97
Qa+2CN2	9	***378.86****	***397.48****	***68.63****	***50.02****
Qa+2CE1	9	380.76	399.37	66.74	48.12
State-independent hysteresis + Confirmation bias					
cLR+2	5	424.12	434.32	23.38	13.18
cLR+2N1	6	401.39	413.67	46.11	33.82
cLR+2N2	7	392.12	406.49	55.38	41.00
cLR+2E1	7	390.82	405.20	56.68	42.30
cLR+2C	6	415.57	427.85	31.93	19.64
cLR+2CN1	7	393.83	408.21	53.66	39.28
cLR+2CN2	8	***384.96***	***401.45***	***62.54***	***46.05***
cLR+2CE1	8	386.54	403.03	60.95	44.47
State-independent hysteresis + Asymmetric learning rates					
LR+2	5	423.45	433.65	24.05	13.85
LR+2N1	6	400.81	413.09	46.69	34.41
LR+2N2	7	391.47	405.85	56.02	41.65
LR+2E1	7	390.13	404.51	57.36	42.99
LR+2C	6	414.89	427.17	32.60	20.32

LR+2CN1	7	393.17	407.55	54.32	39.95
LR+2CN2	8	***384.23***	***400.72***	***63.27***	***46.78***
LR+2CE1	8	385.85	402.34	61.64	45.16

Table U. Extended model comparison: 7-T Color/Motion version (Poor-learner group). See Table 4.
